# Brain region-resolved pharmacodynamics of an antisense oligonucleotide targeting human *SNCA* 3′UTR in BAC-h*SNCA* rats

**DOI:** 10.1186/s40035-026-00577-x

**Published:** 2026-09-16

**Authors:** Marianna Naki, Fedon-Giasin Kattan, Marina Pantazopoulou, Sissy Skea, Dimitrios Zisis, Eleftherios Pilalis, Maria Fouka, Effrosyni Koronaiou, Christos Fotis, Olaf Riess, Aristotelis Chatziioannou, Leonidas G. Alexopoulos, Leonidas Stefanis, Epaminondas Doxakis

**Affiliations:** 1 Center of Basic Research, Biomedical Research Foundation of the Academy of Athens, Athens, 11527 Greece; 2 Department of Physiology, National and Kapodistrian University of Athens (NKUA), Athens, 11527 Greece; 3 Center of Clinical Research, Biomedical Research Foundation of the Academy of Athens, Athens, 11527 Greece; 4 Protavio Ltd, Demokritos Science Park, Agia Paraskevi, 15341 Greece; 5 e-NIOS Applications PC, Kallithea, 17671 Greece; 6 Institute of Medical Genetics and Applied Genomics, University of Tübingen, Tübingen, 72076 Germany; 7 Centers of Systems Biology, Biomedical Research Foundation, Academy of Athens, Athens, 11527 Greece; 8 Biomedical Systems Laboratory, National Technical University of Athens, Athens, 15780 Greece; 9 First Department of Neurology, National and Kapodistrian University of Athens Medical School, Athens, 11528 Greece

**Keywords:** Alpha-synuclein (SNCA), Parkinson's disease, Antisense oligonucleotide, BAC transgenic rat, 3′UTR targeting, Neuroinflammation, Prodromal intervention

## Abstract

**Background:**

Alpha-synuclein accumulation contributes to Parkinson’s disease (PD), and lowering α-synuclein expression is a candidate disease-modifying approach. The brain-wide pharmacodynamic profile of human-targeting antisense oligonucleotides (ASOs) remains incompletely defined in humanized models that preserve the regulatory architecture of the human *SNCA* locus.

**Methods:**

BAC-h*SNCA* rats were treated at an early stage, when human α-synuclein is elevated, but striatal dopamine content remains largely preserved. A single intracerebroventricular dose of an ASO targeting human *SNCA* 3′UTR (SNCA ASO3.0) was administered, and outcomes were assessed 45 days later by isoform-specific RT-qPCR, immunoblotting of soluble and detergent-insoluble α-synuclein species, bulk RNA-seq across selected regions, multiplex cytokine profiling, phospho-kinase assays, neurotransmitter quantification, and behavioral testing.

**Results:**

SNCA ASO3.0 reduced human *SNCA* mRNA and multiple α-synuclein protein species in a regionally graded manner, with the strongest effects in the olfactory bulb and striatum. Bulk RNA-seq identified anatomically distinct transcriptional responses, including synaptic and metabolic pathway shifts in the olfactory bulbs and immune-related enrichment within glial signatures in the striatum and midbrain, without detectable changes in protein levels of glial fibrillary acidic protein or ionized calcium-binding adapter molecule 1. Cytokine profiling showed selective modulation of a subset of analytes, and phospho-kinase assays indicated reduced AKT1S1/PRAS40 phosphorylation. Striatal dopamine levels were unchanged, whereas the striatal tyrosine hydroxylase level was increased. SNCA ASO3.0 increased locomotor activity and improved exploratory behavior and selected measures of olfactory discrimination.

**Conclusions:**

Partial suppression of human α-synuclein in a humanized rat model yields region-specific molecular adaptations accompanied by early behavioral changes at a stage preceding overt dopamine depletion. These data provide a framework for evaluating region-resolved consequences of α-synuclein-lowering interventions in synucleinopathies.

**Supplementary Information:**

The online version contains supplementary material available at https://doi.org/10.1186/s40035-026-00577-x.

## Background

Alpha-synuclein (encoded by *SNCA*) is a presynaptic protein involved in synaptic vesicle trafficking, membrane remodeling, and neurotransmitter release in neurons [1]. Increased *SNCA* dosage, caused by point mutations, gene duplications, or non-coding regulatory variants, contributes to Parkinson’s disease (PD) risk and drives the accumulation of soluble and aggregated forms of the protein in vulnerable brain regions [2–5]. Phosphorylated α-synuclein at Ser129 is a major component of Lewy bodies and Lewy neurites and is widely used as a marker of α-synuclein pathology [6]. Reducing α-synuclein expression has therefore become a rational therapeutic strategy. Antisense oligonucleotides targeting *SNCA* can lower the mRNA and protein levels of α-synuclein in rodent and non-human primate brains and reduce pathological burden in preclinical models [7, 8]. These studies established feasibility, yet how partial reduction of human *SNCA* affects distinct brain regions remains poorly defined, particularly in the early stages when synaptic and transcriptional adaptations precede overt neurodegeneration. Defining region-specific responses is essential, because ASO distribution, α-synuclein turnover, and local susceptibility differ markedly across brain structures relevant to PD [9].

Human *SNCA* transcripts undergo alternative polyadenylation and generate multiple 3′UTR isoforms with distinct regulatory properties [10–12]. Reported isoforms span from approximately 290 to 2529 nucleotides (nt) [11, 12], and a 1246 nt extension has been detected at lower abundance [10]. Longer 3′UTRs harbor additional RNA-binding protein and microRNA recognition sites, and extended variants exhibit reduced translational efficiency in cellular systems [10, 11]. Transcriptomic datasets from the human cortex report variability in the relative abundance of these isoforms between PD and control tissues [12]. This structural and regulatory diversity provides a rationale for isoform-selective targeting in models expressing the complete human locus.

The BAC-h*SNCA* rat carries the entire human *SNCA* locus, including the promoter and 3′UTR architecture, and expresses all major human isoforms under physiological regulation [13]. This model exhibits progressive accumulation of human α-synuclein, olfactory and exploratory deficits, and later, nigrostriatal dopamine loss, which together constitute cardinal features of synucleinopathy progression. This model provides an opportunity to test human-specific ASOs and to quantify their effects on *SNCA* transcripts, α-synuclein protein species, and downstream pathways across anatomically distinct brain regions.

In this study, BAC-h*SNCA* rats were treated at two months of age and analyzed 45 days later, a stage when human α-synuclein overexpression and early behavioral changes are present, but striatal dopamine content remains largely preserved. Independent transcriptomic analyses in this model have reported that younger animals exhibit a broader set of gene expression changes than older cohorts [14], supporting the view that early stages are transcriptionally active and sensitive to *SNCA*-dependent perturbation. This interval approximates an early symptomatic stage, when hyposmia and subtle behavioral alterations precede clinically manifest PD in patients. Alpha-synuclein-lowering interventions are most likely to be initiated during such early phases rather than at advanced stages with established dopamine loss. Analyses in this study include isoform-specific mRNA quantification, soluble and insoluble α-synuclein species, kinase signaling, cytokines, neurotransmitters, transcriptomic profiling, and behavioral readouts. This integrated dataset addresses how different brain regions respond to *SNCA* knockdown in a humanized background and provides insight into processes that may operate at the initiation phase of Parkinsonian pathology.

## Materials and methods

### Generation of DNA constructs

The coding sequence plus 3′UTRs of human *SNCA* mRNA were PCR-amplified from SK-N-SH neuroblastoma cell cDNA using Phusion polymerase (Thermo Fisher Scientific, F530, Waltham, MA). Amplicons were cloned between the BamHI and HindIII sites of the paavCAG-pre-mGRASP-mCerulean-2A-NLS-mCherry plasmid obtained from Addgene (#34911, Watertown, MA) [15]. All constructs were verified by Sanger sequencing (CeMIA SA, Larisa, Greece). Primer sequences are listed in Additional file 1: Table S1.

### Cell culture and transfection

SK-N-SH neuroblastoma cells were cultured in high-glucose Dulbecco's Modified Eagle Medium (DMEM; Sigma-Aldrich, D6429, St. Louis, MO) supplemented with 10% fetal bovine serum (FBS; Thermo Fisher Scientific, #10270106) and 1% penicillin–streptomycin (Sigma-Aldrich, P4333). Cells were maintained at 37 °C in a humidified 5% CO₂ atmosphere (ThermoForma, Thermo Fisher Scientific). Transfections were performed at the time of plating using 50 or 100 nM oligonucleotides (RNase-free, HPLC-purified; GenScript, Piscataway, NJ and GeneDesign, Osaka, Japan) delivered with jetPRIME reagent (Polyplus, #101000046, Illkirch, France) according to the manufacturer's protocol. Cells were harvested and analyzed 72 h post-transfection.

### Animals

Male Sprague–Dawley BAC-h*SNCA* transgenic rats (Tg(*SNCA*)BAC) [13], aged two months and weighing 300–350 g, were used for the main in vivo experiments. Age-matched wild-type Sprague–Dawley rats were included for selected baseline genotype comparisons, as indicated in the relevant figure legends. Animals were housed in a controlled environment with a 12-h light/dark cycle and ad libitum access to food and water. At 40–45 days post-injection, animals were anesthetized with 3.5%−4% isoflurane through inhalation (Abbott Laboratories, B506, Chicago, IL) and euthanized by decapitation. Brains were rapidly extracted, and defined regions were dissected on ice and snap-frozen at − 80 °C for subsequent analysis. All procedures complied with European Directive 2010/63 on the protection of animals used for scientific purposes.

### Surgical procedures and intracerebroventricular injections

All surgical procedures were performed under isoflurane anesthesia. Intracerebroventricular (i.c.v.) injections were made into the right lateral ventricle using a stereotaxic frame (Kopf Instruments, Tujunga, CA). Coordinates relative to bregma were anterior/posterior (AP) − 0.4 mm, medial/lateral (ML) + 1.4 mm, and dorsal/ventral (DV) − 3.8 mm. A total volume of 15 µL containing 3 mg of ASO in sterile saline was delivered manually at approximately 1 µL/s using a pulled glass capillary (outer diameter: 60–80 µm) connected to a Hamilton syringe fitted with a 22-gauge needle. Following the injection, the capillary was left in place for 5 min to allow diffusion. The scalp incision was closed with three simple interrupted sutures using 3–0 Ethilon (Ethicon, Raritan, NJ). Animals recovered in their home cages under standard conditions and were observed over the first 48 h following i.c.v. dosing for general activity, posture, gait, tremor, and convulsions. Trial injections of Fast Green FCF dye (Thermo Fisher Scientific, A16520) were used as a qualitative control for correct i.c.v. delivery and initial ventricular/parenchymal access of the injectate. Representative sections obtained 30 min after injection are shown in Additional file 1: Fig. S1.

### Behavioral testing

All behavioral assessments were conducted during the light cycle (09:00–17:00) and recorded using EthoVision XT8.5 software (Noldus, Wageningen, Netherlands).

#### Open field test

Male BAC-h*SNCA* rats (*n* = 5 per group; ASO3.0 vs. scrambled ASO1) were placed in a square acrylic arena (40 × 40 × 30 cm^3^) with no external stimuli. Behavior was recorded for 30 min using a ceiling-mounted camera (Panasonic, WV-BP 332EE, Kadoma, Japan). Locomotor activity and time spent in the center of the arena were quantified as indicators of general activity and exploratory behavior, respectively.

#### Olfactory discrimination tests

Three-compartment test and habituation/cross-habituation (HaXha) test were performed. In the three-compartment test, male BAC-h*SNCA* rats (*n* = 5 per group) were placed in a three-compartment arena for a 5-min habituation period. The least visited compartment during habituation was then supplemented with the rat's own bedding to introduce a familiar olfactory cue. The animal was repositioned in the center compartment, and the time spent in each compartment over the subsequent 5 min was recorded to assess exploratory preference (neophilia) and odor recognition.

HaXha testing was performed in an auxiliary female BAC-h*SNCA* cohort (*n* = 3 per group) because only this cohort was available for the assay. Previous studies have reported no estrous cycle-dependent variation in olfactory discrimination [16]. This auxiliary series was not designed to assess sex as a biological variable, and the data are presented as supportive within-cohort evidence only. In each trial, a single odorant was presented for 2 min in a clean test cage while the rat's investigative behavior (defined as the time spent within 0–2 cm of the odorant) was recorded using a side-mounted camera. Odorants included water (baseline), almond, vanilla, and social odor (bedding from an opposite-sex cage). Each stimulus was presented in three consecutive 2-min trials, separated by a 1-min intertrial interval to allow habituation. Behavioral responses to repeated and novel odorants were quantified to assess habituation and discrimination.

### High-performance liquid chromatography (HPLC)

#### Sample preparation and detection

To avoid confounding effects on neurotransmitter levels, animals (*n* = 4 per group; ASO3.0 and scrambled ASO1) were euthanized without the use of volatile anesthetics. Striatal tissue was rapidly dissected, homogenized via sonication in 0.1 M perchloric acid (HClO₄) containing 20 mM TAE buffer (pH 6.1), and centrifuged at 13,000 rpm for 30 min at 4 °C. Supernatants were collected and stored at − 80 °C until analysis.

Neurotransmitters and their metabolites were quantified using reverse-phase ion-pair HPLC with electrochemical detection. Chromatographic separation was performed using an isocratic pump (YL Instrument Co., Ltd., YL9112, Gyeonggi-do, Korea) coupled to an electrochemical detector (BASi, Bioanalytical Systems, Indianapolis, IN; BAS-LC4B) equipped with a glassy carbon working electrode set at + 0.8 V. Peak integration and quantification were performed using Clarity HPLC software (DataApex, Prague, Czech Republic), with calibration against external reference standards. All concentrations were normalized to tissue weight and reported as ng neurotransmitter per mg wet tissue.

#### Monoamine analysis

Striatal supernatants were diluted 1:6 in homogenization buffer and injected into a YMC Triart C18 column (100 × 2 mm, 3 μm particle size; YMC, Shimogyo, Japan). The mobile phase consisted of acetonitrile (Sigma-Aldrich, #271004) in 50 mM phosphate buffer (Thermo Fisher Scientific, #70011036, diluted 1:9 to pH 3.0), supplemented with 300 mg/L 5-octylsulfate sodium salt (Sigma-Aldrich, #O4003) as an ion-pairing reagent and 20 mg/L disodium EDTA (Sigma-Aldrich, #79884). Dopamine (DA), dihydroxyphenylacetic acid (DOPAC), and homovanillic acid (HVA) were resolved and quantified. The flow rate was maintained at 0.2 mL/min, and the detector sensitivity was set to 20 nA.

#### Amino acid analysis

Samples were derivatized with o-phthaldialdehyde (OPA) before injection. The derivatization buffer contained 20 mg of OPA (Sigma-Aldrich, #P0657) dissolved in 5.5 mL of 0.1 M borax buffer with 30 μL of 0.03 M sodium sulfite solution. Amino acids were separated on a PerkinElmer HyperSelect ODS column (250 × 4.6 mm, 5 μm particle size; Thermo Fisher Scientific). The mobile phase consisted of 5% acetonitrile in 0.03 M sodium citrate, 0.13 M disodium hydrogen phosphate dihydrate (NaHPO₄·2H₂O), and 400 mg/L disodium EDTA dihydrate (Na₂EDTA·2H₂O), adjusted to pH 5.3. The flow rate was set to 0.8 mL/min, and the detector sensitivity was set to 20 nA. Quantified analytes included aspartate, glutamate, glutamine, and γ-aminobutyric acid.

### Immunohistochemistry

For immunohistochemical analysis, animals (*n* = 3 per group) were deeply anesthetized with pentobarbital and perfused transcardially with physiological saline, followed by ice-cold 4% paraformaldehyde. Brains were post-fixed overnight in 4% paraformaldehyde at 4 °C, then cryoprotected by sequential immersion in 15% and 30% sucrose solutions (*w*/*v*) for 24 h each. Following freezing at − 80 °C, coronal brain sections (35 μm) were cut on a cryostat and collected as free-floating. The free-floating sections were collected serially and maintained in rostrocaudal order. For each staining target, matched sections were selected at comparable stereotaxic levels across animals, ensuring equivalent anatomical levels were analyzed between groups.

Immunostaining was performed using primary antibodies against glial fibrillary acidic protein (GFAP; Proteintech, #60190-1-Ig, Rosemont, IL; 1:500) to label astrocytes, and ionized calcium-binding adapter molecule 1 (IBA1; Proteintech, #10904-1-AP, 1:500) to label microglia. Sections were incubated with fluorescent secondary antibodies: DyLight 555-conjugated anti-mouse IgG (Thermo Fisher Scientific, SA5-10167, 1:500) for GFAP and DyLight 488-conjugated anti-rabbit IgG (Thermo Fisher Scientific, SA5-10038, 1:500) for IBA1. For striatal terminal-marker analysis, vesicular monoamine transporter 2 (VMAT2; ImmunoStar, #20042, Hudson, WI; 1:500) was also assessed on matched sections using DyLight 488-conjugated anti-rabbit IgG (Thermo Fisher Scientific, SA5-10038, 1:500). Nuclei were counterstained with 4′,6-diamidino-2-phenylindole (DAPI; Sigma-Aldrich, D8417). Stained sections were mounted on glass slides and coverslipped with antifade mounting medium (Vector Labs, H-1000-1, Newark, CA) for imaging.

### Total RNA extraction, cDNA synthesis, and quantitative real-time PCR (RT-qPCR)

Total RNA was isolated from SK-N-SH cells and dissected rat brain tissues using TRI Reagent (Molecular Research Center, TR118, Cincinnati, OH) according to the manufacturer's protocol. Spectrophotometric measurements were used to evaluate RNA concentration and purity at 260 and 280 nm (A260/280 ratio ≥ 1.9 for all samples).

First-strand cDNA synthesis was performed using 0.5 μg of total RNA and random hexamers with M-MLV reverse transcriptase (Thermo Fisher Scientific, #28025013) according to the manufacturer's instructions. Synthesized cDNA was diluted 1:11 in nuclease-free water and stored at − 80 °C until further use.

RT-qPCR was conducted using KAPA SYBR Fast Universal 2 × qPCR Master Mix (Kapa Biosystems, Roche, #07959362001, Basel, Switzerland) in 96-well PCR white microplates (Azenta, Burlington, MA) on a CFX Opus Real-Time PCR System (Bio-Rad, Richmond, CA). Each reaction was performed in technical triplicate, and the three measurements were averaged to generate one expression value per biological sample. No-reverse-transcription controls were included in each run. Gene expression levels were calculated using the 2^−ΔΔCt^ method, with glyceraldehyde-3-phosphate dehydrogenase (GAPDH) used as the internal normalization control. Primer sequences used for qPCR are listed in Additional file 1: Table S1.

### RNA sequencing analysis

#### Data acquisition and preprocessing

Total RNA was extracted using TRIzol Reagent (Thermo Fisher Scientific, #15596018). RNA sequencing was performed by the Greek Genome Center (GGC, Athens, Greece). Library preparation was performed using the NEBNext Ultra II Directional RNA Library Prep Kit for Illumina (New England Biolabs, Ipswich, MA), according to the manufacturer's instructions. Library quality was assessed with an Agilent Bioanalyzer, and quantification was performed using the Qubit High Sensitivity DNA Kit (Thermo Fisher Scientific, Q32851). Sequencing was conducted on the Illumina NovaSeq 6000 platform (Illumina, San Diego, CA), generating single-end 100 bp reads. The resulting FASTQ files were used for downstream analysis.

#### Bioinformatic analysis

All RNA-seq analyses were performed using the MultiOmicsIntegrator workflow (https://github.com/ASAGlab/MOI--An-integrated-solution-for-omics-analyses), a containerized pipeline designed for integrated multi-omics analysis. This workflow leverages Docker to ensure full reproducibility and computational scalability.

#### Quality control and read alignment

Initial quality control of raw reads was performed using FastQC (v0.12.0) and aggregated using MultiQC [17]. Adapter trimming and low-quality base removal were performed using TrimGalore (v0.6.10). Processed reads were aligned in two stages using Salmon (v1.10.2) [18]: (1) Alignment to the *Rattus norvegicus* reference genome (mRatBN7.2) to quantify endogenous gene expression, including *Snca,* and (2) Alignment to the human reference genome (GRCh38) to quantify expression of the human *SNCA* transgene.

#### Differential expression analysis

Differential gene expression analysis was carried out using edgeR (v4.0.16). Genes with low expression were filtered using the filterByExpr function. Data were normalized using the calcNormFactors function with the TMM (trimmed mean of M-values) method. Expression values were modeled using a negative binomial generalized linear model. The experimental design compared three rats treated with scrambled ASO1 and three rats treated with SNCA ASO3.0 across four brain regions. Genes were considered differentially expressed if they met a false discovery rate (FDR) threshold of < 0.05 (Benjamini–Hochberg correction). Only genes with CPM (counts per million) > 0.5 in at least six samples were retained for analysis.

#### Enrichment analysis and system-level interpretation

Differentially expressed genes (DEGs) were interpreted using BioInfoMiner [19], a web-based platform for systemic functional analysis. BioInfoMiner applies graph-theoretic methods and statistical enrichment algorithms to map gene lists onto semantic networks derived from multiple ontologies, including Gene Ontology (GO), Human Phenotype Ontology [20], Mammalian Phenotype Ontology [21], and REACTOME pathways [22]. The output includes a signature matrix that identifies prioritized hub genes and their associated systemic biological processes.

### Preparation of whole protein extracts and immunoblotting

Tissue samples were homogenized in ice-cold high-salt (HS) buffer (50 mM Tris–HCl, pH 7.5, 0.75 M NaCl, 2 mM EDTA, 50 mM NaF) containing 1% Triton X-100 and supplemented with cOmplete™ protease inhibitor cocktail (Roche, #11697498001) and PhosSTOP™ phosphatase inhibitor cocktail (Roche, #4906837001) at a ratio of 4 mL/g tissue. Homogenates were centrifuged at 50,000 × *g* for 60 min at 4 °C. The resulting supernatants (soluble fractions) were collected and stored at − 80 °C.

Pellets were re-homogenized in HS buffer containing 1 M sucrose (3 mL/g) and centrifuged again at 50,000 × *g* for 1 h at 4 °C. The supernatant was discarded, and pellets were resuspended in 4 M urea/2% SDS buffer (3 mL/g), yielding the insoluble fraction. Protein concentration was determined in soluble fractions using the Bradford assay according to the manufacturer's instructions (Bio-Rad, #5000006).

For immunoblotting, equal amounts of protein were mixed with 6 × SDS sample buffer (375 mM Tris–HCl, pH 6.8, 10% SDS, 50% glycerol, 10% β-mercaptoethanol, 0.03% bromophenol blue), boiled at 100 °C for 5 min, separated on 13% SDS-PAGE gels, and transferred onto Protran nitrocellulose membranes (Amersham/Merck, GE10600002, St. Louis, MO). Membranes were blocked in Tris-buffered saline containing 0.1% Tween-20 (TBS-T) and 5% non-fat dry milk for 1 h at room temperature, and incubated overnight at 4 °C with primary antibodies diluted in TBS-T. The primary antibodies included 4B12 (GeneTex, GTX21904, Irvine, CA; 1:1000) and Syn1 (BD Transduction Laboratories, #610787, Franklin Lakes, NJ; 1:1000) for total human α-synuclein (monomeric and higher-molecular-weight), EP1536Y pSer129 SNCA for phosphorylated α-synuclein (Abcam, ab51253, Cambridge, UK; 1:1000), anti-tyrosine hydroxylase (TH) antibody (Proteintech, #25859-1-AP, 1:1000), anti-IBA1 antibody (Proteintech, #10904-1-AP, 1:1000), anti-GFAP antibody (Proteintech, 16825-1-AP, 1:1000), and anti-VMAT2 antibody (Santa Cruz Biotechnology, sc-374079, 1:500). The 4B12 antibody detects human α-synuclein and shows partial cross-reactivity in rat tissue. Syn1 detects both human and rat α-synuclein (Additional file 1: Fig. S2). HRP-conjugated secondary antibodies (Cell Signaling Technologies, #7074 and #7076, Danvers, MA; 1:5000) were applied for 1 h at room temperature. Immunoreactive bands were visualized using Clarity or Clarity Max ECL detection reagents (Bio-Rad, #1705061 and #1705062) and imaged with a Fusion FX6 imaging system (Vilber, Marne-la-Vallée, France). Densitometric quantification was performed in Fiji (NIH), using GAPDH (Proteintech, HRP-60004, 1:15,000) as the internal loading control.

### Multiplex protein analysis

Total protein lysates prepared from dissected frozen brain regions were used for multiplex protein analysis. Protein concentration was determined using the Bradford assay according to the manufacturer's protocol (Bio-Rad). Before analysis, protein samples were diluted to a final concentration of 500 μg/mL using Protavio lysis buffer (Protavio, Athens, Greece).

Bead-based multiplex enzyme-linked immunosorbent assays (ELISA) were performed using color-coded microspheres, each with a unique fluorescent signature corresponding to a specific analyte. Capture antibodies were covalently bound to the microsphere surface. Samples were incubated with the pre-washed bead mix in 96-well microtiter plates. After magnetic separation and washing to remove unbound components, bead-analyte complexes were incubated with biotinylated detection antibodies specific to each target, followed by a final incubation with streptavidin–phycoerythrin (Moss, Inc., Franklin Park, IL).

Data acquisition was performed using the Luminex® FLEXMAP 3D system (Luminex, Austin, TX) with a high photomultiplier tube setting, doublet discriminator gating set to 3000–20,000, and a minimum of 50 bead counts per analyte. Median Fluorescence Intensity (MFI) values were used for statistical analyses. For absolute quantification, net MFI values (blank-subtracted) were fitted using a five-parameter logistic regression model to generate calibration curves. Three custom multiplex panels were employed. (1) Phosphorylation Panel (20-plex, Protavio), targeting selected phosphorylated signaling proteins. Specific proteins and phosphorylation sites are listed in Additional file 1: Table S2. Control lysates from various cell lines stimulated with known activators were used to verify assay performance. (2) Extracellular Protein Panel (13-plex, Protavio), used to profile selected secreted proteins in seven brain regions. Targets are listed in Additional file 1: Table S3. Calibration curves were generated using recombinant proteins. (3) Cytokine Panel (23-plex, Bio-Rad, #12005641), used to quantify cytokines in four brain regions. Target proteins are provided in Additional file 1: Table S4. Absolute concentrations were calculated based on recombinant standards. Custom antibody-coupled beads and multiplex assay panels were technically validated for specificity and reproducibility as previously described [23]. For the custom phosphoprotein panel, measurements were considered reliably above background when the signal-to-noise ratio (SNR), defined as the sample MFI divided by the assay-specific background MFI, was ≥ 3. In the phosphoprotein plots, this threshold is indicated by a horizontal red line. Measurements with an SNR < 3 are shown for completeness but were not interpreted as reliable above-background signals.

### Gene set enrichment analysis (GSEA)

GSEA was conducted using GSEA software version 4.3.3, incorporating either the mouse Biological Process (BP) subset of the GO gene set collection or a custom gene set composed of cell-type markers cataloged in PanglaoDB [24].

### GO analysis

Gene Ontology enrichment analysis of biological processes was conducted using the ClueGO v2.5.10 plug-in [25] within the Cytoscape v3.10.2 platform. The significance threshold was set at *P* < 0.05, with all other parameters maintained at default settings. Network visualization was generated using the yFiles Organic Layout algorithm.

### Physical interaction network of α-synuclein

The physical interaction network of differentially expressed α-synuclein interactors was constructed using the GeneMANIA v3.5.3 plug-in [26] within Cytoscape v3.10.2. Default parameters were used to define physical interactions and build the interaction network.

### Statistical analysis

All experiments included at least 3 biological replicates. Group sizes were *n* = 3 per group for RNA-seq and immunohistochemistry, *n* = 4 per group for HPLC, *n* = 5 per group for cytokine profiling and phospho-kinase assays, and *n* = 3 per group for the auxiliary female HaXha cohort. For qPCR, immunoblotting, and behavioral assays, group sizes varied by assay and are stated in the corresponding figure legends. For all in vivo experiments, each animal constituted one biological replicate and the unit of statistical analysis. Where technical replicates were performed, their values were averaged to generate a single value for each biological sample before statistical analysis. Data are presented as mean ± standard deviation (SD). Data were assessed for normality using the Shapiro–Wilk test. For pairwise comparisons involving matched samples, two-tailed Student’s *t*-tests were used for normally distributed data, and Mann–Whitney U tests for non-parametric data. For human transcript abundance comparison, FDR-corrected two-sided Welch’s *t*-tests (unequal variances) were used. For multi-group comparisons, one-way ANOVA was initially performed, followed by multiple pairwise* t*-tests corrected for multiple comparisons using the Benjamini–Hochberg procedure to control FDR at 5%. For multiple comparisons of animal subjects, two-way ANOVA (study group × brain region) was initially performed, followed by Benjamini–Hochberg FDR-adjusted post hoc comparisons between scrambled ASO1 and SNCA ASO3.0 within each region. Differences were considered statistically significant at *P* < 0.05 for uncorrected pairwise comparisons and at *q* < 0.05 for FDR-adjusted multiple comparisons. All statistical analyses were conducted using GraphPad Prism (version 8.0.0; GraphPad Software, San Diego, CA).

## Results

### *SNCA* 3′UTR architecture and in vitro screening identify ASO3.0 as the lead candidate

**Fig. 1:**
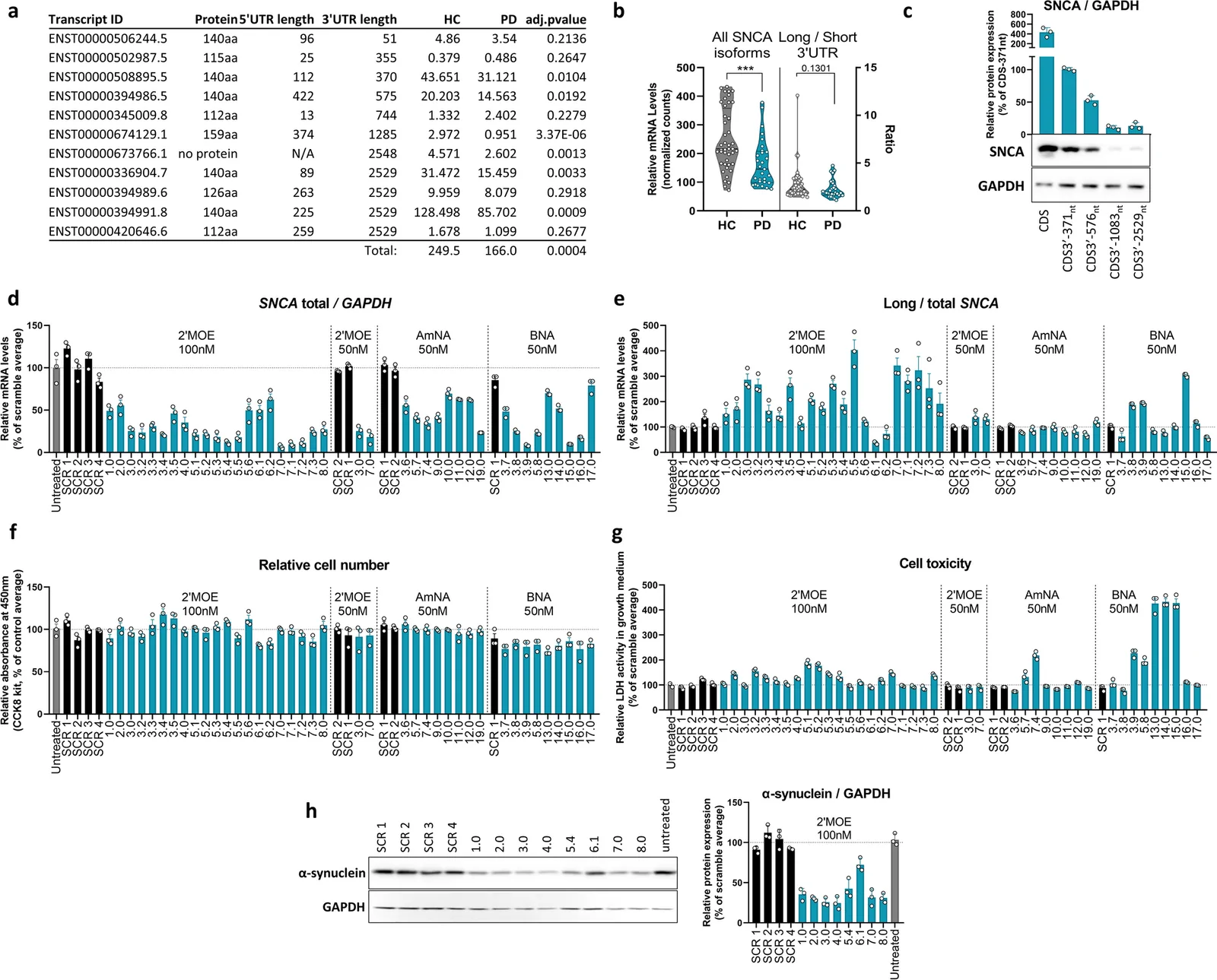
Isoform-specific dysregulation of *SNCA* 3′UTRs in PD and ASO-mediated reduction of translation-efficient transcripts in vitro. **a** Expression levels of individual Ensembl *SNCA* transcript isoforms in prefrontal cortex (Brodmann Area 9) from healthy control (HC) and PD cases.** b** Total *SNCA* mRNA levels and long/short 3′UTR transcript ratios in HC and PD cortex.** c** Alpha-synuclein protein levels in SK-N-SH cells 48 h after transfection with constructs bearing short or long *SNCA* 3′UTRs.** d** Total *SNCA* mRNA levels in SK-N-SH cells 72 h after transfection with 50 or 100 nM 2′-O-methoxyethyl (2′MOE)-, amido-bridged nucleic acid (AmNA)-, or bridged nucleic acid (BNA)-modified ASOs.** e** Long/total 3′UTR transcript ratios after ASO treatment.** f, g** Total cell counts and viability measurements after ASO transfection.** h** Alpha-synuclein protein levels at 72 h after transfection with 100 nM 2′MOE ASOs. Statistical analysis: Differential transcript abundance between healthy controls and patients with PD was assessed using two-sided Welch’s t-tests (unequal variances) for each transcript, with multiple testing adjusted using the Benjamini–Hochberg false discovery rate (FDR) procedure. Total *SNCA* mRNA and long-to-short 3′UTR transcript ratios were tested using Mann–Whitney (***q < 0.001). All other data represent mean ± SD from three biological replicates

Genome-wide association studies have identified a primary PD risk locus spanning the *SNCA* 3′UTR, with several variants positioned near or between annotated polyadenylation sites [3, 4]. To inform isoform-aware ASO design, a previously published prefrontal cortex RNA-seq dataset comprising 29 PD and 44 control cases was reanalyzed to quantify total *SNCA* mRNA and the relative abundance of major Ensembl-annotated 3′UTR isoforms [27]. In the healthy cortex, ENST00000394991.8 (2529-nt 3′UTR) was the predominant isoform and was expressed at approximately threefold higher levels than ENST00000508895.5 (370-nt 3′UTR) (Fig. 1a). Total *SNCA* mRNA was significantly reduced in PD samples (Fig. 1b). Both major isoforms decreased, with a more pronounced reduction in the longest isoform. The median long-to-short 3′UTR ratio declined from 2.3 in controls to 1.9 in PD (*P* = 0.13), suggesting reduced representation of extended 3′UTR transcripts in PD cortex.

To assess the functional consequences of 3′UTR length, SK-N-SH neuroblastoma cells were transfected with *SNCA* constructs carrying either short or long 3′UTRs. Alpha-synuclein protein output declined as 3′UTR length increased (Fig. 1c), in agreement with the inhibitory effect of extended 3′UTRs on translation.

A panel of 38 human-specific gapmer ASOs incorporating 2′-O-methoxyethyl (2′MOE), amido-bridged nucleic acid (AmNA), or bridged nucleic acid (BNA) chemistries with diverse backbone linkages was screened in SK-N-SH cells (Fig. 1d–g). Total *SNCA* was measured using primers targeting the proximal 3′UTR shared by all transcript isoforms, whereas isoform-specific primers were used to quantify transcripts containing the extended 3′UTR. Multiple candidates reduced both total *SNCA* mRNA and long-3′UTR-containing transcripts. The two most potent ASOs were the 2′MOE-modified SNCA ASO3.0 and ASO7.0. At 100 nM, SNCA ASO3.0 reduced total *SNCA* mRNA by 75%, increased the long-to-total 3′UTR ratio to 2.9, and lowered α-synuclein protein by 65% (Fig. 1d–h). ASO7.0 produced a similar qualitative pattern, reducing total *SNCA* mRNA by 93%, increasing the long-to-total ratio to 3.4, and lowering α-synuclein protein by 69%. Cell counts and viability measurements showed no cytotoxicity for either ASO at the concentrations tested (Fig. 1f, g).

SNCA ASO3.0 and ASO7.0 were advanced into preliminary in vivo evaluation. After a single 3 mg i.c.v. dose of ASO7.0, one BAC-h*SNCA* rat was found dead approximately 12 h after injection, one on day 14, and one survived to the planned 45-day endpoint. Necropsy of the earliest animal revealed a small brain clot, consistent with a possible technical complication during cannulation. The day-14 animal was discovered too late for an informative autopsy, and the endpoint animal showed no gross pathology. No overt pre-mortem abnormalities were observed in any of the three ASO7.0-treated animals. Because only one ASO7.0-treated animal survived to the planned endpoint, meaningful endpoint analysis was not possible. In contrast, the full ASO3.0 cohort completed the study without mortality or overt clinical abnormalities. ASO3.0 was therefore selected for detailed characterization.

### SNCA ASO3.0 reduces human *SNCA* transcripts and α-synuclein protein in BAC-h*SNCA* rats

Homozygous BAC-h*SNCA* rats exhibit a two- to three-fold elevation of α-synuclein across major brain regions, quantified with the human-reactive 4B12 antibody (a.a. residues 103–108) and corroborated with Syn1, which recognizes endogenous rat α-synuclein as well (Additional file 1: Fig. S2a, b). This elevation reflects transcription from the human *SNCA* promoter and models the dosage increases observed in *SNCA* multiplication carriers [13].

**Fig. 2:**
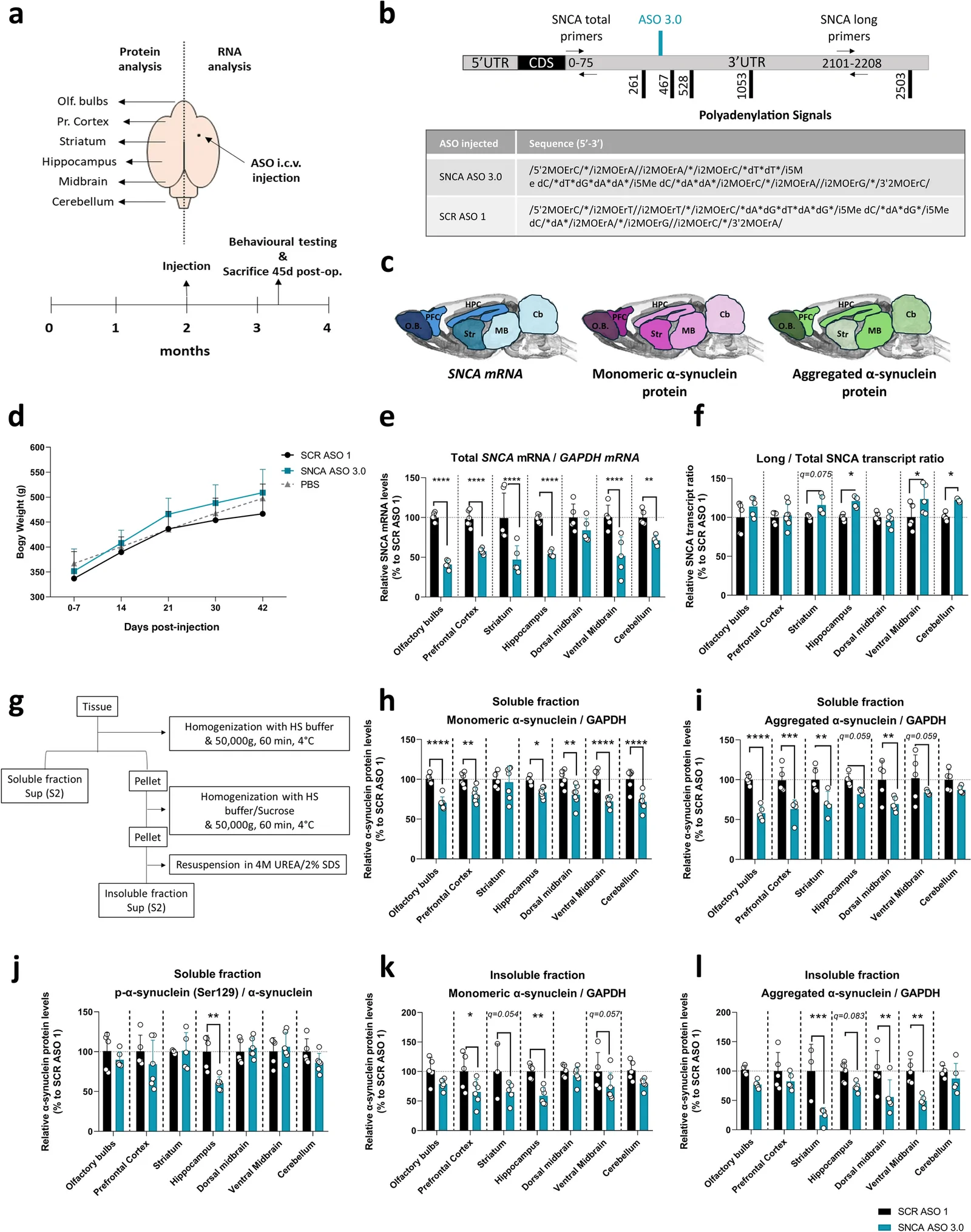
In vivo suppression of *SNCA* transcripts and α-synuclein protein species by SNCA ASO3.0 in BAC-h*SNCA* rats. **a** Experimental timeline for ASO administration and tissue collection.** b** Schematic showing the precise binding site of SNCA ASO3.0 within the SNCA 3′UTR, mapped in relation to established polyadenylation signals, alongside the sequences of SNCA ASO3.0 and the scrambled control ASO1.** c** Anatomical schematic summarizing the regional gradient of *SNCA* suppression after SNCA ASO3.0 treatment, with color intensity representing the magnitude of reduction of *SNCA* mRNA and α-synuclein protein levels relative to scrambled ASO1-treated animals. The sagittal brain template was adapted from the Waxholm Space atlas of the Sprague–Dawley rat brain (v4; Research Resource Identifier (RRID): SCR_017124) [28]. **d** Body weight monitoring six weeks after injection of SNCA ASO3.0 or scrambled ASO1 relative to PBS-injected controls.** e** Regional *SNCA* mRNA levels following SNCA ASO3.0 treatment.** f** Long/total *SNCA* transcript isoform ratio across brain regions.** g** Schematic of protein extraction protocol separating soluble and insoluble fractions. **h–j** Monomeric, aggregated, and serine-129-phosphorylated α-synuclein levels in Triton X-100-soluble fractions. **k, l** Monomeric and detergent-insoluble α-synuclein in SDS-solubilized fractions. Total human α-synuclein was detected with the 4B12 antibody, and pSer129-SNCA with the EP1536Y antibody. Data represent mean ± SD (*n* = 3–7 per group); each point represents one animal (FDR-adjusted *q < 0.05, **q < 0.01, ***q < 0.001). Statistical analysis: Two-way ANOVA (study group × brain region) followed by Benjamini–Hochberg FDR-adjusted post hoc comparisons between scrambled ASO1 and SNCA ASO3.0 within each region

To evaluate human α-synuclein suppression in vivo, 2-month-old rats received a single 3 mg i.c.v. injection of unconjugated SNCA ASO3.0 or a scrambled ASO1 control (Fig. 2). All ASO3.0- and scrambled ASO1-treated animals completed the study without mortality or overt clinical signs. No abnormalities in general activity, posture, gait, tremor, or convulsions were observed during post-anesthetic recovery or within the first 48 h after i.c.v. dosing. Body weight remained stable over the six weeks relative to PBS-injected rats (Fig. 2d). Forty-five days after dosing, hemisected brains were processed for RT-qPCR and immunoblotting.

SNCA ASO3.0 reduced total human *SNCA* mRNA in all regions, with a clear regional gradient summarized schematically in Fig. 2c and quantified in Fig. 2e. The most substantial reductions occurred in the olfactory bulb and striatum (approximately 60%), followed by cortex and hippocampus (approximately 45%), and then cerebellum and midbrain (approximately 30%). The regional gradient matches known patterns of ASO distribution following i.c.v. delivery, where deeper structures typically receive lower exposure [9]. PBS and scrambled-ASO1 groups were indistinguishable across all regions (Additional file 1: Fig. S3a, b). Long and short 3′UTR isoforms were suppressed to a similar extent in most regions. A significant rise in the long/total ratio appeared only in ventral midbrain, hippocampus, and cerebellum (Fig. 2f), indicating greater engagement of the shorter species in these regions. Outside these three regions, the ratio remained unchanged, indicating no detectable shift in 3′UTR isoform representation beyond the overall reduction in human *SNCA*.

Protein measurements were performed on Triton X-100-soluble and SDS-solubilized fractions (Fig. 2g). In the soluble fraction, levels of monomeric, serine-129-phosphorylated, and higher molecular-weight α-synuclein species were measured (Fig. 2h–j and Additional file : Fig. S4). In contrast, only monomeric and detergent-insoluble α-synuclein were detectable in the SDS-solubilized fraction (Fig. 2k, l, and Additional file 1: Fig. S4). Soluble and insoluble total human α-synuclein were detected with the 4B12 antibody, and pSer129-SNCA was quantified with EP1536Y. Protein changes followed the same regional ranking as the mRNA gradient, although the extent of reduction differed between fractions and species, with the clearest and most consistent effects in the soluble fraction. Across regions, soluble higher-molecular-weight α-synuclein species showed stronger reductions than soluble monomers (averaging roughly 45% vs 30% of scrambled ASO1 levels), suggesting that the reduced monomer availability limits higher-order species formation. Changes in the insoluble fraction were more variable across regions and less tightly aligned with the mRNA gradient, likely reflecting the limited dynamic range and region-to-region variability of detergent-insoluble α-synuclein measurements. pSer129-SNCA showed smaller shifts than the total α-synuclein, consistent with the independent regulation of phosphorylation.

These results indicate that SNCA ASO3.0 reduces human *SNCA* transcripts and multiple α-synuclein protein species across regions with a reproducible pharmacodynamic gradient. This gradient provides the context for interpreting the downstream molecular, signaling, and behavioral outcomes.

### SNCA ASO3.0 induces region-specific transcriptomic remodeling in the BAC-h*SNCA* rat brain

Transcriptomic changes induced by SNCA ASO3.0 were assessed using bulk polyadenylated RNA sequencing of the olfactory bulbs, cortex, striatum, and midbrain. DEGs were defined at FDR < 0.05. Region-specific shifts in neuronal and glial signatures were evaluated using GSEA with PanglaoDB-defined cell-type markers. Gene Ontology Biological Process (GO-BP) enrichment was mapped using ClueGO, and BioInfoMiner identified high-contribution nodes within enriched networks. GeneMANIA was used to visualize DEGs participating in curated α-synuclein protein interaction networks. These analyses were used to describe treatment-associated responses under partial *SNCA* lowering rather than to establish experimentally validated pathway maps.

**Fig. 3:**
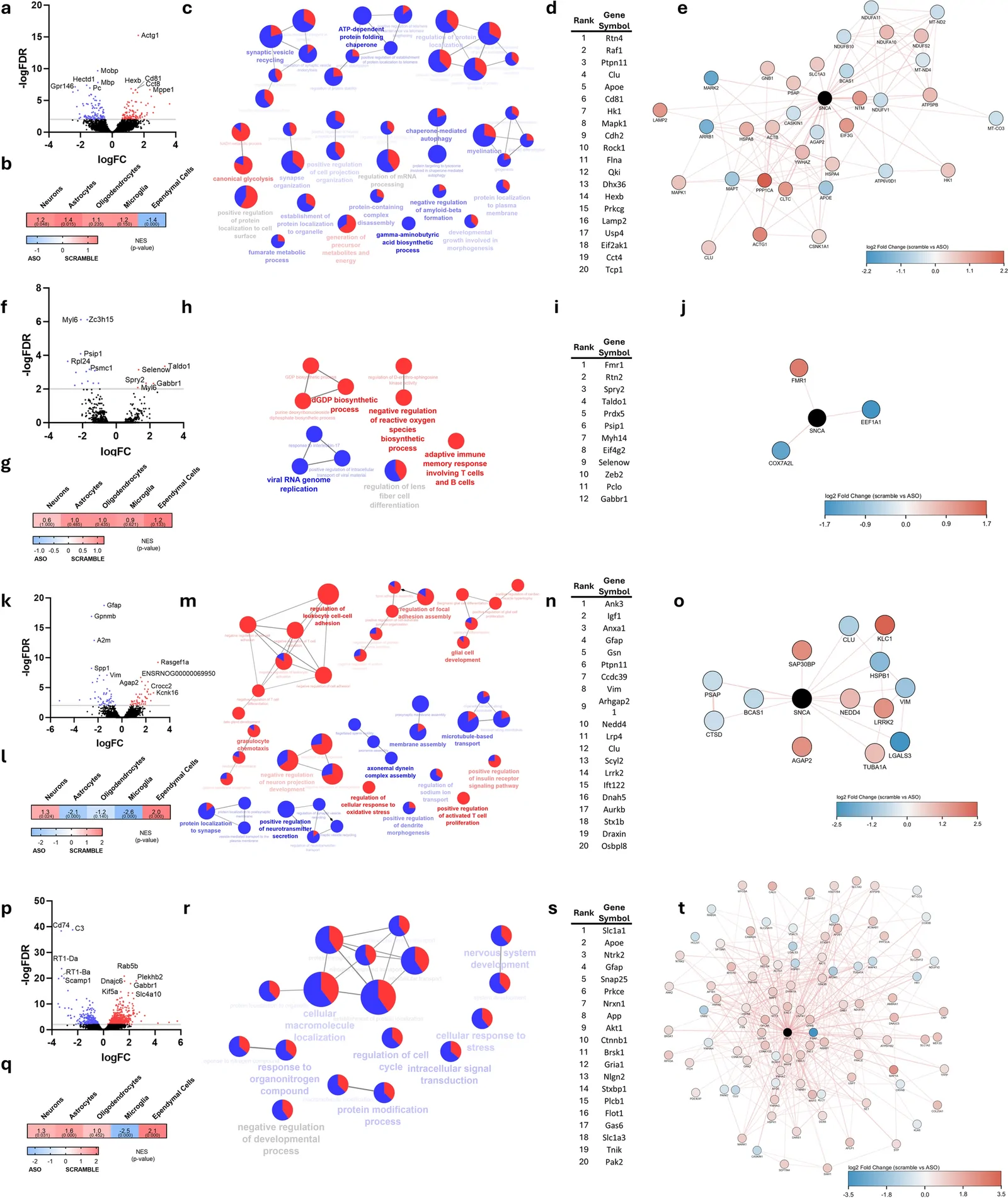
Transcriptomic analysis of olfactory bulbs, cortex, striatum, and midbrain following SNCA ASO3.0 treatment in BAC-h*SNCA* rats. Panels **a-e**, **f-j**, **k–o**, and **p–t** correspond to olfactory bulbs, cortex, striatum, and midbrain, respectively. **a, f, k, p** Volcano plots comparing transcript expression between SNCA ASO3.0 and scrambled ASO1 (FDR < 0.05). **b, g, l, q** GSEA of cell-type-associated gene signatures using PanglaoDB-defined markers. **c, h, m, r** ClueGO enrichment of Gene Ontology Biological Process (GO-BP) terms among differentially expressed genes. **d, i, n, s** BioInfoMiner prioritization of high-contribution genes within enriched networks. **e, j, o, t** GeneMANIA interaction maps showing differentially expressed genes that participate in curated α-synuclein interaction networks. Color coding: blue indicates downregulation and red indicates upregulation

In the olfactory bulb, 279 transcripts were downregulated and 256 upregulated following SNCA ASO3.0 treatment (Fig. 3a; Additional file 2). GSEA revealed coordinated reductions across neuronal and glial gene signatures (Fig. 3b). ClueGO identified positive regulation of protein localization, synaptic vesicle recycling, and myelination as enriched GO-BP terms with predominant downregulation, whereas canonical glycolysis and generation of precursor metabolites and energy were upregulated (Fig. 3c; Additional file 3). BioInfoMiner highlighted *Rtn4, Raf1, Clu, Ptpn11, Rock1*, and *Apoe* as central regulators within the enriched networks (Fig. 3d). GeneMANIA identified 35 DEGs with documented physical interactions with α-synuclein (Fig. 3e). GSEA additionally indicated enrichment of mitochondrial electron transport, xenobiotic metabolism, and synaptic translation pathways in SNCA ASO-treated tissue (Additional files 3 and 4).

Despite strong human α-synuclein suppression, cortical transcriptomic responses were modest, with 11 transcripts downregulated and 30 upregulated (Fig. 3f**; **Additional file 2). GSEA showed minor decreases across neuronal and glial signatures (Fig. 3g). ClueGO identified a limited number of GO-BP terms, including intracellular transport and adaptive immune regulation, with small effect sizes (Fig. 3h**; **Additional file 3). BioInfoMiner prioritized *Fmr1, Rtn2, Spry2, Taldo1,* and *Prdx5* as hub genes within the affected pathways (Fig. 3i). GeneMANIA identified three DEGs that participate in α-synuclein interaction networks (Fig. 3j). The GSEA terms enriched in SNCA ASO-treated cortex were sparse and heterogeneous, and no coherent synaptic, glial, or metabolic cluster emerged.

In the striatum, 106 transcripts were downregulated and 108 upregulated (Fig. 3k; Additional file 2). GSEA showed reduced neuronal signatures and increased astrocytic, oligodendrocytic, and microglial signatures (Fig. 3l). ClueGO identified downregulation of microtubule-based transport, positive regulation of neurotransmitter secretion, and presynaptic membrane assembly, whereas upregulated pathways included negative regulation of neuron projection development, leukocyte cell–cell adhesion, and glial cell development (Fig. 3m; Additional file 3). BioInfoMiner prioritized *Ank3, Igf1, Anxa1, Gfap,* and *Gsn* as influential nodes driving these pathway alterations (Fig. 3n). Thirteen DEGs encoded proteins with documented α-synuclein interactions (Fig. 3o). GSEA additionally identified enrichment of complement cascades, antigen processing, chemokine regulation, and phagocytic control in SNCA ASO-treated tissue (Additional file 4). Western blotting and immunohistochemistry showed no changes in GFAP or IBA1 in the striatum (Fig. S5a–d), arguing against overt reactive gliosis.

In the midbrain, 1120 transcripts were downregulated and 527 upregulated (Fig. 3p**; **Additional file 2). GSEA showed broad repression of neuronal, astrocytic, and oligodendrocytic signatures, accompanied by increased microglial signatures (Fig. 3q). ClueGO identified downregulated pathways involving macromolecule localization, responses to organonitrogen compounds, cell-cycle regulation, and intracellular signal transduction (Fig. 3r; Additional file 3). BioInfoMiner highlighted *Slc1a1, Apoe, Ntrk2, Gfap*, and *Snap25* as key contributors to these transcriptional changes (Fig. 3s). Ninety-seven DEGs mapped to curated α-synuclein protein interaction networks (Fig. 3t). GSEA additionally identified antigen presentation, complement activation, immunoglobulin-related modules, and phagocytic regulation in SNCA ASO-treated midbrain (Additional file 4). As in the striatum, these pathways intersect with glia-synapse signaling and do not correspond to increases in GFAP or IBA1 protein (Additional file 1: Fig. S5a–d).

Collectively, these results indicate that SNCA ASO3.0 is associated with regionally distinct transcriptional responses in the BAC-h*SNCA* rat brain. The olfactory bulbs showed the clearest combination of human *SNCA* reduction and coordinated synaptic and metabolic enrichment. Cortex showed comparatively limited transcriptomic changes despite measurable knockdown. Striatum and midbrain showed larger DEG burdens with glial- and immune-weighted enrichment, but without corresponding increases in GFAP or IBA1 protein.

### SNCA ASO3.0 selectively modulates cytokine and chemokine expression in the BAC-h*SNCA* rat brain

Because oligomeric and aggregated α-synuclein can modulate innate immune signaling in microglia through TLR2, TLR4, and the NLRP3-linked pathways [29–31], selected cytokines were measured as pharmacodynamic readouts following *SNCA* reduction.

**Fig. 4:**
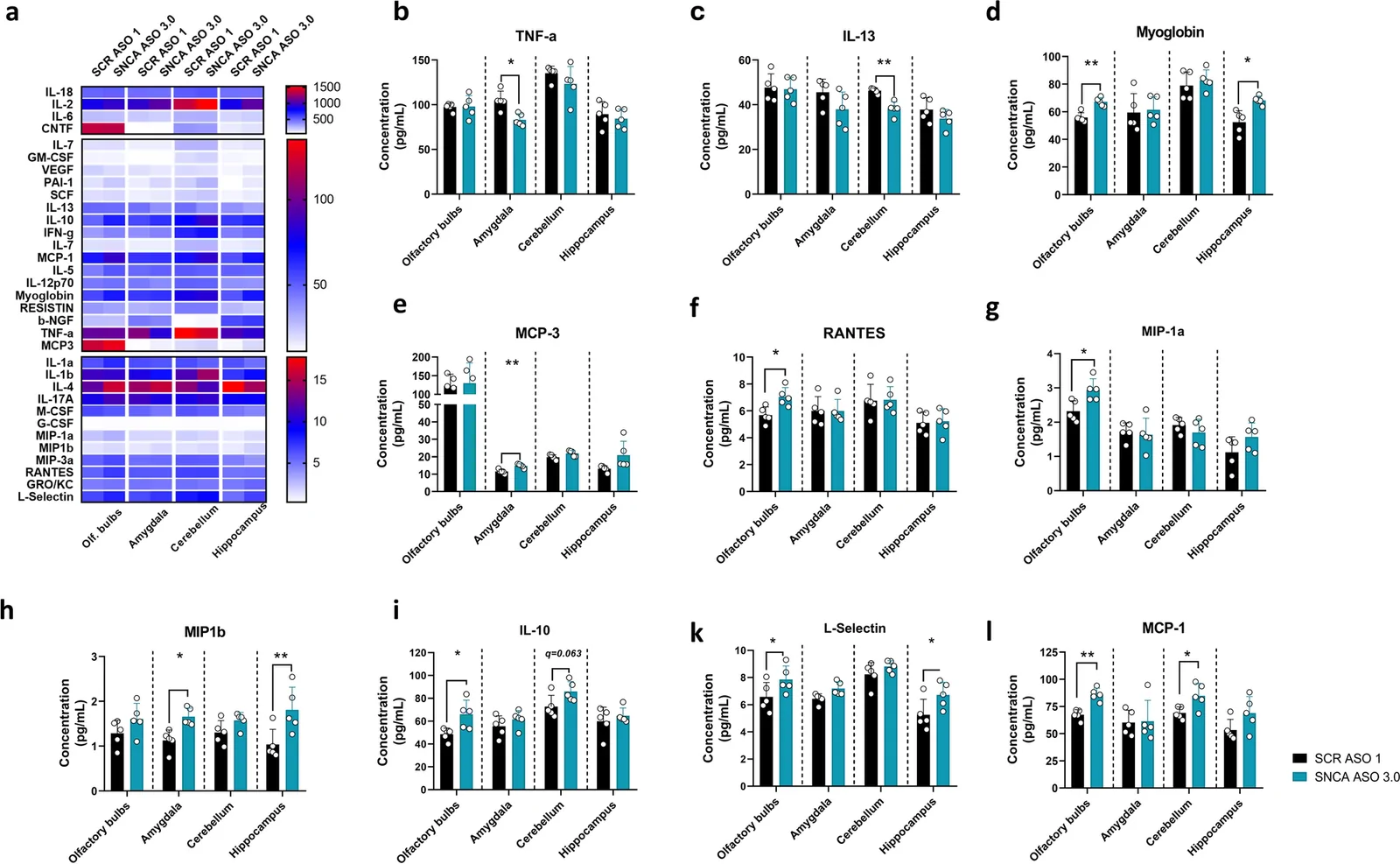
Region-specific modulation of cytokine and chemokine expression by SNCA ASO3.0 in BAC-h*SNCA* rat brain.** a** Heatmap summarizing concentrations of 23 cytokines and related factors in olfactory bulbs, amygdala, cerebellum, and hippocampus 45 days after treatment with SNCA ASO3.0 or scrambled ASO1. **b, c** Cytokines significantly decreased in at least one brain region (TNFα and IL-13). **d–l** Factors significantly increased in at least one region (myoglobin, MCP-3, RANTES, MIP-1a, MIP-1b, IL-10, L-selectin, and MCP-1). The remaining analytes showed no significant changes in any region (see Fig. S6). Data represent mean ± SD (*n* = 5 per group). FDR-adjusted *q < 0.05, **q < 0.01. Statistical analysis: Two-way ANOVA (study group × brain region) followed by Benjamini–Hochberg FDR-adjusted post hoc comparisons between scrambled ASO1 and SNCA ASO3.0 within each region

To examine cytokine changes, a 23-plex bead-based assay was applied to the olfactory bulbs, amygdala, cerebellum, and hippocampus 45 days after SNCA ASO3.0 or scrambled ASO1 administration in homozygous BAC-h*SNCA* rats. Cytokine-based interpretation is therefore restricted to these assayed regions. Most analytes did not differ significantly between treatment groups in any region. A restricted subset showed significant and concordant modulation (Fig. 4a). TNFα and IL-13 concentrations were reduced in at least one region, whereas myoglobin, MCP-3, RANTES, MIP-1β, MIP-1α, L-selectin, IL-10, and MCP-1 were increased (Fig. 4b–l). The remaining cytokines and growth factors, including CNTF, RAGE, β-NGF, PAI-1, SCF, MDC, IL-18, IL-1α/β, IL-2, M-CSF, G-CSF, GM-CSF, IL-4, IL-5, IL-6, IL-7, IL-12p70, IL-17A, IFNγ, GRO/KC, MIP-3α, resistin, and VEGF, remained unchanged across all regions (Additional file 1: Fig. S6).

Together, these data indicate selective, region-restricted modulation of a limited subset of cytokine and chemokine analytes after *SNCA* lowering, with most measured analytes unchanged.

### SNCA ASO3.0 modulates AKT-mTOR signaling without broad effects on stress or inflammatory pathways

PI3K-AKT and mTOR signaling are dysregulated in PD brain tissue. Increased AKT phosphorylation has been reported in human PD brains, where α-synuclein enhances PIK3CA/p110α activity, increasing PI3K-AKT-mTOR output and promoting lipid droplet accumulation [32]. Pathologic α-synuclein also disrupts the TSC1-TSC2 complex, elevating mTORC1 activity and protein synthesis and contributing to neuronal vulnerability [33].

**Fig. 5:**
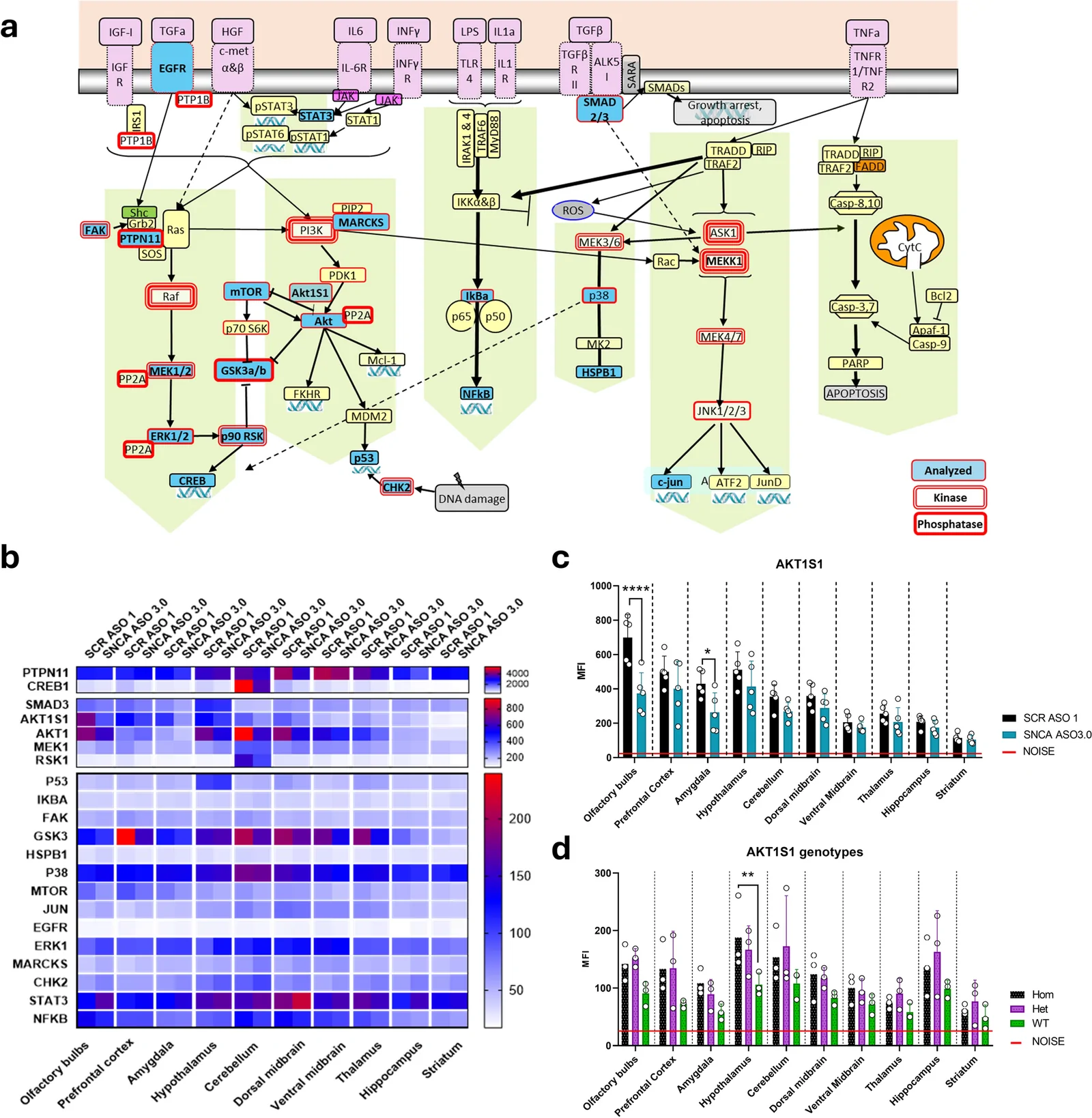
Modulation of AKT-mTOR signaling by SNCA ASO3.0 in BAC-h*SNCA* rat brain. **a** Schematic of the signaling pathways and kinase targets profiled. **b** Heatmap of phosphorylation levels across brain regions 45 days after SNCA ASO3.0 or scrambled ASO1 treatment. **c** Phosphorylation of AKT1S1/PRAS40 after SNCA ASO3.0 treatment, showing the strongest and statistically supported reduction in the olfactory bulbs. **d** Baseline AKT1S1/PRAS40 phosphorylation in untreated BAC-h*SNCA* rats compared with wild-type controls. Where shown, the horizontal red line denotes an SNR of 3, the threshold used to consider the phosphoprotein signal reliably above assay-specific background. Values below this threshold are displayed for completeness but were not interpreted as reliable above-background signals. Data represent mean ± SD (*n* = 3–5 per group). FDR-adjusted **q < 0.01, ****q < 0.0001. Statistical analysis: Two-way ANOVA (study group × brain region) followed by Benjamini–Hochberg FDR-adjusted post hoc comparisons between scrambled ASO1 and SNCA ASO3.0 within each region

To examine how partial *SNCA* suppression affects this signaling axis, phosphorylation states of 21 kinases and downstream targets were measured across 10 brain regions (Fig. 5a). Basal phosphorylation was detected for MARCKS, HSPB1, mTOR, IκBα, FAK, p53, p38, EGFR, CHK2, and NFKB (Fig. 5b, Additional file 1: Fig. S7). Distinct regional profiles were evident, with enriched SMAD3, p53, and EGFR phosphorylation in the hypothalamus and elevated CREB1 and RSK1 in the cerebellum. In contrast, the striatum showed the lowest overall activity.

SNCA ASO3.0 reduced AKT-dependent phosphorylation of AKT1S1/PRAS40, most prominently in the olfactory bulbs (Fig. 5c). This change recapitulated the genotype-associated difference between BAC-h*SNCA* and wild-type rats (Fig. 5d). Because AKT1S1/PRAS40 is an inhibitory component of mTORC1 whose phosphorylation relieves that inhibition, the directionality is consistent with reduced mTORC1-related signaling. Other AKT-mTOR pathway components, including AKT1, PTPN11, mTOR, and GSK3β, showed similar downward trends but did not reach statistical significance (Fig. 5b, Additional file 1: Fig. S7).

In contrast, phosphorylation of IκBα, NFKB, p38, and MEK1 did not change (Fig. 5b, Additional file 1: Fig. S7). The stability of these inflammatory and stress-related readouts aligns with the unchanged GFAP and IBA1 protein levels, and the modest glial transcriptional shifts in striatum and midbrain. These features indicate that kinase adjustments after α-synuclein reduction occur without overt inflammatory activation. The pattern suggests that partial α-synuclein suppression attenuates the PI3K-AKT-mTOR signaling while leaving broader stress-responsive pathways stable.

### SNCA ASO3.0 does not alter the striatal neurotransmitter levels in young BAC-h*SNCA* rats

Dopaminergic vulnerability develops progressively in both PD patients and BAC-h*SNCA* rats. Absolute striatal DA can remain stable during early phases despite substantial molecular and synaptic stress. In BAC-h*SNCA* rats, reductions in striatal DA become prominent later in life, with clearer degeneration reported around 12–18 months [13]. At 3.5 months, the model is therefore expected to exhibit preserved DA content alongside early molecular alterations, corresponding to an early symptomatic stage and a closer alignment with an early-intervention setting rather than a late-stage rescue paradigm.

**Fig. 6:**
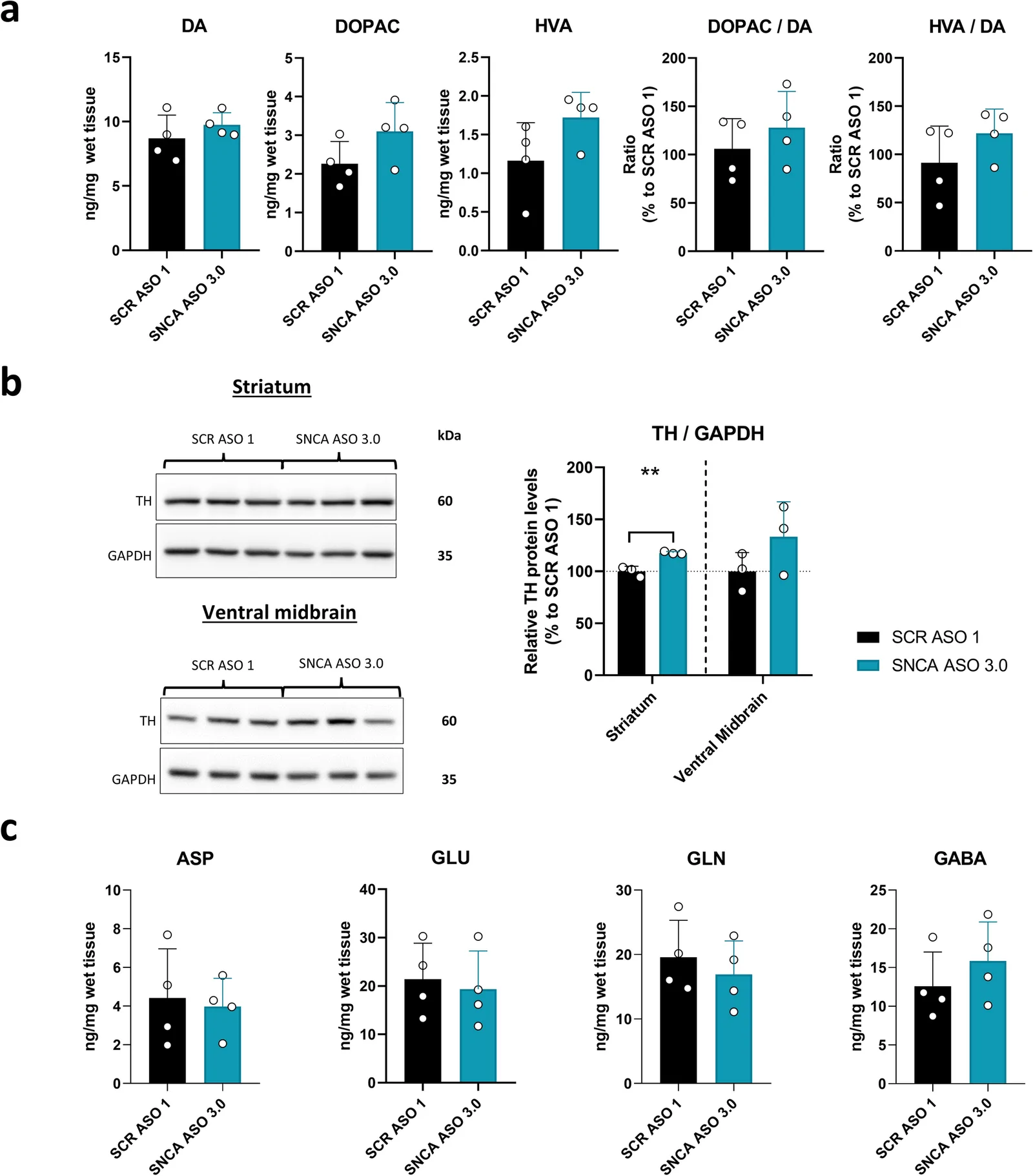
Effects of SNCA ASO3.0 on striatal neurotransmission in BAC-h*SNCA* rats.** a** Striatal dopamine (DA), dihydroxyphenylacetic acid (DOPAC), homovanillic acid (HVA), and turnover ratios. **b** Tyrosine hydroxylase (TH) protein levels in the striatum and ventral midbrain. **c** Striatal concentrations of aspartate (ASP), glutamate (GLU), glutamine (GLN), and γ-aminobutyric acid (GABA). Data represent mean ± SD (*n* = 3–4 per group) (**P < 0.01). Statistical analysis: Student’s *t*-test between scrambled ASO1 and SNCA ASO3.0 within each region

To determine whether partial *SNCA* suppression influences early dopaminergic dynamics, striatal DA and its metabolites DOPAC and HVA were quantified. Absolute concentrations of DA, DOPAC, and HVA were not different between groups (Fig. 6a), consistent with this early time point. The DOPAC/DA and HVA/DA ratios showed a mild upward trend. These ratios are typically lower in patients with PD than in healthy controls [34].

TH protein increased by 14% in the striatum after SNCA ASO3.0 treatment (Fig. 6b), with no significant changes in the ventral midbrain. This striatal specificity is consistent with the local adaptation of dopaminergic terminals, which occurs without detectable changes in overall dopamine levels [35]. Striatal VMAT2 was also assessed by immunoblotting and immunohistochemistry and did not differ significantly between rats treated with scrambled ASO1 and SNCA ASO3.0 (Additional file 1: Fig. S5e, f), arguing against a broad parallel change in presynaptic dopaminergic terminal markers at this stage.

Additional neurotransmitters associated with corticostriatal integration were examined. Alterations in aspartate, glutamate, glutamine, and γ-aminobutyric acid have been documented in more advanced denervation models [36–38]. In the present early-stage cohort, SNCA ASO did not significantly affect these measures. Modest, non-significant shifts toward lower aspartate, glutamate, glutamine, and a slightly higher γ-aminobutyric acid, were detected (Fig. 6c).

Overall, the neurotransmitter profile remained stable at this age. The combination of unchanged dopamine content, a regional increase in TH, and stable amino acid transmitters indicates that partial *SNCA* lowering produces early molecular adjustments in dopaminergic terminals without altering bulk neurotransmitter levels in young BAC-h*SNCA* rats.

### SNCA ASO3.0 treatment enhances locomotor activity and olfactory-driven exploration in BAC-h*SNCA* rats

The BAC-h*SNCA* rat model develops progressive behavioral deficits affecting locomotion, exploration, and olfactory discrimination. These features emerge despite relatively preserved dopamine content at young ages and reflect early functional changes in the context of elevated human α-synuclein levels rather than advanced nigrostriatal degeneration [13].

**Fig. 7:**
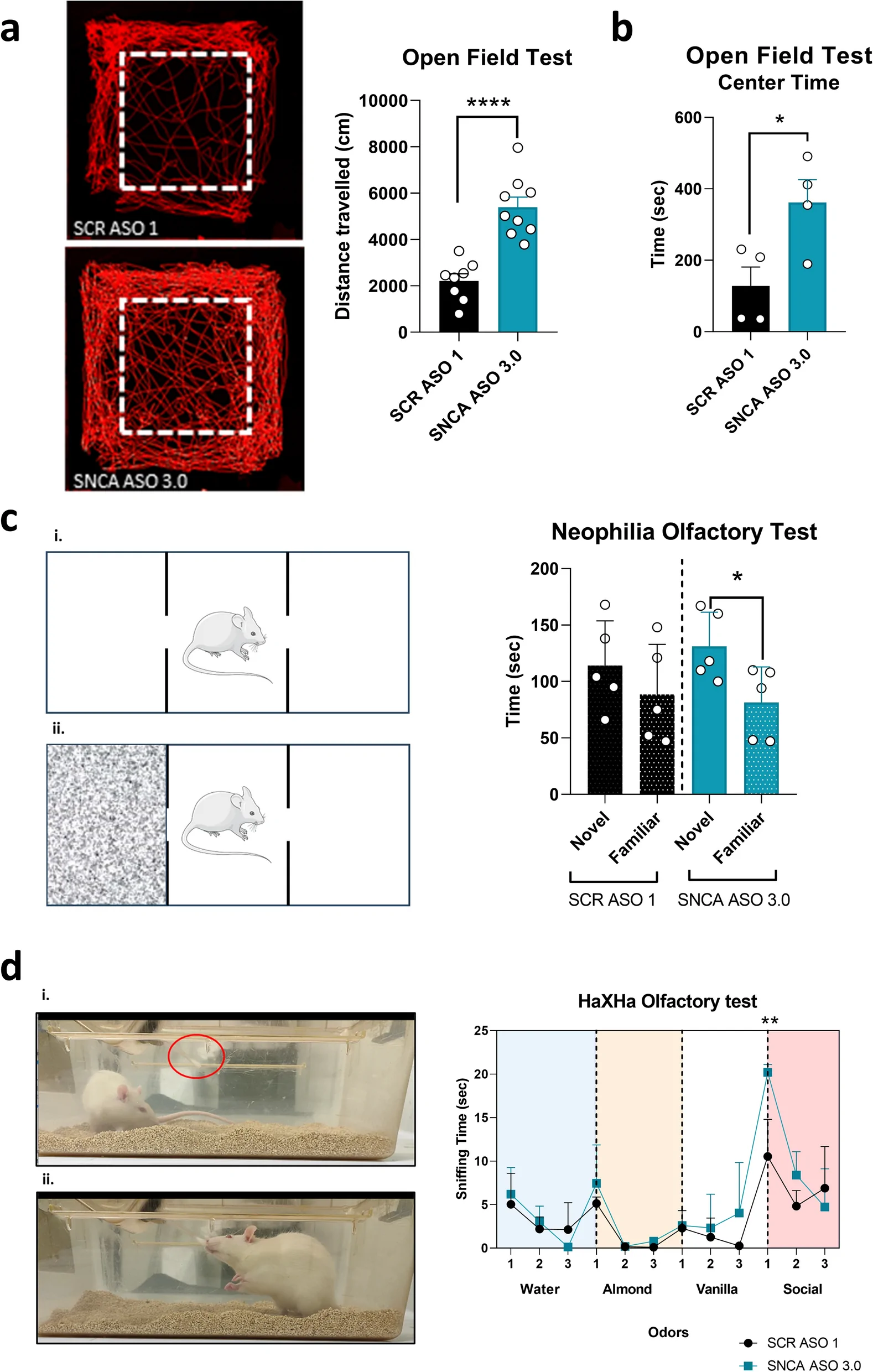
Behavioral outcomes following SNCA ASO3.0 treatment in BAC-h*SNCA* rats. **a** Locomotor activity assessed as total distance traveled in an open field. **b** Time spent in the center of the open field arena. **c** Time spent in the unfamiliar compartment of a three-compartment exploration apparatus (neophilia test). **d** HaXha responses to sequential presentations of almond, vanilla, and social odorants. Data represent mean ± SD; each point represents one animal. For panels **a–c**, male BAC-h*SNCA* rats were used, with panel-specific sample sizes indicated in the graph (*n* = 4**–**9 per group). For panel **d**, a separate female BAC-h*SNCA* cohort was used (*n* = 3 per group). No technical replicate was analyzed as an independent biological observation (*P < 0.05, ***P < 0.001). Statistical analysis: Student’s *t*-test between scrambled ASO1 and SNCA ASO3.0. Some graphical elements in panel **c** were adapted from Servier Medical Art under a Creative Commons Attribution 3.0 Unported License

To evaluate whether partial SNCA suppression affects these early behaviors, open-field testing was performed. The SNCA ASO3.0-treated rats showed a two-fold increase in the total distance traveled compared with scrambled controls (Fig. 7a). The time spent in the center zone increased approximately three folds (Fig. 7b). Greater center occupancy in this model reflects enhanced exploratory drive and reduced anxiety.

Olfactory function was examined because hyposmia is a prominent early feature of PD, and BAC-h*SNCA* rats exhibit impaired odor discrimination by three months of age [13]. In a three-compartment assay containing familiar and novel sawdust, the SNCA ASO3.0-treated animals spent significantly more time investigating the compartment with novel odor cues (Fig. 7c). Increased engagement with the novel compartment indicates enhanced neophilic exploration and aligns with the robust SNCA knockdown and pathway adjustments detected in the olfactory bulbs.

To assess odor discrimination more directly, the HaXha paradigm was applied using almond, vanilla, and a social odorant. Treated rats exhibited steeper habituation to repeated presentations of each odor and showed enhanced cross-habituation to the social odorant (Fig. 7d). Cross-habituation to almond and vanilla was comparable between groups.

Overall, these results show that moderate *SNCA* knockdown enhances locomotor activity, increases exploratory drive, and improves specific components of olfactory discrimination in young BAC-h*SNCA* rats. The behavioral profile aligns with the pattern of early molecular adaptations detected across the striatum, olfactory bulb, and associated signaling pathways, at a stage when bulk neurotransmitter levels remain stable.

## Discussion

In this study, we show that partial suppression of human *SNCA* in BAC-h*SNCA* rats yields anatomically distinct molecular and behavioral outcomes in a model with progressive dopaminergic and olfactory phenotypes [13]. A single i.c.v. dose of SNCA ASO3.0 lowered human *SNCA* transcripts and α-synuclein protein species across multiple regions, altered selected kinase and cytokine readouts, shifted transcriptomic signatures, and improved locomotor and olfactory-driven behaviors. These effects were quantified at a stage when human α-synuclein is elevated and subtle behavioral alterations are present, while striatal dopamine content is preserved.

Previous studies with *SNCA*-directed ASOs established that partial α-synuclein suppression is feasible and can attenuate pathology in mouse and non-human primate models driven by cDNA overexpression or fibril exposure [7, 8]. The present work adds three elements to that foundation. First, the humanized BAC-h*SNCA* rats carry the intact human *SNCA* locus, including the promoter and the full 3′UTR architecture, so ASO engagement occurs across the complete set of annotated human transcripts rather than a single transgene. This configuration allows isoform-aware design and assessment of 3′UTR-dependent effects in a genomic setting that more closely resembles the human locus. Second, pharmacodynamic readouts are resolved across anatomically distinct brain regions within the same cohort, integrating *SNCA* mRNA and α-synuclein protein, kinase signaling, cytokines, bulk transcriptomics, and behavior to map how partial, spatially heterogeneous *SNCA* suppression translates into region-specific circuit and glial adaptations. Third, the use of a young cohort samples an early symptomatic window, before overt nigrostriatal dopamine loss, which is closer to the anticipated intervention stage for α-synuclein-lowering strategies. Together, these features position the study as a bridge between earlier feasibility work in overexpression models and the regionally heterogeneous target engagement expected in clinical α-synuclein-lowering trials.

SNCA ASO3.0 produced a reproducible gradient of *SNCA* suppression, with higher knockdown in the olfactory bulb and cortex and lower knockdown in the cerebellum and midbrain. This pattern parallels quantitative maps of RNase H ASO distribution and activity across rodent and non-human primate CNS after central administration, which show higher exposure in superficial regions and more modest effects in deeper nuclei [9]. Single-nucleus RNA-seq studies further indicate that ASO activity is heterogeneous at the cellular level, with neurons exhibiting deeper knockdown and longer-lasting effects than regional averages would suggest [39]. Although the SNCA ASO3.0 design was informed by the structural diversity of human SNCA 3′UTR isoforms and by evidence that long and short *SNCA* 3′UTR transcripts are differentially represented in PD [40], in vivo long/total 3′UTR ratios changed only modestly and in a region-restricted manner, and several regions showed proportional suppression of long and short transcripts. This pattern may reflect differences in *SNCA* alternative polyadenylation control between the human transcript architecture and the rat cellular environment. In this setting, SNCA ASO3.0 primarily acts by lowering total human *SNCA* in vivo on a human 3′UTR background, while producing more pronounced isoform reweighting in human-derived cellular systems.

Region-resolved RNA-seq indicated that SNCA ASO3.0 treatment did not impose a single transcriptional program but instead elicited distinct responses in the olfactory bulbs, cortex, striatum, and midbrain. Olfactory bulbs and striatum, which combine a high human *SNCA* burden with relatively stronger ASO engagement, showed coordinated adjustments in synaptic, structural, and metabolic pathways. In contrast, the cortex remained comparatively stable, whereas the midbrain carried the largest DEG burden with enrichment of glial- and immune-related modules. Independent transcriptomic profiling in untreated BAC-h*SNCA* rats likewise identified region-specific and age-dependent expression changes rather than a uniform DEG pattern [14]. In the present study, the pronounced midbrain signal was captured in bulk tissue containing mixed neuronal and glial populations and is therefore best interpreted as a composite regional response that may reflect intrinsic vulnerability, regional *SNCA* burden, cellular heterogeneity, and uneven target engagement rather than a single defined pathway state. These bulk RNA-seq data therefore reflect region-averaged responses across heterogeneous cell types, and single-nucleus RNA-seq would be needed to resolve cell-specific contributions.

BioInfoMiner prioritization identified high-contribution hubs within these networks. In the olfactory bulb, decreased *Rtn4*, *Rock1*, and *Clusterin* point to processes linked to neurite constraint and extracellular α-synuclein handling [41–43]. In the cortex, increased *Fmr1* and *Prdx5* alongside reduced *Spry2* suggest altered translational control, ERK-linked regulation, and redox buffering [44–46]. In the striatum, increased *Igf1* and *Anxa1*, together with reduced *Ank3* and *Lrrk2*, are consistent with neurotrophic, resolution-associated, and PD-linked signaling changes [47–54]. In the midbrain, downregulation of *Slc1a1*, *Snap25*, *Stxbp1*, and *Nrxn1*, together with increased *ApoE* and *Gas6*, is consistent with combined presynaptic and glial responses [55, 56]. GeneMANIA networks revealed that a substantial subset of striatal and midbrain DEGs encode proteins with documented physical or functional interactions with α-synuclein or with PD-linked proteins such as LRRK2, APP, and PARK7. Considered together, these examples converge on recurrent themes of presynaptic regulation, structural plasticity, metabolic adaptation, and glial-associated responses.

The olfactory bulb provided the clearest example of aligned target engagement and functional change: SNCA ASO3.0 reduced human *SNCA* mRNA, decreased α-synuclein protein species, and improved odor-driven exploratory behavior. The striatum showed strong human *SNCA* transcript reduction, but protein changes were more species- and fraction-dependent. The accompanying increase in TH, together with unchanged dopamine and VMAT2 levels, and increased locomotor activity, point to early presynaptic readjustment rather than major remodeling of dopaminergic terminal density or structural neuroprotection. This interpretation is consistent with germline *Snca* deletion and transgenic overexpression studies showing that α-synuclein regulates dopaminergic transmission in an activity-dependent manner: *Snca* loss increases paired-stimulus dopamine release, whereas elevated α-synuclein impairs synaptic vesicle reclustering after endocytosis and alters burst-evoked dopamine release in an interval-dependent manner [35, 57, 58]. Modest α-synuclein reduction in dopaminergic terminals could therefore alter release dynamics without necessarily changing the bulk tissue dopamine levels at this age, consistent with the present neurochemical and behavioral findings. The glial- and immune-weighted transcriptomic changes in the striatum and midbrain are more likely to reflect secondary responses to altered neuronal and synaptic activity than to be primary drivers of the behavioral phenotype.

SNCA ASO3.0 produced selective cytokine changes across the assayed regions rather than a broad shift across the full analyte panel. TNFα and IL-13 were reduced in at least one region, whereas IL-10, MCP-1, MCP-3, MIP-1α, MIP-1β, RANTES, L-selectin, and myoglobin were increased selectively across regions. Together, these changes are compatible with a shift toward reduced pro-inflammatory tone after partial α-synuclein lowering. By contrast, IL-1α, IL-1β, and IL-18 remained unchanged under the present conditions, consistent with either selective cytokine modulation by α-synuclein lowering or with the absence of elevation of these inflammasome-linked mediators by the underlying α-synuclein-overexpression state in the assayed regions at this stage.

Kinase profiling identified a narrower intracellular signaling response. The most robust and statistically supported effect was reduced AKT1S1/PRAS40 phosphorylation, particularly in the olfactory bulbs, whereas indices of NFκB and MAPK pathway activation (IκBα, NFKB, p38, MEK1) did not change. Given evidence that excess α-synuclein can amplify PI3K-AKT-mTOR activity and lipid and lysosomal-associated stress [32], this pattern suggests that partial α-synuclein lowering attenuates the AKT-mTOR pathway load without engaging broad inflammatory or stress kinase responses.

From a therapeutic perspective, the tolerability and pharmacodynamic profile of SNCA ASO3.0 are broadly consistent with central nervous system (CNS)-directed antisense literature. Phosphorothioate-modified ASOs with 2′ sugar substitutions show efficient distribution from cerebrospinal fluid (CSF) into the parenchyma, long tissue half-lives, and predictable RNase H-mediated target engagement [59]. CNS-wide studies in rodents and non-human primates report durable knockdown across neurons and glia with broad regional activity after central delivery [9, 39, 60]. Chronic and class-effect mouse studies of 2′-MOE ASOs also support generally consistent tolerability across development candidates [61, 62]. In the present study, all ASO3.0-treated animals completed the experiment without mortality or overt clinical abnormalities, and no changes in GFAP, IBA1, or inflammatory kinase readouts (IκBα, NFKB, p38) were detected across brain regions. A separate LC–MS/MS biodistribution study of the same ASO3.0 molecule under the same dosing conditions detected the ASO across multiple brain regions, including cortex, cerebellum, midbrain, and hypothalamus, 45 days after dosing [63], in line with these pharmacokinetic principles.

The study also offers translational insights. Partial *SNCA* suppression produced measurable functional benefits, consistent with reports that RNAi-mediated α-synuclein lowering by 30%–50% is well tolerated in mice, rats, and non-human primates [64–66]. Clinical trials with α-synuclein-lowering antibodies have also reported no serious adverse events [67–69]. Region-specific pharmacodynamics support the feasibility of intrathecal administration strategies that typically produce spatial gradients in humans. These features suggest that early intervention with partial, regionally heterogeneous α-synuclein suppression may be sufficient to modify vulnerable circuits before sustained dopaminergic loss, a level of target reduction that is realistic for human intrathecal dosing.

Several limitations constrain interpretation. All readouts were collected at a single 45-day interval, so the temporal evolution of AKT-mTOR signaling, cytokine modulation, striatal TH changes, and behavioral effects cannot be resolved. The cohort size was modest for several assays, limiting confidence in region-restricted trends. Knockdown was partial and regionally heterogeneous, reflecting clinical reality but complicating causal interpretation of downstream regional effects. RNA-seq was performed on bulk tissue without a wild-type transcriptomic comparator, so cell-type-specific responses, formal reversal toward a wild-type state, and the relative contribution of regional exposure differences versus on-target *SNCA*-dependent effects cannot be directly assigned. Aggregate-selective α-synuclein species were assessed with detergent solubility rather than conformation-specific antibodies. Electrophysiological consequences of altered presynaptic function were not tested. Fine-scale ASO penetration, concentration gradients, and cell-type-selective uptake were not resolved directly. Although no mortality or overt clinical abnormalities were observed in the ASO3.0 cohort, subtle off-target or low-grade toxic effects cannot be fully excluded from the present dataset. A lower 1.5 mg dose produced qualitatively similar but smaller pharmacodynamic effects, consistent with dose dependence, but only one *SNCA*-targeting ASO sequence was characterized in detail, so the extent to which downstream responses generalize across distinct *SNCA*-targeting ASOs remains to be determined.

Despite these constraints, the integrated dataset clarifies how partial, regionally heterogeneous suppression of human *SNCA* maps to anatomically distinct molecular signatures and measurable behavioral changes in a humanized BAC-h*SNCA* rat. The results argue against a uniform brain-wide response and instead support a model in which the magnitude and the regional context of target engagement shape distinct transcriptional, signaling, and functional adaptations.

## Conclusions

A single intracerebroventricular administration of a human *SNCA* 3′UTR-targeting ASO produced a reproducible, regionally graded reduction of human *SNCA* expression in BAC-h*SNCA* rats. Region-resolved profiling indicates that partial *SNCA* lowering is accompanied by anatomically distinct transcriptomic and signaling changes, together with selective modulation of cytokine/chemokine readouts, rather than a uniform global response. These molecular effects co-occurred with increased locomotor activity and higher performance on selected olfactory-driven measures at an age when striatal dopamine content remained preserved. Collectively, these data define early, region-specific consequences of partial in vivo* SNCA* lowering and provide a pharmacodynamic framework for interpreting regionally heterogeneous ASO target engagement in the context of synucleinopathy therapeutics.

## Supplementary Information

Additional file 1. **Figure S1**. Representative visualization of intracerebroventricular injectate spread using Fast Green FCF. **Figure S2**. Western Blots of α-synuclein protein expression across brain regions in WT and BAC-h*SNCA* rats. **Figure S3**. Transcript-level comparison of PBS and scrambled ASO1.** Figure S4**. Western blots of α-synuclein protein expression in BAC-h*SNCA* rats following ASO3.0 treatment. **Figure S5**. SNCA ASO3.0 treatment does not alter GFAP, IBA1, or VMAT2 readouts in BAC-h*SNCA* rats. **Figure S6**. Cytokines unaffected by SNCA ASO3.0 treatment in homozygous BAC-h*SNCA* rats. **Figure S7**. Kinases and kinase targets with no phosphorylation changes after ASO3.0 treatment. **Table S1**. Primer sequences used for cloning and RT-qPCR. **Table S2**. Target proteins and phosphorylation sites included in the custom-developed 20-plex phosphoprotein assay panel. **Table S3**. Target proteins included in the custom-developed 13-plex extracellular protein assay panel. **Table S4**. Target proteins included in the Bio-Plex Pro™ Rat Cytokine 23-Plex assay panel

Additional file 2. **Data S1**. Differentially expressed transcripts following SNCA ASO3.0 delivery in homozygous BAC-h*SNCA* rats

Additional file 3. **Data S2**. Overrepresented Gene Ontology biological processes and corresponding differentially expressed genes identified by ClueGO analysis

Additional file 4. **Data S3**. Results of Gene Set Enrichment Analysis on the mouse Gene Ontology - Biological Process gene set collection Additional file 5. Uncropped Western blot images

Additional file 5. Uncropped Western blot images

## Data Availability

The RNA-seq data have been deposited in the Gene Expression Omnibus (GEO) repository with the dataset identifier GSE293775. All other data are provided in the article and Supplementary Information.

## Abbreviations

2′MOE: 2′-O-methoxyethyl
3′UTR: 3′ Untranslated region
ASO: Antisense oligonucleotide
BAC: Bacterial artificial chromosome
BNA: Bridged nucleic acid
BP: Biological process
DA: Dopamine
DAPI: 4′,6-Diamidino-2-phenylindole
DEG: Differentially expressed genes
DOPAC: Dihydroxyphenylacetic acid
ELISA: Enzyme-linked immunosorbent assay
FDR: False discovery rate
GFAP: Glial fibrillary acidic protein
GSEA: Gene set enrichment analysis
GO-BP: Gene ontology-biological process
GPA: Gene prioritization analysis
HaXha: Habituation/Cross-Habituation
HPLC: High-performance liquid chromatography
HS: High-salt
HVA: Homovanillic acid
IBA1: Ionized calcium-binding adapter molecule
MFI: Median fluorescence intensity
OPA: O-phthaldialdehyde
PD: Parkinson’s disease
RT-qPCR: Reverse transcription-quantitative real-time PCR
TH: Tyrosine hydroxylase
VMAT2: Vesicular monoamine transporter 2

## References

[1] Sharma M, Burre J. alpha-Synuclein in synaptic function and dysfunction. Trends Neurosci. 2023;46(2):153–66. 36567199 10.1016/j.tins.2022.11.007 PMC9877183

[2] Singleton AB, Farrer M, Johnson J, Singleton A, Hague S, Kachergus J, et al. alpha-Synuclein locus triplication causes Parkinson’s disease. Science. 2003;302(5646):841. 14593171 10.1126/science.1090278

[3] Simon-Sanchez J, Schulte C, Bras JM, Sharma M, Gibbs JR, Berg D, et al. Genome-wide association study reveals genetic risk underlying Parkinson’s disease. Nat Genet. 2009;41(12):1308–12. 19915575 10.1038/ng.487 PMC2787725

[4] Nalls MA, Blauwendraat C, Vallerga CL, Heilbron K, Bandres-Ciga S, Chang D, et al. Identification of novel risk loci, causal insights, and heritable risk for Parkinson’s disease: a meta-analysis of genome-wide association studies. Lancet Neurol. 2019;18(12):1091–102. 31701892 10.1016/S1474-4422(19)30320-5 PMC8422160

[5] Soldner F, Stelzer Y, Shivalila CS, Abraham BJ, Latourelle JC, Barrasa MI, et al. Parkinson-associated risk variant in distal enhancer of alpha-synuclein modulates target gene expression. Nature. 2016;533(7601):95–9. 27096366 10.1038/nature17939 PMC5042324

[6] Fujiwara H, Hasegawa M, Dohmae N, Kawashima A, Masliah E, Goldberg MS, et al. alpha-Synuclein is phosphorylated in synucleinopathy lesions. Nat Cell Biol. 2002;4(2):160–4. 11813001 10.1038/ncb748

[7] Cole T, Paumier K, Zhao H, Weihofen A, Kordasiewicz H, Swayze E. Snca targeted antisense oligonucleotides mediate progression of pathological deposition in alpha synuclein rodent transmission models of Parkinson’s disease. Neurology. 2016;86(16):6–239.

[8] Uehara T, Choong CJ, Nakamori M, Hayakawa H, Nishiyama K, Kasahara Y, et al. Amido-bridged nucleic acid (AmNA)-modified antisense oligonucleotides targeting alpha-synuclein as a novel therapy for Parkinson’s disease. Sci Rep. 2019;9(1):7567. 31110191 10.1038/s41598-019-43772-9 PMC6527855

[9] Jafar-Nejad P, Powers B, Soriano A, Zhao H, Norris DA, Matson J, et al. The atlas of RNase H antisense oligonucleotide distribution and activity in the CNS of rodents and non-human primates following central administration. Nucleic Acids Res. 2021;49(2):657–73. 33367834 10.1093/nar/gkaa1235 PMC7826274

[10] Je G, Guhathakurta S, Yun SP, Ko HS, Kim YS. A novel extended form of alpha-synuclein 3’UTR in the human brain. Mol Brain. 2018;11(1):29. 29801501 10.1186/s13041-018-0371-x PMC5970512

[11] Marchese D, Botta-Orfila T, Cirillo D, Rodriguez JA, Livi CM, Fernandez-Santiago R, et al. Discovering the 3’ UTR-mediated regulation of alpha-synuclein. Nucleic Acids Res. 2017;45(22):12888–903. 29149290 10.1093/nar/gkx1048 PMC5728410

[12] Rhinn H, Qiang L, Yamashita T, Rhee D, Zolin A, Vanti W, et al. Alternative alpha-synuclein transcript usage as a convergent mechanism in Parkinson’s disease pathology. Nat Commun. 2012;3:1084. 23011138 10.1038/ncomms2032 PMC3660047

[13] Nuber S, Harmuth F, Kohl Z, Adame A, Trejo M, Schonig K, et al. A progressive dopaminergic phenotype associated with neurotoxic conversion of alpha-synuclein in BAC-transgenic rats. Brain J Neurol. 2013;136(Pt 2):412–32. 10.1093/brain/aws358 PMC3572936 23413261

[14] Hoof V, Casadei N, Riess O, Schulze-Hentrich J, Hentrich T. Brain region-specific and systemic transcriptomic alterations in a human alpha-synuclein overexpressing rat model. Aging (Albany NY). 2025;17(10):2598–636. 41117843 10.18632/aging.206331 PMC12606970

[15] Kim J, Zhao T, Petralia RS, Yu Y, Peng H, Myers E, et al. mGRASP enables mapping mammalian synaptic connectivity with light microscopy. Nat Methods. 2011;9(1):96–102. 22138823 10.1038/nmeth.1784 PMC3424517

[16] Brown RE. Individual odors of rats are discriminable independently of changes in gonadal hormone levels. Physiol Behav. 1988;43(3):359–63. 3174848 10.1016/0031-9384(88)90199-0

[17] Ewels P, Magnusson M, Lundin S, Kaller M. MultiQC: summarize analysis results for multiple tools and samples in a single report. Bioinformatics. 2016;32(19):3047–8. 27312411 10.1093/bioinformatics/btw354 PMC5039924

[18] Patro R, Duggal G, Love MI, Irizarry RA, Kingsford C. Salmon provides fast and bias-aware quantification of transcript expression. Nat Methods. 2017;14(4):417–9. 28263959 10.1038/nmeth.4197 PMC5600148

[19] Vekris A, Pilalis E, Chatziioannou A, Petry KG. A computational pipeline for the extraction of actionable biological information from NGS-phage display experiments. Front Physiol. 2019;10:1160. 31607941 10.3389/fphys.2019.01160 PMC6769401

[20] Kohler S, Carmody L, Vasilevsky N, Jacobsen JOB, Danis D, Gourdine JP, et al. Expansion of the Human Phenotype Ontology (HPO) knowledge base and resources. Nucleic Acids Res. 2019;47(D1):D1018–27. 30476213 10.1093/nar/gky1105 PMC6324074

[21] Smith CL, Eppig JT. The mammalian phenotype ontology: enabling robust annotation and comparative analysis. Wiley Interdiscip Rev Syst Biol Med. 2009;1(3):390–9. 20052305 10.1002/wsbm.44 PMC2801442

[22] Fabregat A, Jupe S, Matthews L, Sidiropoulos K, Gillespie M, Garapati P, et al. The Reactome pathway knowledgebase. Nucleic Acids Res. 2018;46(D1):D649–55. 29145629 10.1093/nar/gkx1132 PMC5753187

[23] Poussin C, Mathis C, Alexopoulos LG, Messinis DE, Dulize RH, Belcastro V, et al. The species translation challenge-a systems biology perspective on human and rat bronchial epithelial cells. Sci Data. 2014;1:140009. 25977767 10.1038/sdata.2014.9 PMC4322580

[24] Franzen O, Gan LM, Bjorkegren JLM. PanglaoDB: a web server for exploration of mouse and human single-cell RNA sequencing data. Database J Biol Databases Curation. 2019;2019:046. 10.1093/database/baz046 PMC6450036 30951143

[25] Bindea G, Mlecnik B, Hackl H, Charoentong P, Tosolini M, Kirilovsky A, et al. ClueGO: a Cytoscape plug-in to decipher functionally grouped gene ontology and pathway annotation networks. Bioinformatics. 2009;25(8):1091–3. 19237447 10.1093/bioinformatics/btp101 PMC2666812

[26] Franz M, Rodriguez H, Lopes C, Zuberi K, Montojo J, Bader GD, et al. GeneMANIA update 2018. Nucleic Acids Res. 2018;46(W1):W60–4. 29912392 10.1093/nar/gky311 PMC6030815

[27] Dumitriu A, Golji J, Labadorf AT, Gao B, Beach TG, Myers RH, et al. Integrative analyses of proteomics and RNA transcriptomics implicate mitochondrial processes, protein folding pathways and GWAS loci in Parkinson disease. BMC Med Genomics. 2016;9:5. 26793951 10.1186/s12920-016-0164-y PMC4722694

[28] Papp EA, Leergaard TB, Calabrese E, Johnson GA, Bjaalie JG. Waxholm space atlas of the Sprague Dawley rat brain. Neuroimage. 2014;97:374–86. 24726336 10.1016/j.neuroimage.2014.04.001 PMC4160085

[29] Kim C, Ho DH, Suk JE, You S, Michael S, Kang J, et al. Neuron-released oligomeric alpha-synuclein is an endogenous agonist of TLR2 for paracrine activation of microglia. Nat Commun. 2013;4:1562. 23463005 10.1038/ncomms2534 PMC4089961

[30] Hughes CD, Choi ML, Ryten M, Hopkins L, Drews A, Botia JA, et al. Picomolar concentrations of oligomeric alpha-synuclein sensitizes TLR4 to play an initiating role in Parkinson’s disease pathogenesis. Acta Neuropathol. 2019;137(1):103–20. 30225556 10.1007/s00401-018-1907-y PMC6338693

[31] Trudler D, Nazor KL, Eisele YS, Grabauskas T, Dolatabadi N, Parker J, et al. Soluble alpha-synuclein-antibody complexes activate the NLRP3 inflammasome in hiPSC-derived microglia. Proc Natl Acad Sci U S A. 2021;118(15):e2025847118. 33833060 10.1073/pnas.2025847118 PMC8054017

[32] Elhadi SA, Congdon-Loeffler J, Harrosch E, Khatib D, Naamneh L, Schechter M, et al. α-synuclein activates the PI3K/AKT pathway to drive lipid droplets accumulation: implications for parkinson’s disease. bioRxiv. 2025. 10.1101/2025.03.27.645689

[33] Khan MR, Yin X, Kang SU, Mitra J, Wang H, Ryu T, et al. Enhanced mTORC1 signaling and protein synthesis in pathologic alpha-synuclein cellular and animal models of Parkinson’s disease. Sci Transl Med. 2023;15(724):eadd0499. 38019930 10.1126/scitranslmed.add0499

[34] Kremer T, Taylor KI, Siebourg-Polster J, Gerken T, Staempfli A, Czech C, et al. Longitudinal analysis of multiple neurotransmitter metabolites in cerebrospinal fluid in early Parkinson’s disease. Mov Disord. 2021;36(8):1972–8. 33942926 10.1002/mds.28608 PMC8453505

[35] Nemani VM, Lu W, Berge V, Nakamura K, Onoa B, Lee MK, et al. Increased expression of alpha-synuclein reduces neurotransmitter release by inhibiting synaptic vesicle reclustering after endocytosis. Neuron. 2010;65(1):66–79. 20152114 10.1016/j.neuron.2009.12.023 PMC3119527

[36] Huang L, Ren Y, Zeng Z, Ren H, Li S, He S, et al. Comparative study of striatum GABA concentrations and magnetic resonance spectroscopic imaging in Parkinson’s disease monkeys. BMC Neurosci. 2019;20(1):42. 31395015 10.1186/s12868-019-0522-8 PMC6686405

[37] Chassain C, Bielicki G, Keller C, Renou JP, Durif F. Metabolic changes detected in vivo by 1H MRS in the MPTP-intoxicated mouse. NMR Biomed. 2010;23(6):547–53. 20661872 10.1002/nbm.1504

[38] Muriel MP, Agid Y, Hirsch E. Plasticity of afferent fibers to striatal neurons bearing D1 dopamine receptors in Parkinson’s disease. Mov Disord. 2001;16(3):435–41. 11391736 10.1002/mds.1103

[39] Mortberg MA, Gentile JE, Nadaf NM, Vanderburg C, Simmons S, Dubinsky D, et al. A single-cell map of antisense oligonucleotide activity in the brain. Nucleic Acids Res. 2023;51(14):7109–24. 37188501 10.1093/nar/gkad371 PMC10415122

[40] Locascio JJ, Eberly S, Liao Z, Liu G, Hoesing AN, Duong K, et al. Association between alpha-synuclein blood transcripts and early, neuroimaging-supported Parkinson’s disease. Brain J Neurol. 2015;138(Pt 9):2659–71. 10.1093/brain/awv202 PMC4643625 26220939

[41] Kulczynska-Przybik A, Dulewicz M, Slowik A, Borawska R, Kulakowska A, Kochanowicz J, et al. The clinical significance of cerebrospinal fluid reticulon 4 (RTN4) levels in the differential diagnosis of neurodegenerative diseases. J Clin Med. 2021;10(22):5281. 34830564 10.3390/jcm10225281 PMC8622503

[42] Niederost B, Oertle T, Fritsche J, McKinney RA, Bandtlow CE. Nogo-A and myelin-associated glycoprotein mediate neurite growth inhibition by antagonistic regulation of RhoA and Rac1. J Neurosci. 2002;22(23):10368–76. 12451136 10.1523/JNEUROSCI.22-23-10368.2002 PMC6758757

[43] Filippini A, Mutti V, Faustini G, Longhena F, Ramazzina I, Rizzi F, et al. Extracellular clusterin limits the uptake of alpha-synuclein fibrils by murine and human astrocytes. Glia. 2021;69(3):681–96. 33045109 10.1002/glia.23920 PMC7821254

[44] Huber KM, Gallagher SM, Warren ST, Bear MF. Altered synaptic plasticity in a mouse model of fragile X mental retardation. Proc Natl Acad Sci U S A. 2002;99(11):7746–50. 12032354 10.1073/pnas.122205699 PMC124340

[45] Marvaldi L, Thongrong S, Kozlowska A, Irschick R, Pritz CO, Baumer B, et al. Enhanced axon outgrowth and improved long-distance axon regeneration in sprouty2 deficient mice. Dev Neurobiol. 2015;75(3):217–31. 25104556 10.1002/dneu.22224

[46] Wang MJ, Huang HY, Chiu TL, Chang HF, Wu HR. Peroxiredoxin 5 silencing sensitizes dopaminergic neuronal cells to rotenone via DNA damage-triggered ATM/p53/PUMA signaling-mediated apoptosis. Cells. 2019;9(1):22. 31861721 10.3390/cells9010022 PMC7016837

[47] Ayadi AE, Zigmond MJ, Smith AD. IGF-1 protects dopamine neurons against oxidative stress: association with changes in phosphokinases. Exp Brain Res. 2016;234(7):1863–73. 26894890 10.1007/s00221-016-4572-1 PMC4893922

[48] Tien LT, Lee YJ, Pang Y, Lu S, Lee JW, Tseng CH, et al. Neuroprotective effects of intranasal IGF-1 against neonatal lipopolysaccharide-induced neurobehavioral deficits and neuronal inflammation in the substantia nigra and locus coeruleus of juvenile rats. Dev Neurosci. 2017;39(6):443–59. 28787734 10.1159/000477898 PMC5799046

[49] Darvish H, Azcona LJ, Taghavi S, Firouzabadi SG, Tafakhori A, Alehabib E, et al. ANXA1 with anti-inflammatory properties might contribute to parkinsonism. Ann Neurol. 2021;90(2):319–23. 34180078 10.1002/ana.26148

[50] Fushiki A, Ng D, Lewis ZR, Yadav A, Saraiva T, Hammond LA, et al. A vulnerable subtype of dopaminergic neurons drives early motor deficits in parkinson's disease. bioRxiv [Preprint]. 2024:2024.12.20.629776. 10.1101/2024.12.20.629776

[51] Leterrier C. The axon initial segment: an updated viewpoint. J Neurosci. 2018;38(9):2135–45. 29378864 10.1523/JNEUROSCI.1922-17.2018 PMC6596274

[52] Leussis MP, Berry-Scott EM, Saito M, Jhuang H, de Haan G, Alkan O, et al. The ANK3 bipolar disorder gene regulates psychiatric-related behaviors that are modulated by lithium and stress. Biol Psychiatry. 2013;73(7):683–90. 23237312 10.1016/j.biopsych.2012.10.016

[53] Bieri G, Brahic M, Bousset L, Couthouis J, Kramer NJ, Ma R, et al. LRRK2 modifies alpha-syn pathology and spread in mouse models and human neurons. Acta Neuropathol. 2019;137(6):961–80. 30927072 10.1007/s00401-019-01995-0 PMC6531417

[54] Daher JP, Volpicelli-Daley LA, Blackburn JP, Moehle MS, West AB. Abrogation of alpha-synuclein-mediated dopaminergic neurodegeneration in LRRK2-deficient rats. Proc Natl Acad Sci U S A. 2014;111(25):9289–94. 24927544 10.1073/pnas.1403215111 PMC4078806

[55] Kang SJ, Kim SJ, Noh HR, Kim BJ, Kim JB, Jin U, et al. Neuronal ApoE regulates the cell-to-cell transmission of alpha-synuclein. Int J Mol Sci. 2022;23(15):8311. 35955451 10.3390/ijms23158311 PMC9369063

[56] Gilchrist SE, Goudarzi S, Hafizi S. Gas6 inhibits toll-like receptor-mediated inflammatory pathways in mouse microglia via Axl and Mer. Front Cell Neurosci. 2020;14:576650. 33192322 10.3389/fncel.2020.576650 PMC7584110

[57] Abeliovich A, Schmitz Y, Farinas I, Choi-Lundberg D, Ho WH, Castillo PE, et al. Mice lacking alpha-synuclein display functional deficits in the nigrostriatal dopamine system. Neuron. 2000;25(1):239–52. 10707987 10.1016/s0896-6273(00)80886-7

[58] Somayaji M, Cataldi S, Choi SJ, Edwards RH, Mosharov EV, Sulzer D. A dual role for alpha-synuclein in facilitation and depression of dopamine release from substantia nigra neurons in vivo. Proc Natl Acad Sci U S A. 2020;117(51):32701–10. 33273122 10.1073/pnas.2013652117 PMC7768743

[59] Crooke ST. Molecular mechanisms of antisense oligonucleotides. Nucleic Acid Ther. 2017;27(2):70–7. 28080221 10.1089/nat.2016.0656 PMC5372764

[60] Doxakis E. Therapeutic antisense oligonucleotides for movement disorders. Med Res Rev. 2021;41(5):2656–88. 32656818 10.1002/med.21706

[61] Zanardi TA, Kim TW, Shen L, Serota D, Papagiannis C, Park SY, et al. Chronic toxicity assessment of 2’-o-methoxyethyl antisense oligonucleotides in mice. Nucleic Acid Ther. 2018;28(4):233–41. 29708844 10.1089/nat.2017.0706

[62] Kim TW, Papagiannis CN, Zwick LS, Engelhardt JA, Hoffmaster CM, Post NM, et al. Comparison of the class effects of antisense oligonucleotides in CByB6F1-Tg(HRAS)2Jic and CD-1 mice. Toxicol Pathol. 2019;47(1):82–92. 30585133 10.1177/0192623318813143

[63] Palaiologou A, Naki M, Pantazopoulou M, Kattan FG, Stefanis L, Doxakis E, et al. A bioanalytical liquid chromatography tandem mass spectrometry approach for the quantification of a novel antisense oligonucleotide designed for Parkinson’s disease: a rat brain biodistribution study. ACS Pharmacol Transl Sci. 2025;8(2):592–601. 39974630 10.1021/acsptsci.4c00698 PMC11833717

[64] Cooper JM, Wiklander PB, Nordin JZ, Al-Shawi R, Wood MJ, Vithlani M, et al. Systemic exosomal siRNA delivery reduced alpha-synuclein aggregates in brains of transgenic mice. Mov Disord Off J Mov Disord Soc. 2014;29(12):1476–85. 10.1002/mds.25978 PMC4204174 25112864

[65] Lewis J, Melrose H, Bumcrot D, Hope A, Zehr C, Lincoln S, et al. In vivo silencing of alpha-synuclein using naked siRNA. Mol Neurodegener. 2008;3:19. 18976489 10.1186/1750-1326-3-19 PMC2612658

[66] Zharikov AD, Cannon JR, Tapias V, Bai Q, Horowitz MP, Shah V, et al. shRNA targeting alpha-synuclein prevents neurodegeneration in a Parkinson’s disease model. J Clin Investig. 2015;125(7):2721–35. 26075822 10.1172/JCI64502 PMC4563670

[67] Schenk DB, Koller M, Ness DK, Griffith SG, Grundman M, Zago W, et al. First-in-human assessment of PRX002, an anti-alpha-synuclein monoclonal antibody, in healthy volunteers. Mov Disord. 2017;32(2):211–8. 10.1002/mds.26878 PMC5324684 27886407

[68] Weihofen A, Liu Y, Arndt JW, Huy C, Quan C, Smith BA, et al. Development of an aggregate-selective, human-derived alpha-synuclein antibody BIIB054 that ameliorates disease phenotypes in Parkinson’s disease models. Neurobiol Dis. 2019;124:276–88. 30381260 10.1016/j.nbd.2018.10.016

[69] Brys M, Fanning L, Hung S, Ellenbogen A, Penner N, Yang M, et al. Randomized phase I clinical trial of anti-alpha-synuclein antibody BIIB054. Mov Disord. 2019;34(8):1154–63. 10.1002/mds.27738 PMC6771554 31211448

