# Supplementary material for: Brain region-resolved pharmacodynamics of an antisense oligonucleotide targeting human SNCA 3′UTR in BAC-hSNCA rats
Source: Transl Neurodegener. 2026 Sep 16;15:47. doi: 10.1186/s40035-026-00577-x (PMC13579807; doi:10.1186/s40035-026-00577-x)

Supplementary File 5: Uncropped blots

Brain region-resolved pharmacodynamics of an antisense oligonucleotide targeting human *SNCA* 3'UTR in BAC-h*SNCA* rats

Marianna Naki, Fedon-Giasin Kattan, Marina Pantazopoulou, Sissy Skea, Dimitrios Zisis, Eleftherios Pilalis, Maria Fouka, Effrosyni Koronaiou, Christos Fotis, Olaf Riess, Aristotelis Chatziioannou, Leonidas G Alexopoulos, Leonidas Stefanis and Epaminondas Doxakis

Supplementary File 5. Uncropped blots corresponding to Figure 1c, 1h, 6b, S2a, S2b, S4, S5a,S5c, S5e as labeled. Nippon Blue Star prestained protein marker was used in all blots. Dotted lines represent approximate cropped region used as a representative image in corresponding figure. ^= Additional samples on membrane irrelevant to the final experiment and analysis.

Figure 1c

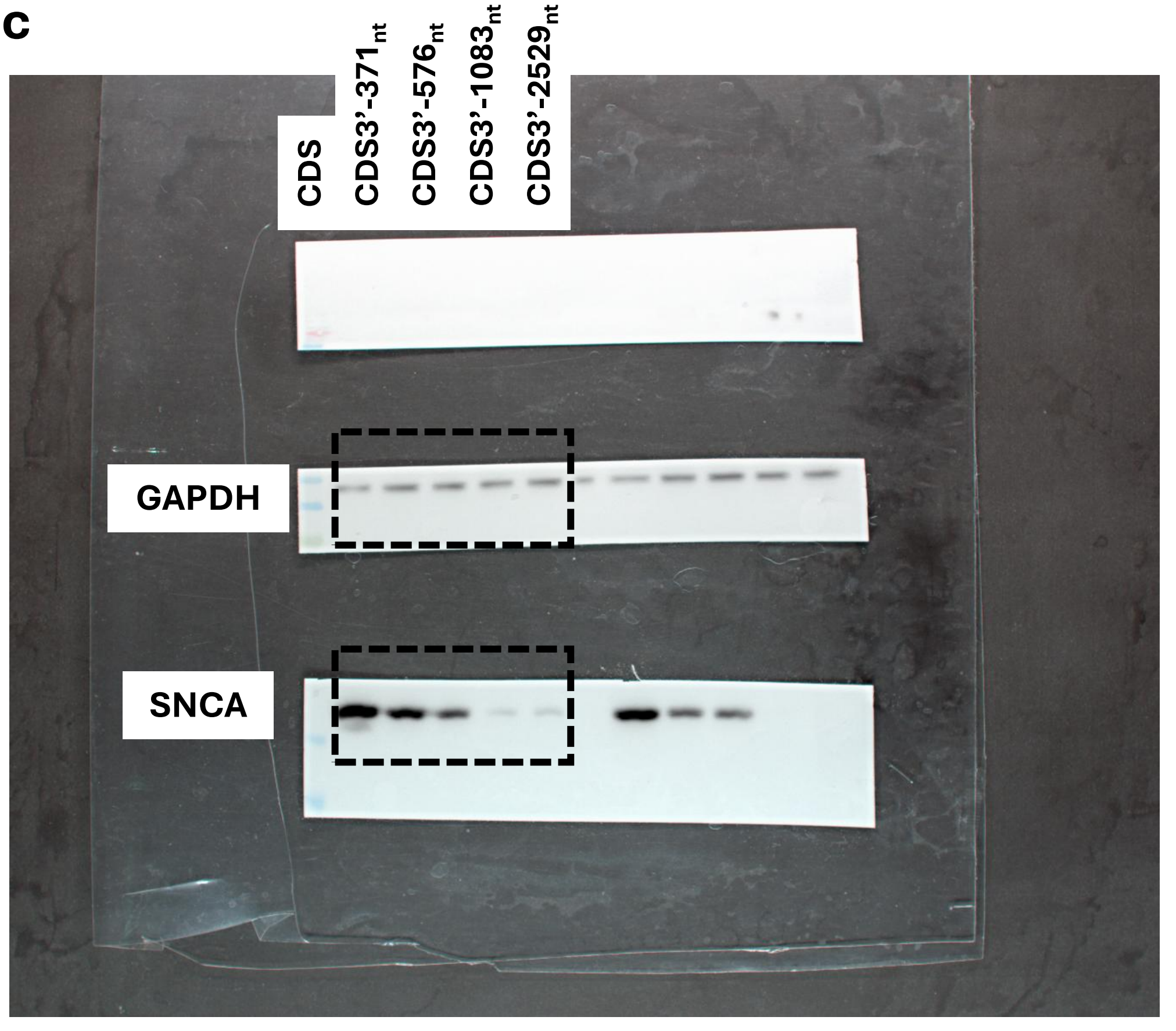
CDS
CDS3'-371nt
CDS3'-576nt
CDS3'-1083nt
CDS3'-2529nt
GAPDH
SNCA

Figure 1h

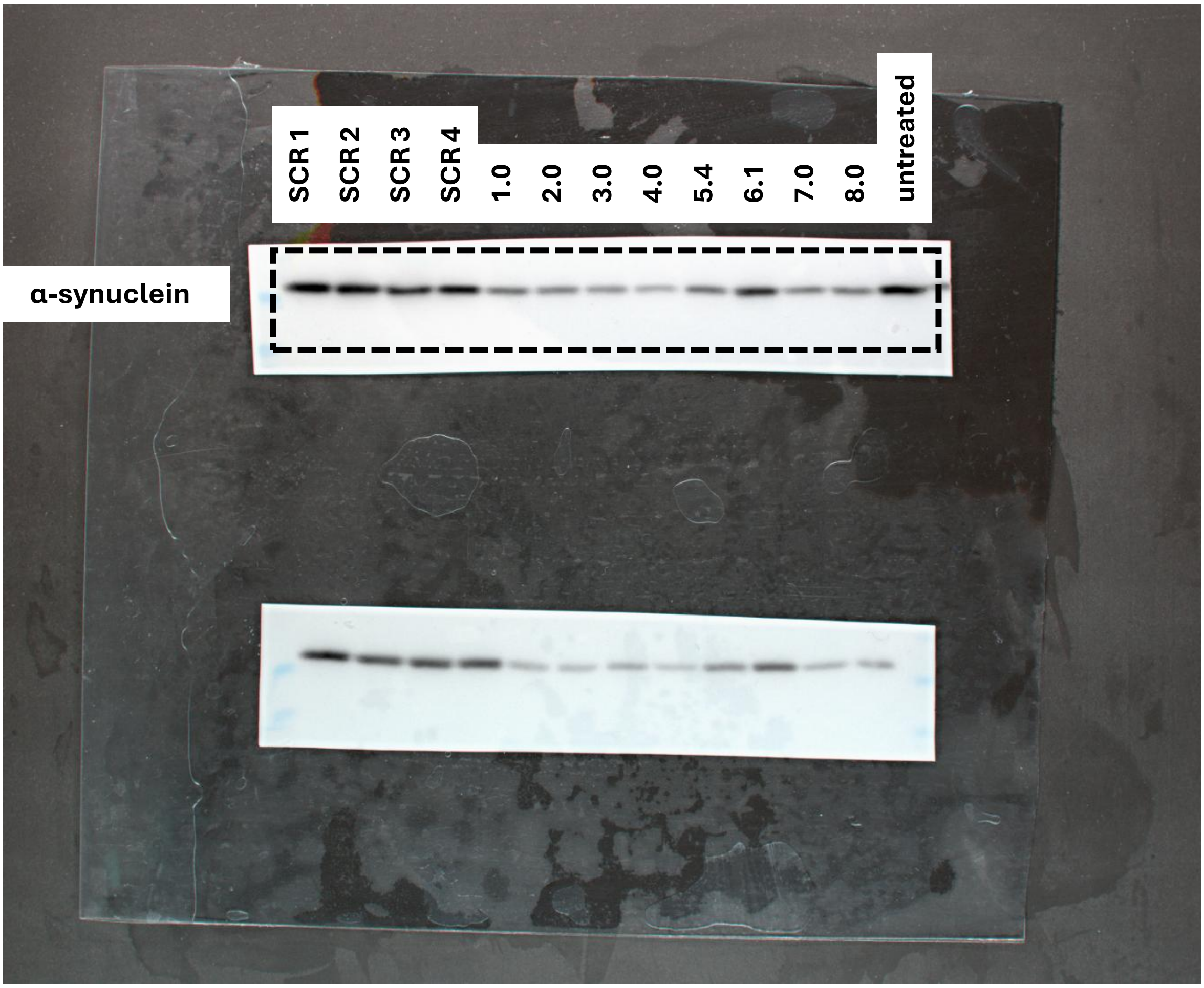
SCR 1
SCR 2
SCR 3
SCR 4
1.0
2.0
3.0
4.0
5.4
6.1
7.0
8.0
untreated
α-synuclein

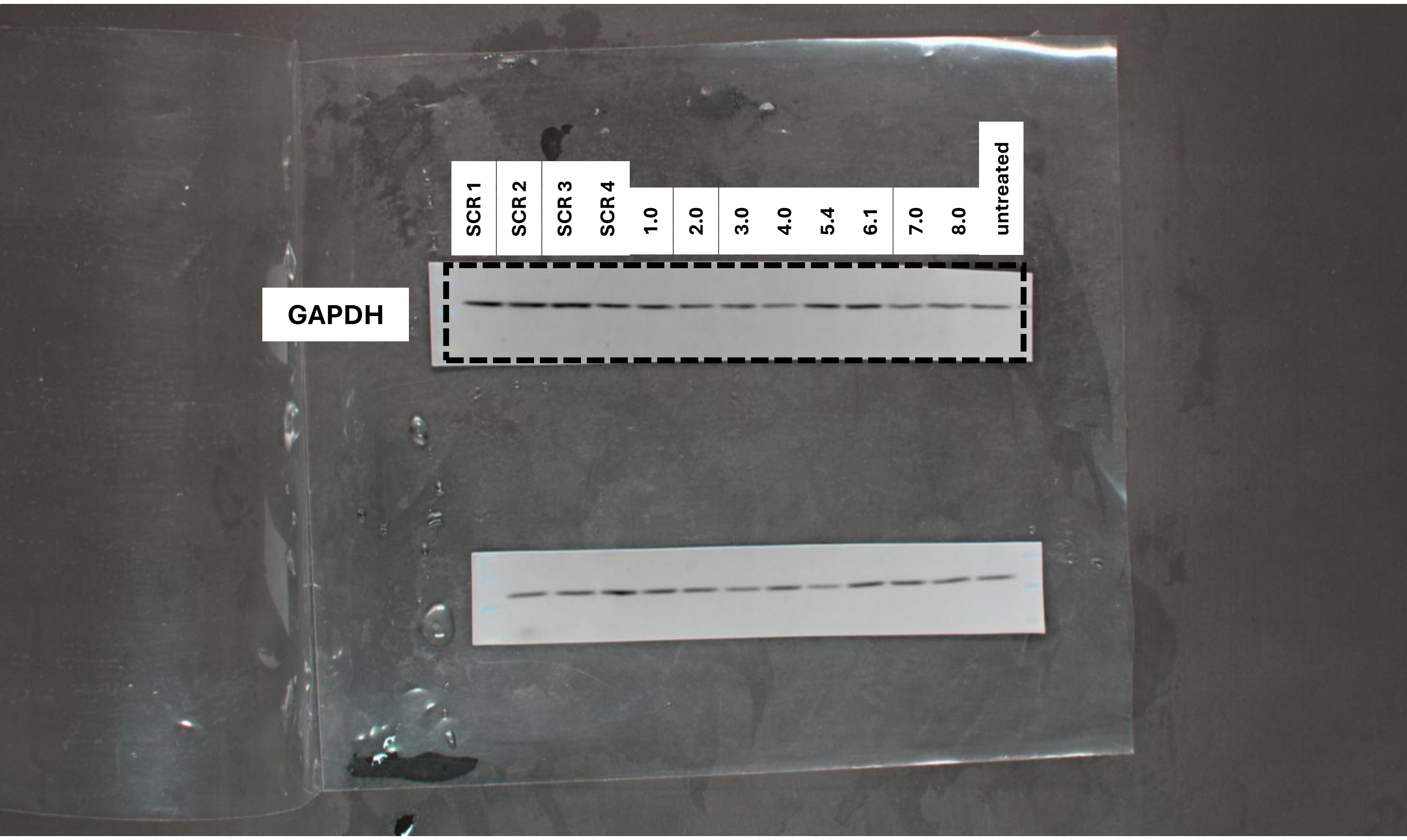
SCR 1
SCR 2
SCR 3
SCR 4
1.0
2.0
3.0
4.0
5.4
6.1
7.0
8.0
untreated
GAPDH

Figure 6b
striatum

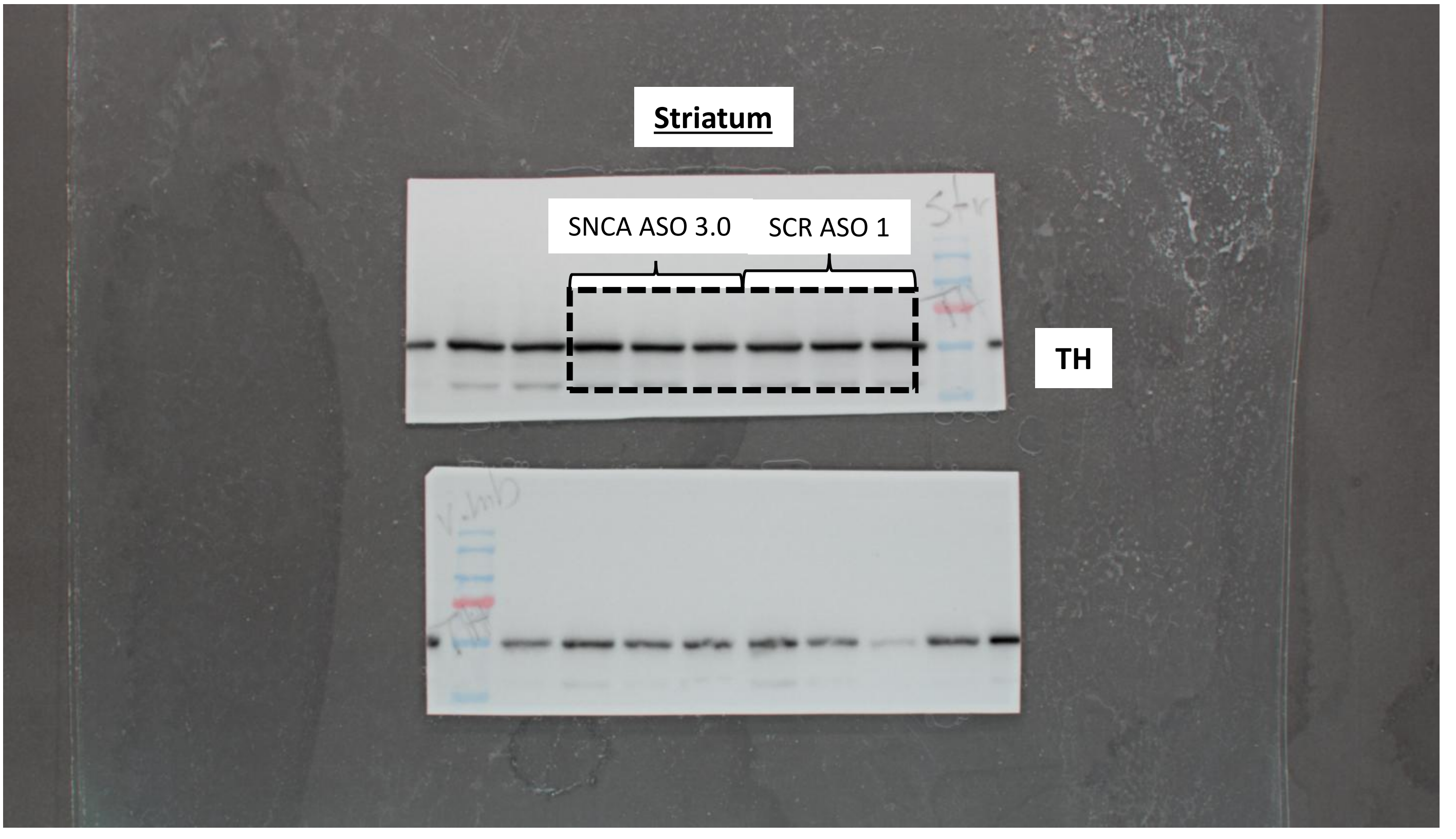

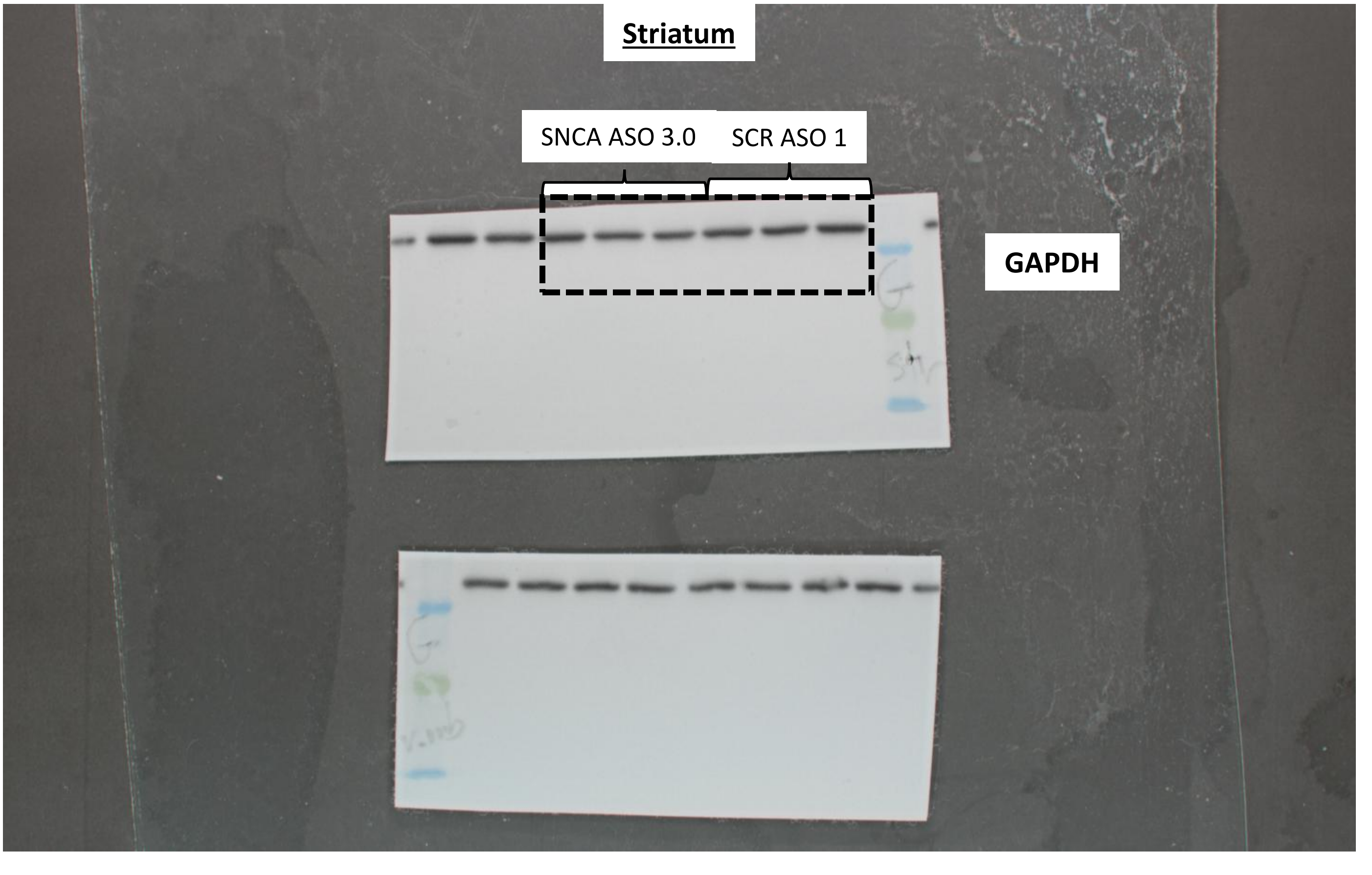

Figure 6b
Ventral midbrain

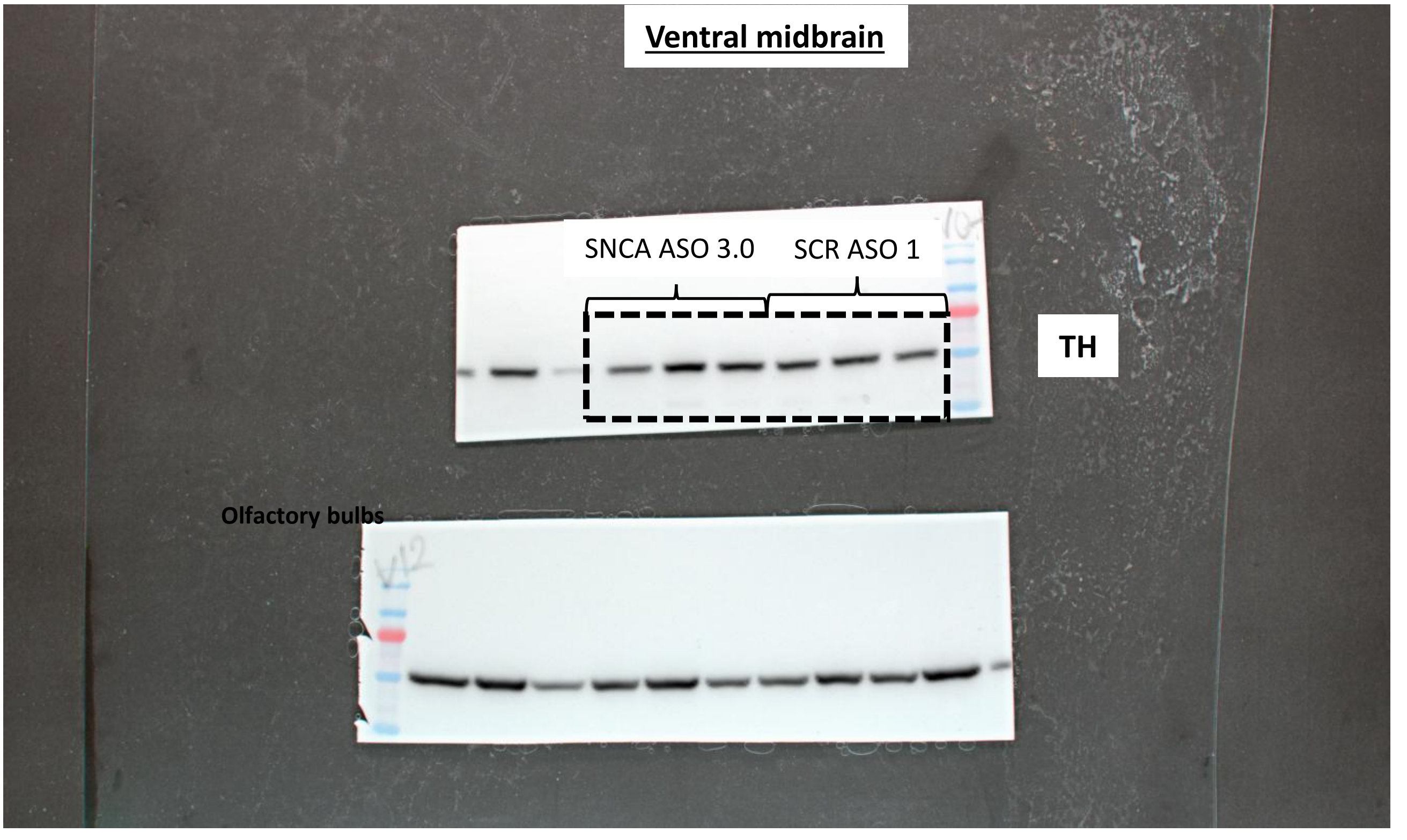

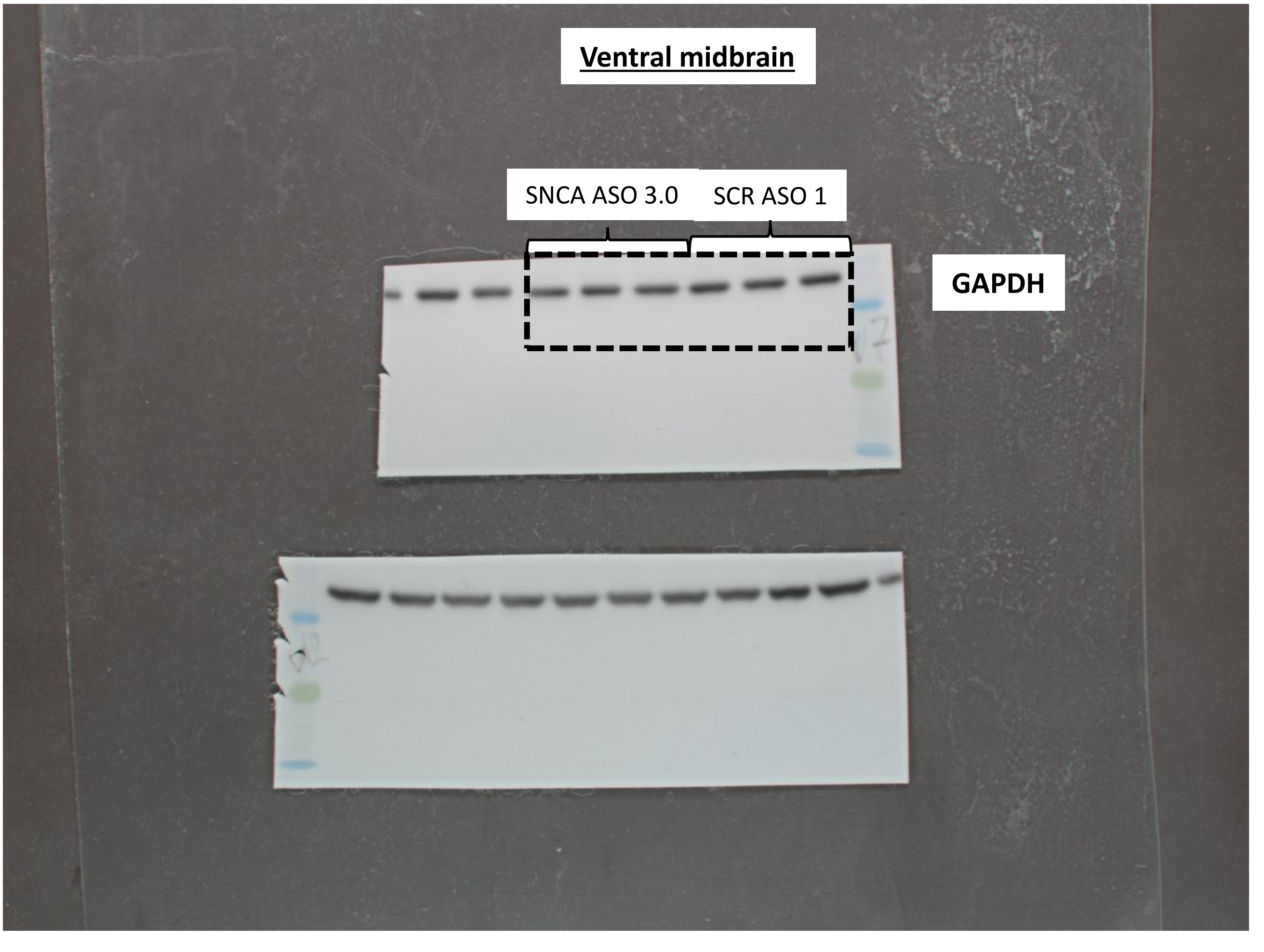

Figure S2a

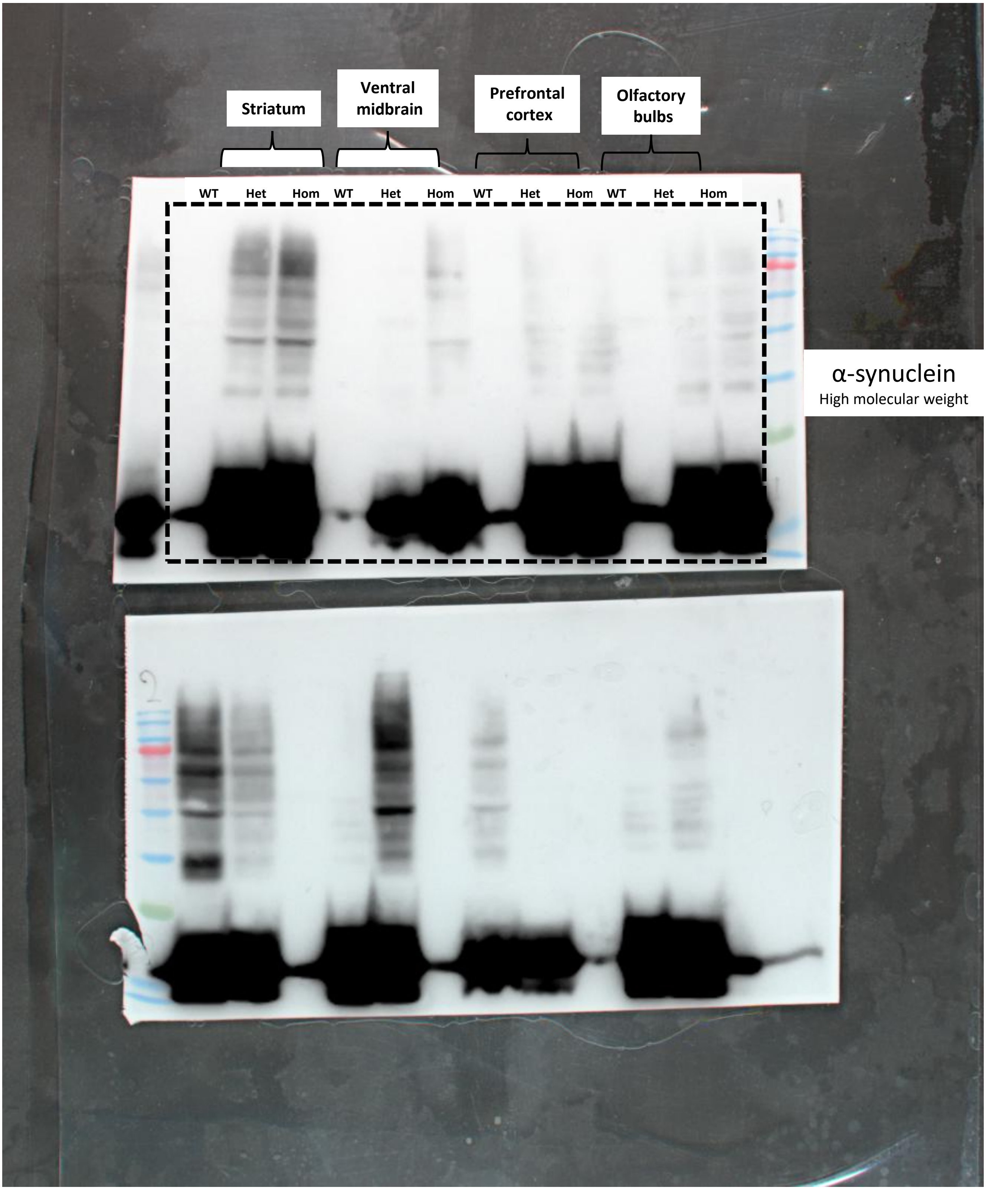

Striatum
Ventral midbrain
Prefrontal cortex
Olfactory bulbs
WT Het Hom WT Het Hom WT Het Hom WT Het Hom
α-synuclein
High molecular weight

Figure S2a

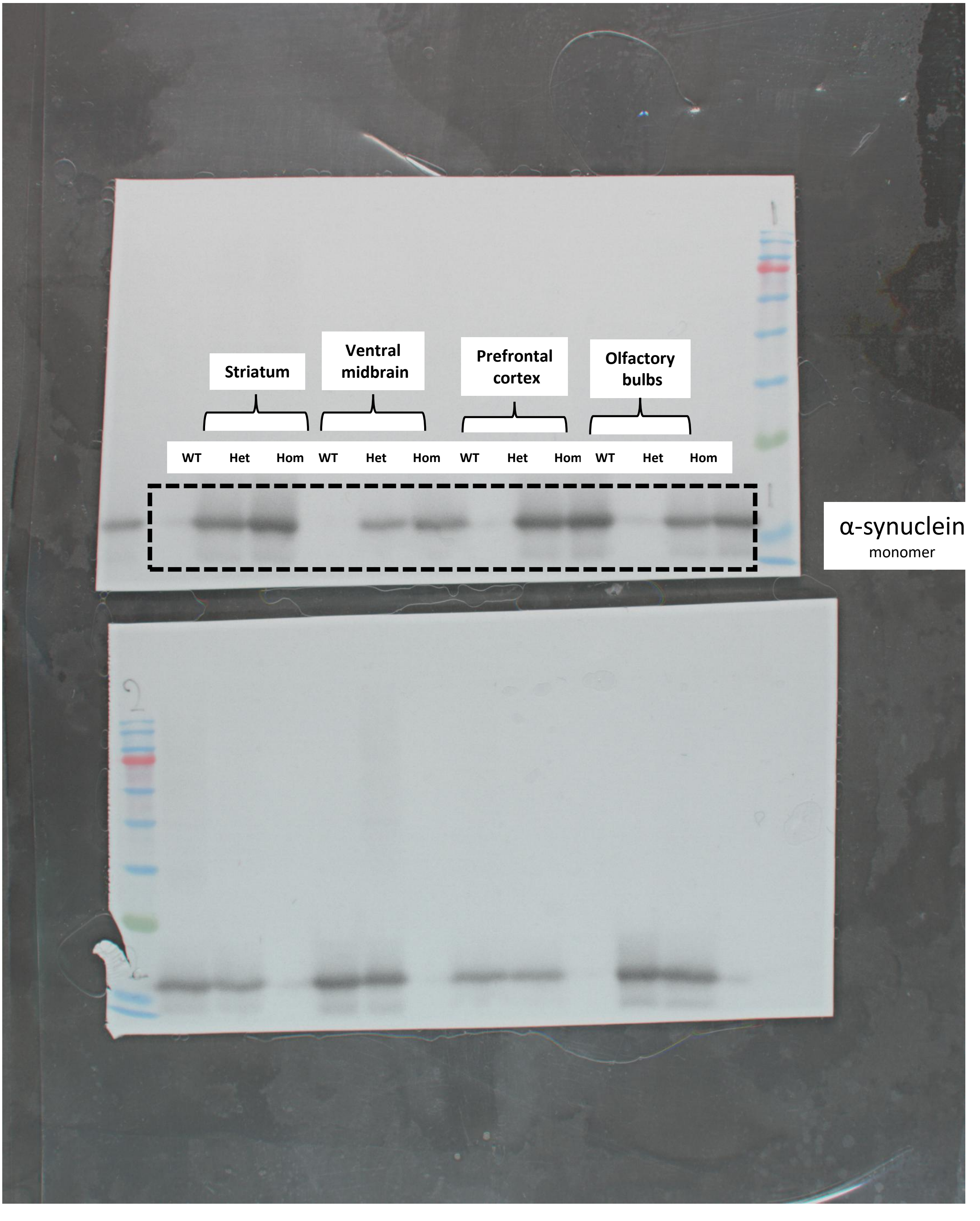
Striatum
Ventral midbrain
Prefrontal cortex
Olfactory bulbs
WT Het Hom WT Het Hom WT Het Hom WT Het Hom
α-synuclein
monomer

Figure S2

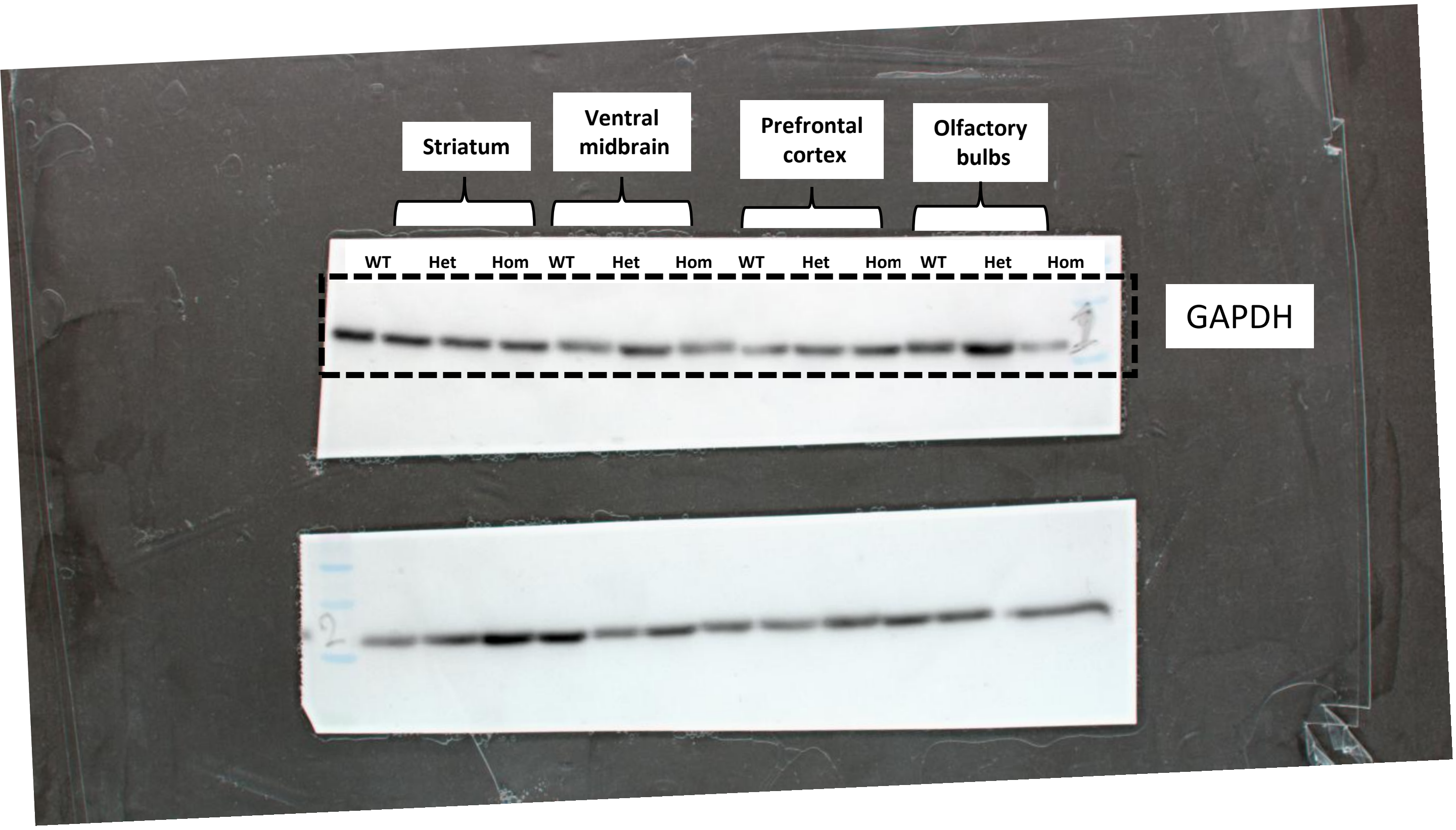
Striatum
Ventral midbrain
Prefrontal cortex
Olfactory bulbs
WT
Het
Hom
WT
Het
Hom
WT
Het
Hom
WT
Het
Hom
GAPDH

Figure S2b

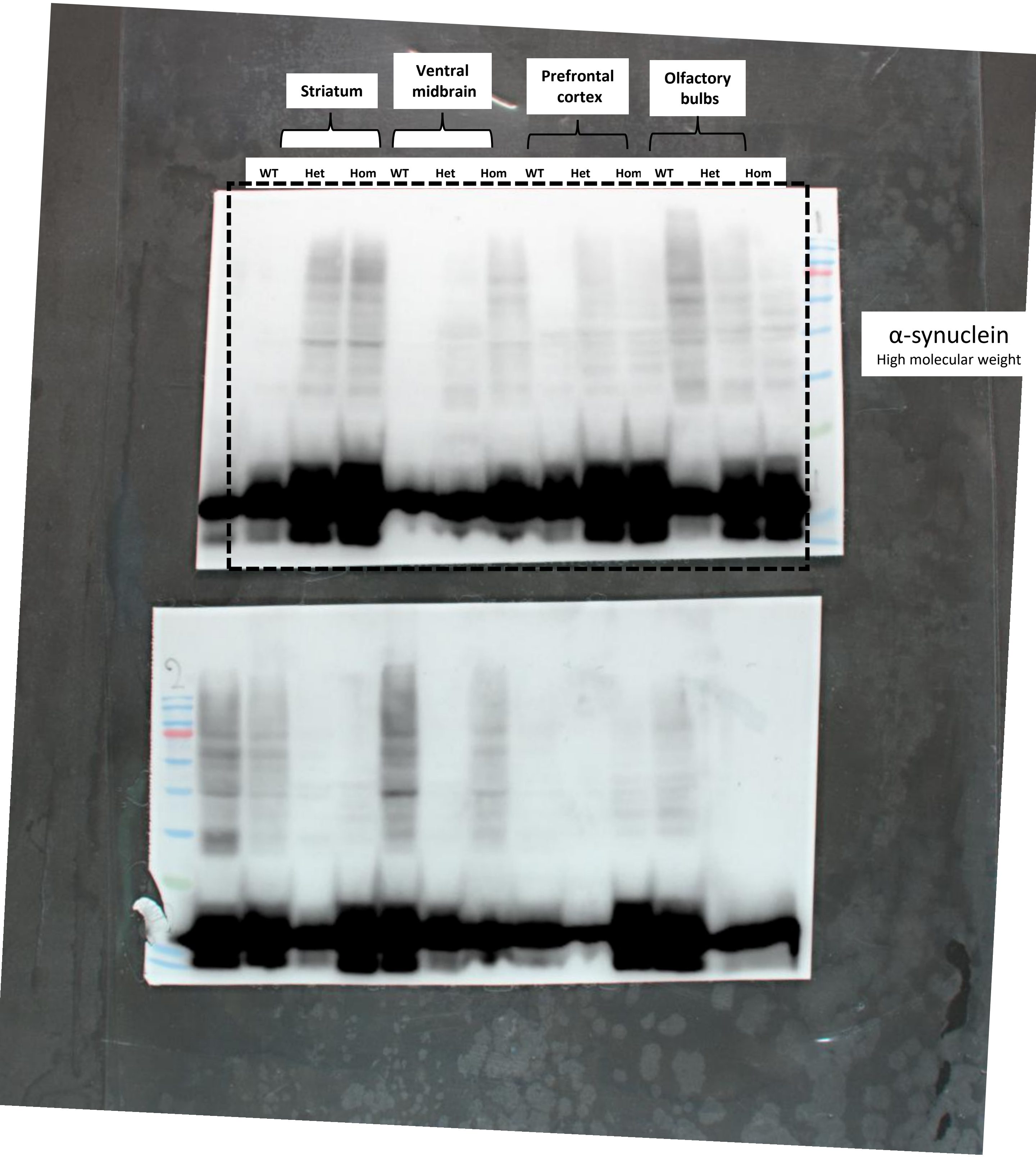
Striatum
Ventral midbrain
Prefrontal cortex
Olfactory bulbs
WT Het Hom WT Het Hom WT Het Hom WT Het Hom
α-synuclein
High molecular weight

Figure S2b

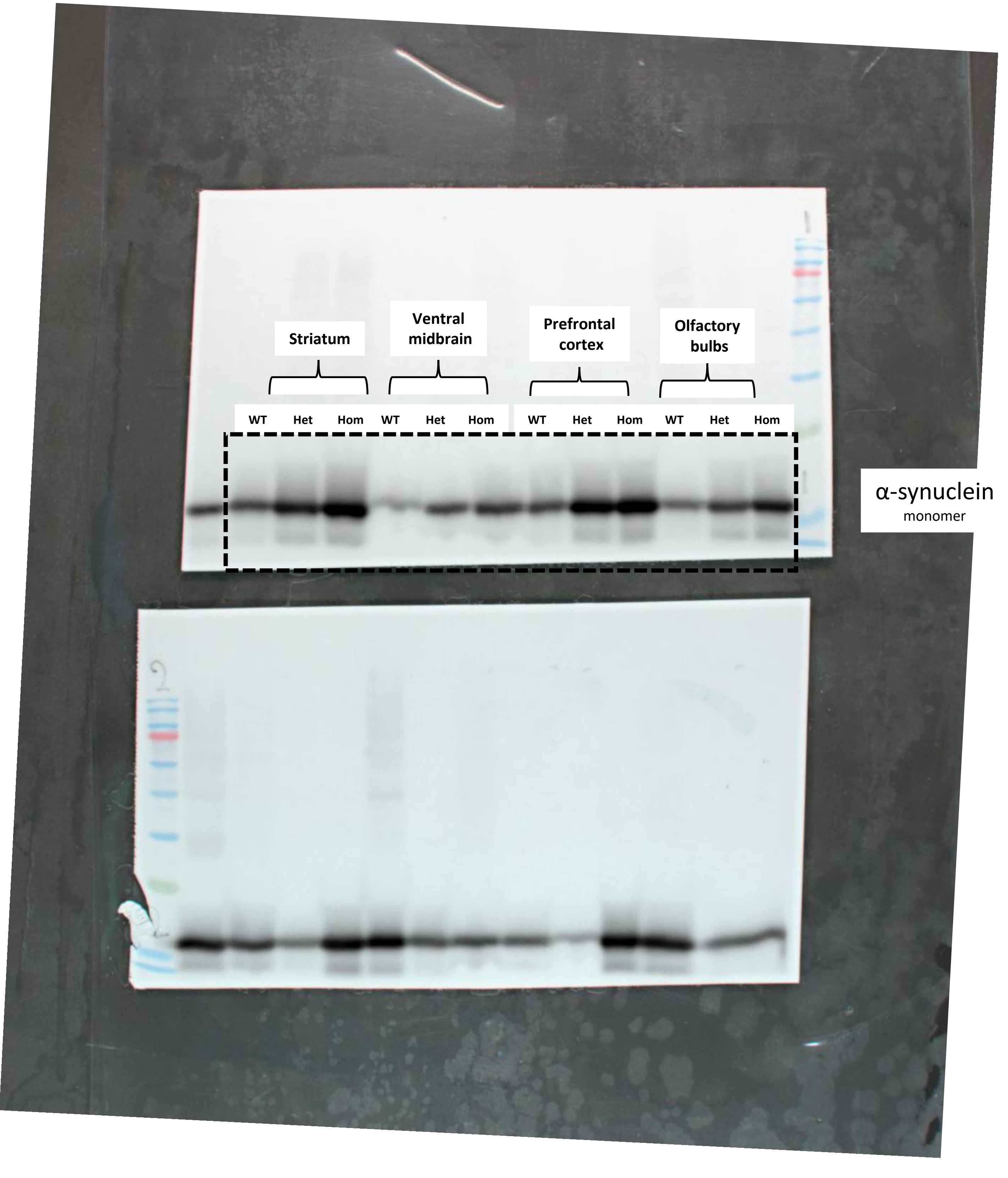

Striatum
Ventral midbrain
Prefrontal cortex
Olfactory bulbs
WT Het Hom WT Het Hom WT Het Hom WT Het Hom
α-synuclein
monomer

Figure S4

Olfactory bulbs

Triton X-100 soluble fraction

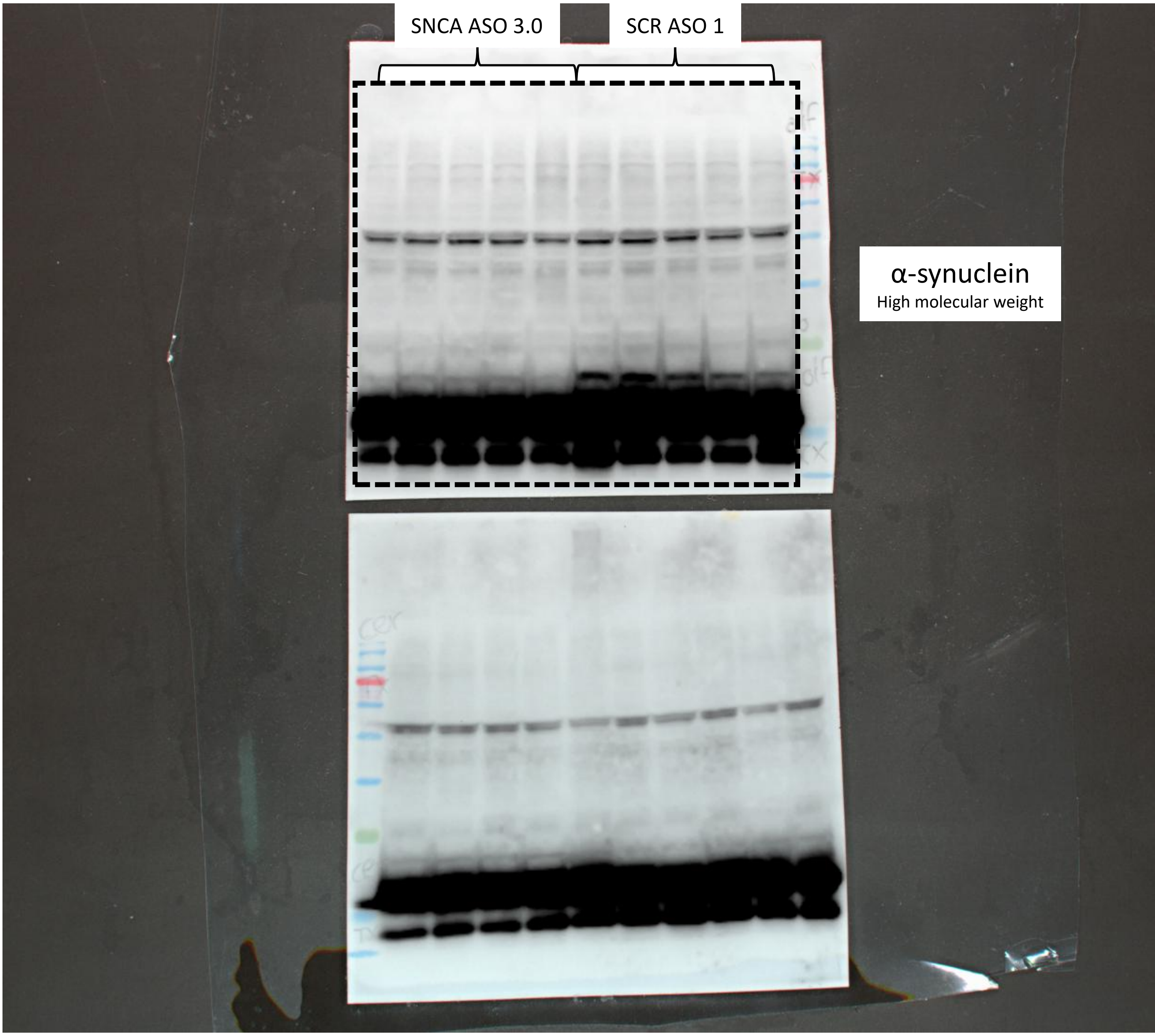

Figure S4

Olfactory bulbs

Triton X-100 soluble fraction

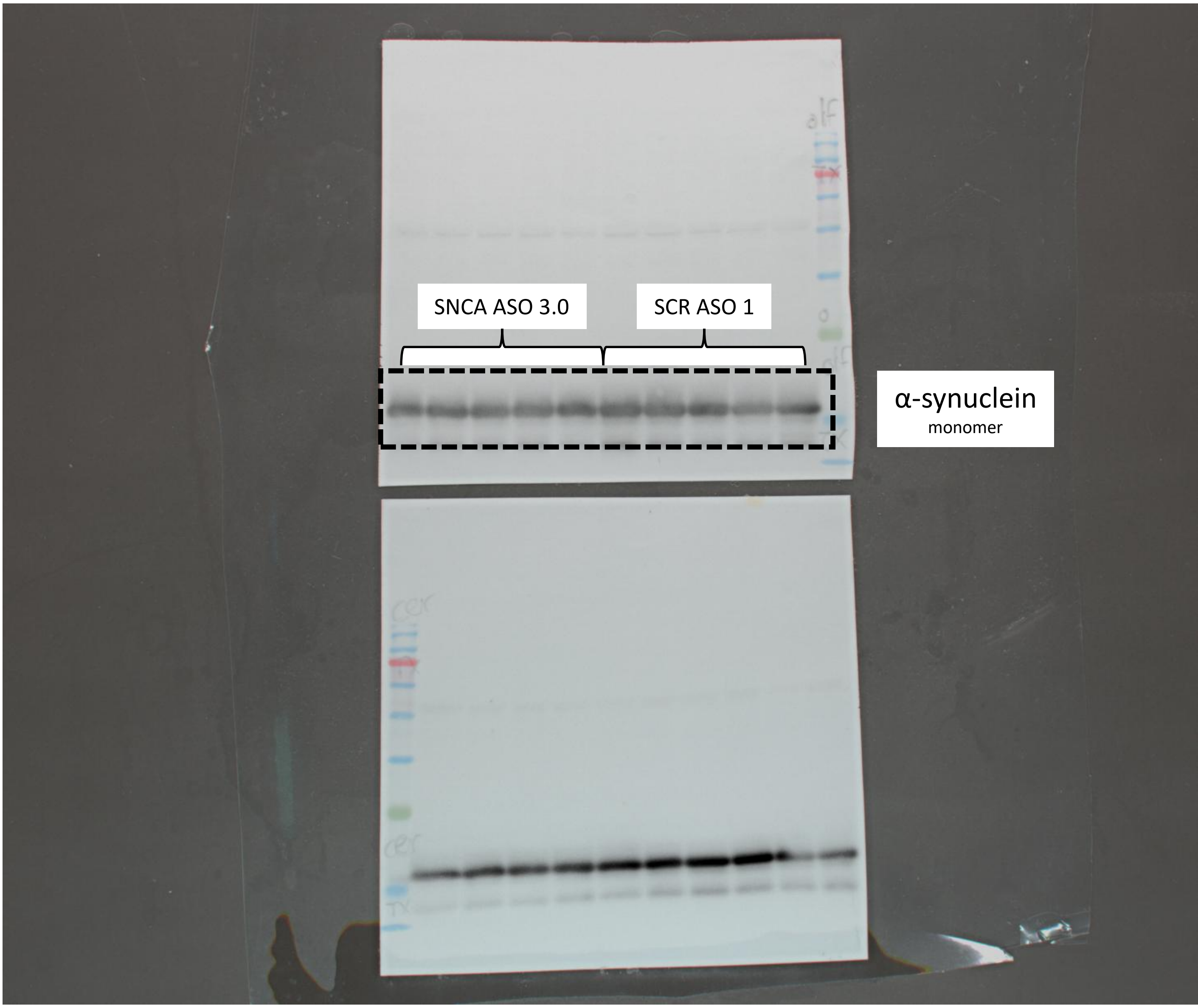

Figure S4

Olfactory bulbs

Triton X-100 soluble fraction

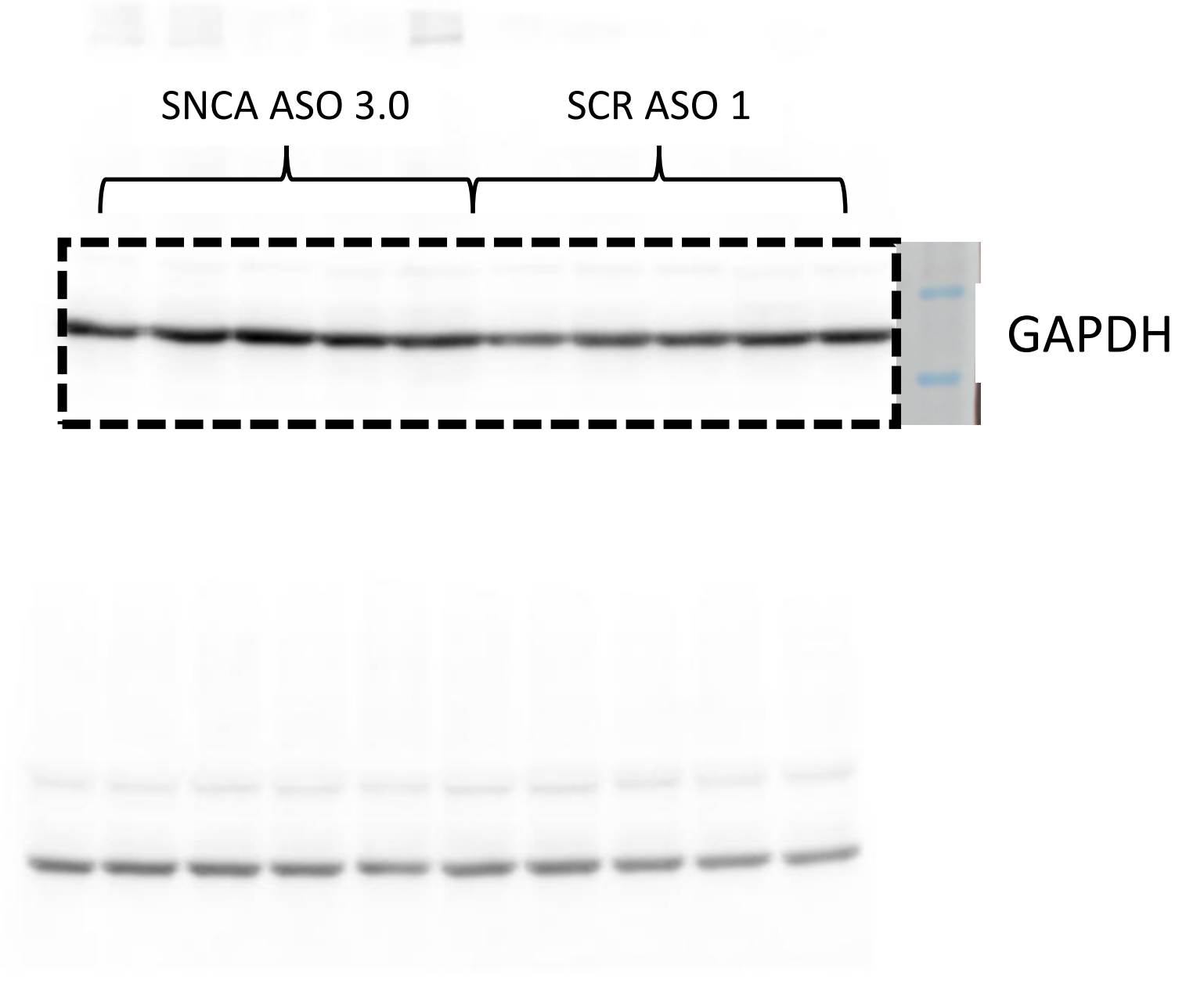

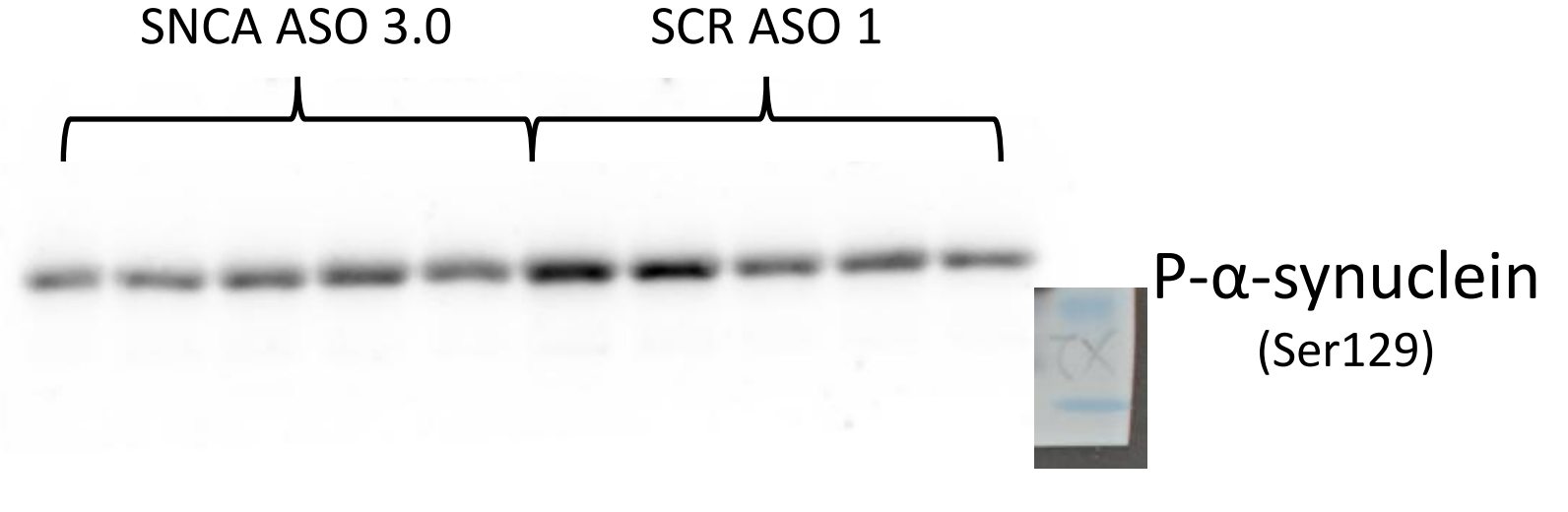

Figure S4

Olfactory bulbs
SDS insoluble fraction

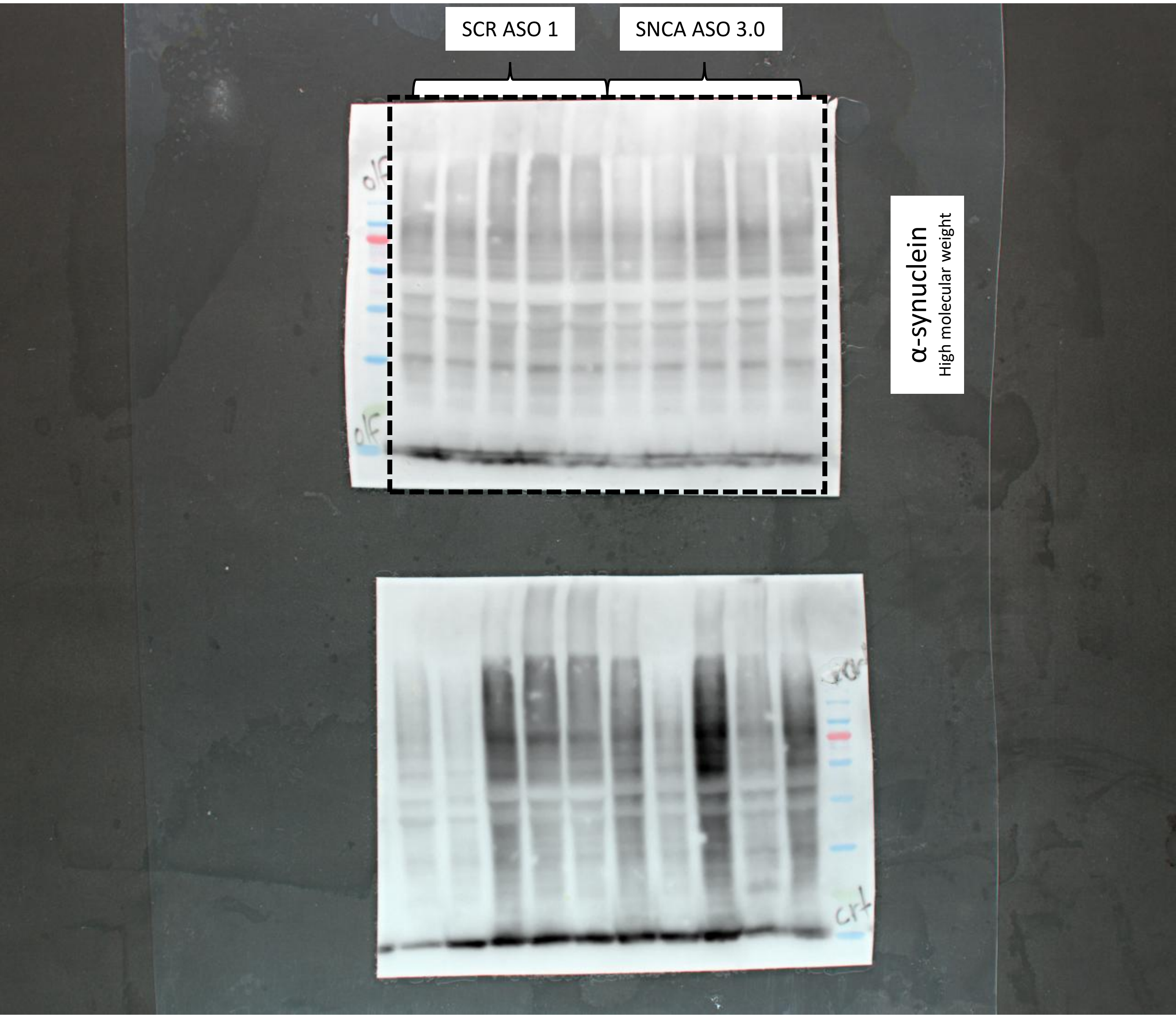

Figure S4

Olfactory bulbs
SDS insoluble fraction

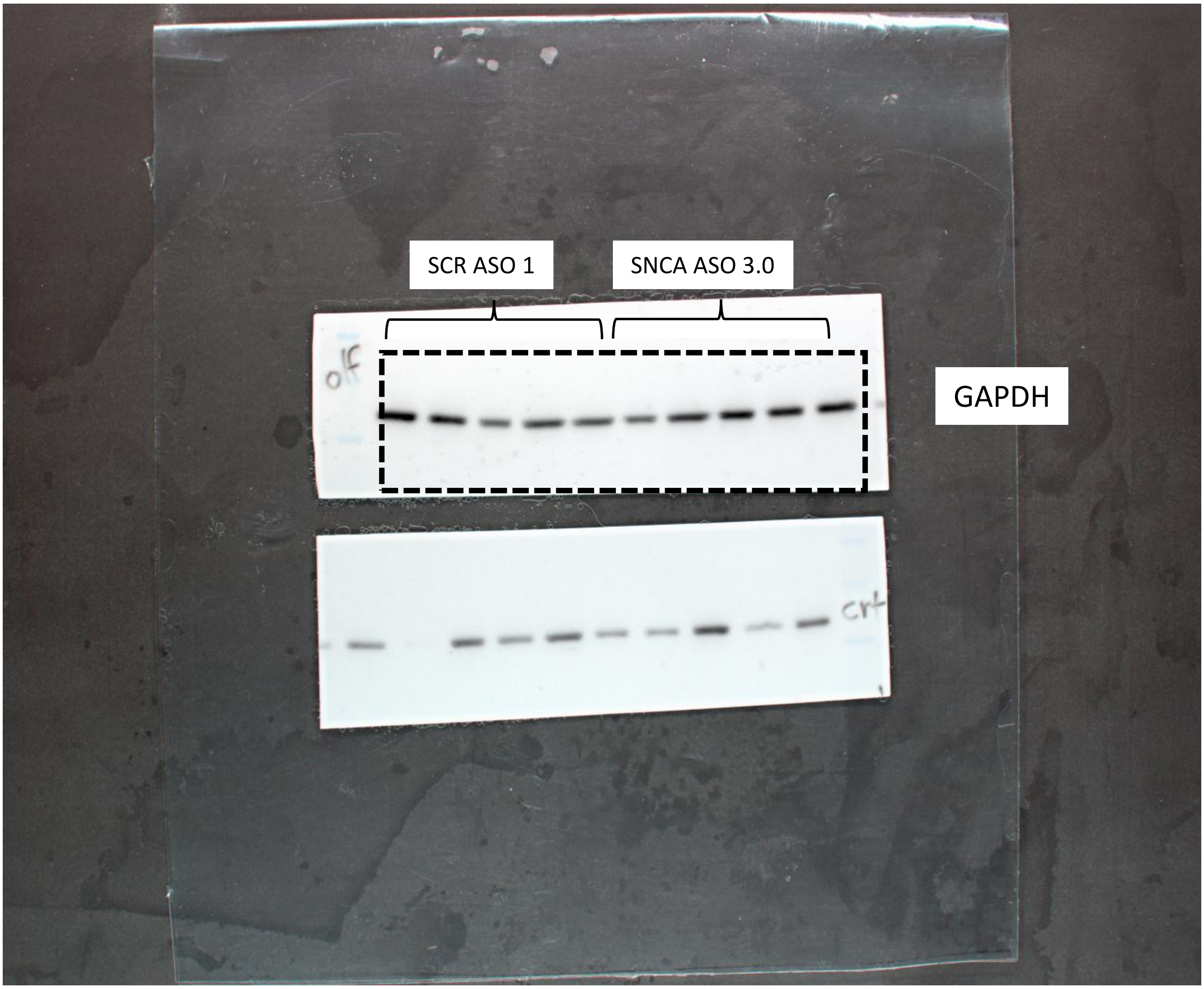

Figure S4

Olfactory bulbs

SDS insoluble fraction

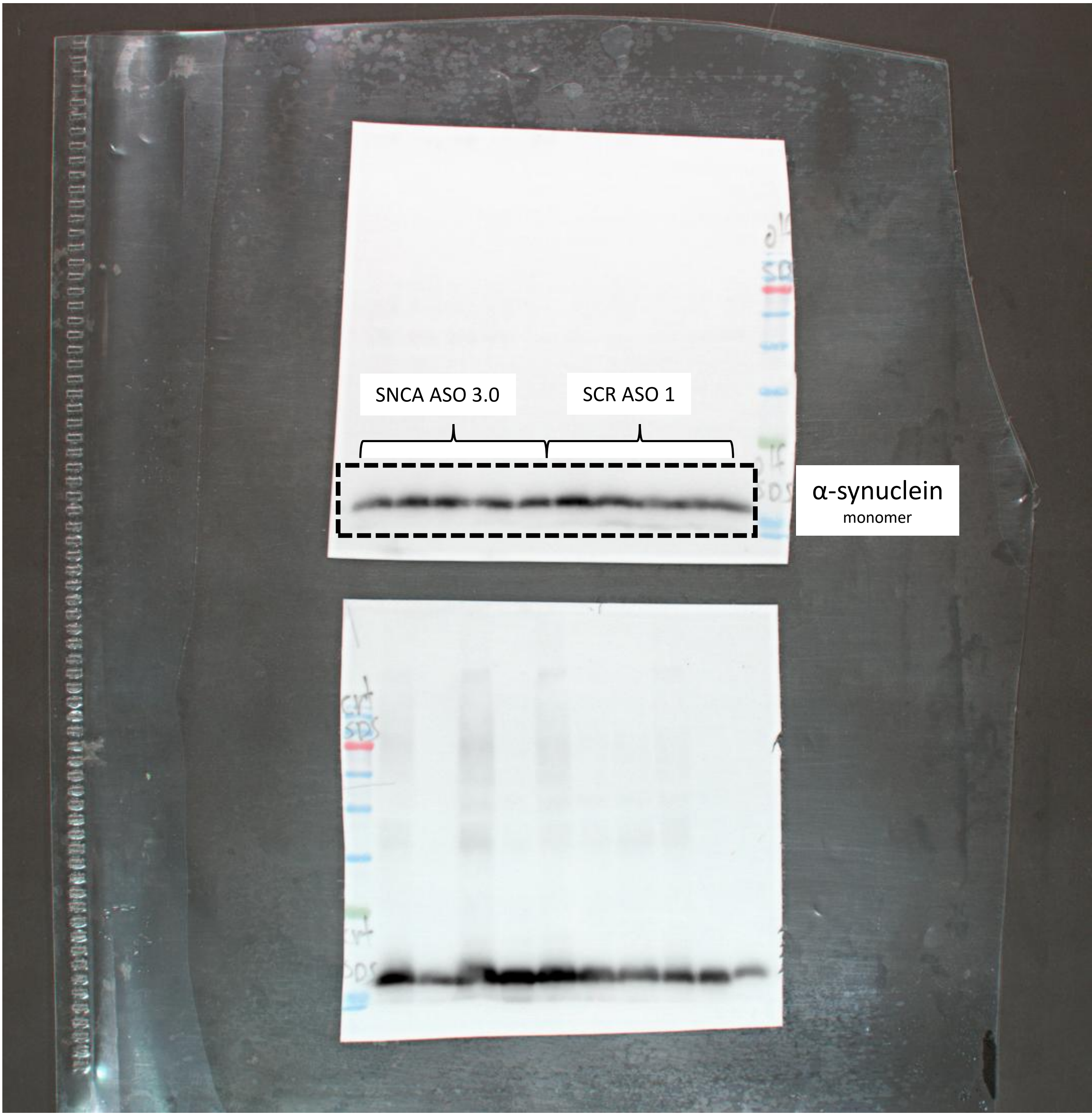

Figure S4

Prefrontal Cortex

Triton X-100 soluble fraction

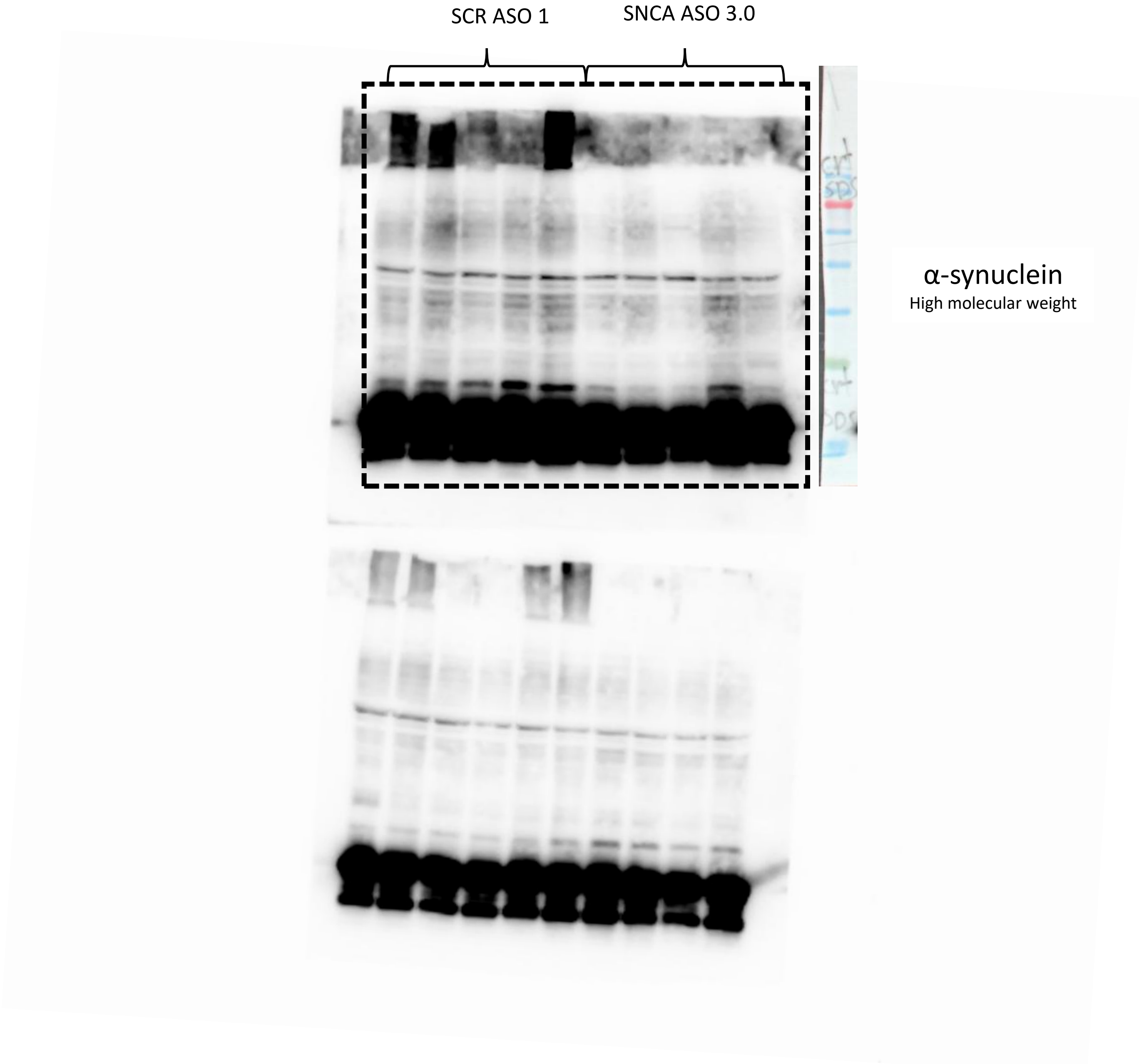

Figure S4

Prefrontal Cortex

Triton X-100 soluble fraction

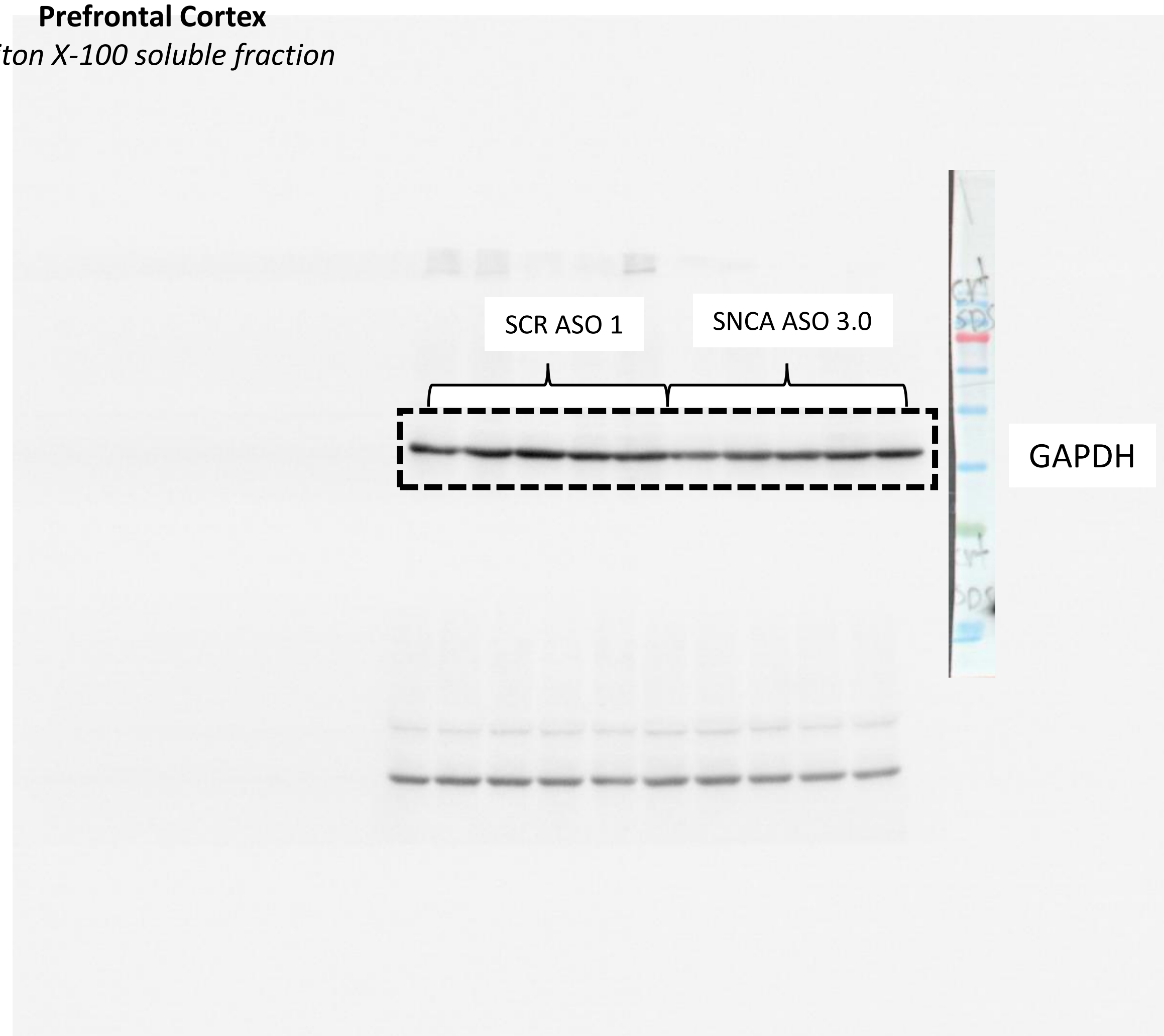

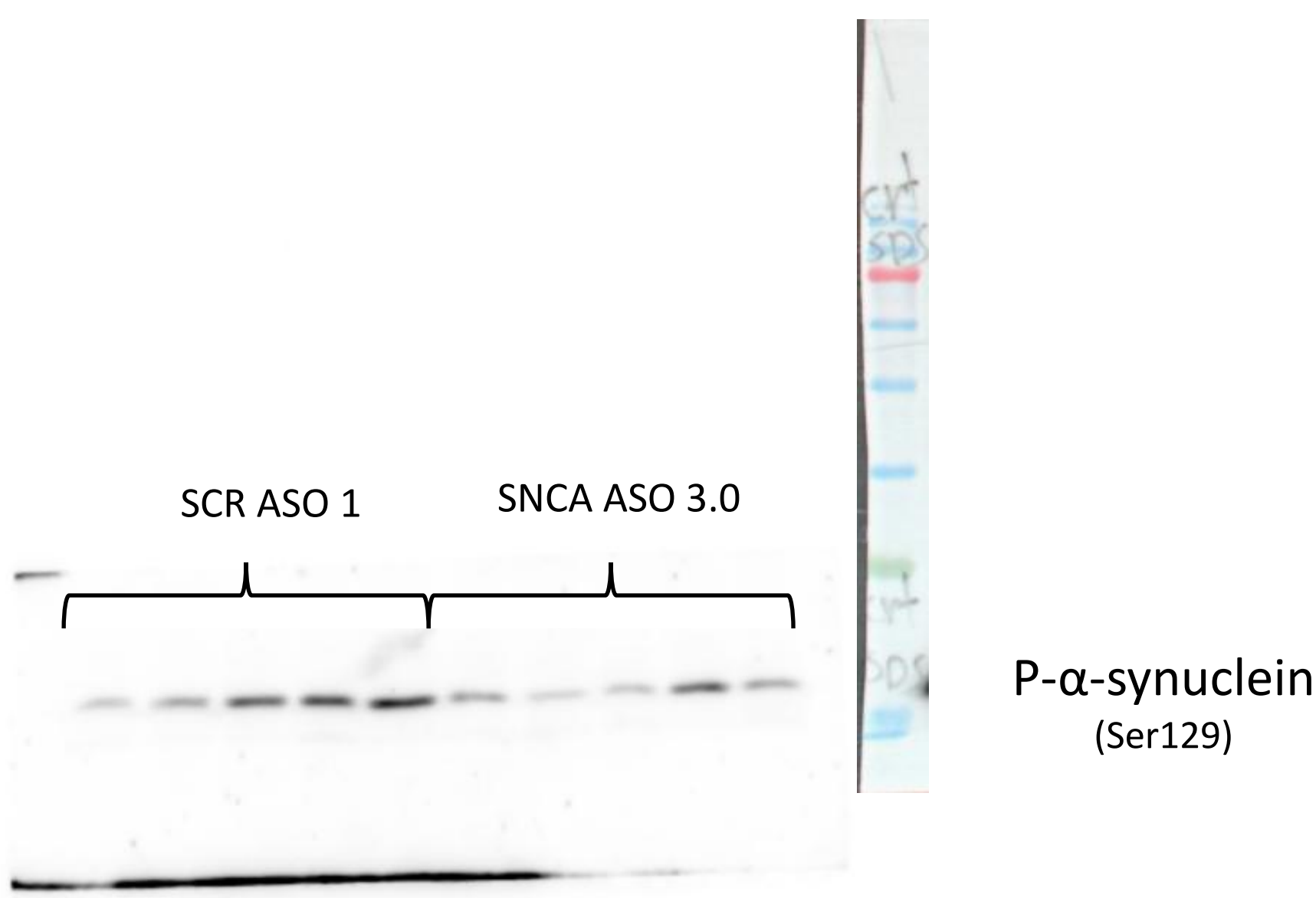

Figure S4

Prefrontal Cortex

SDS insoluble fraction

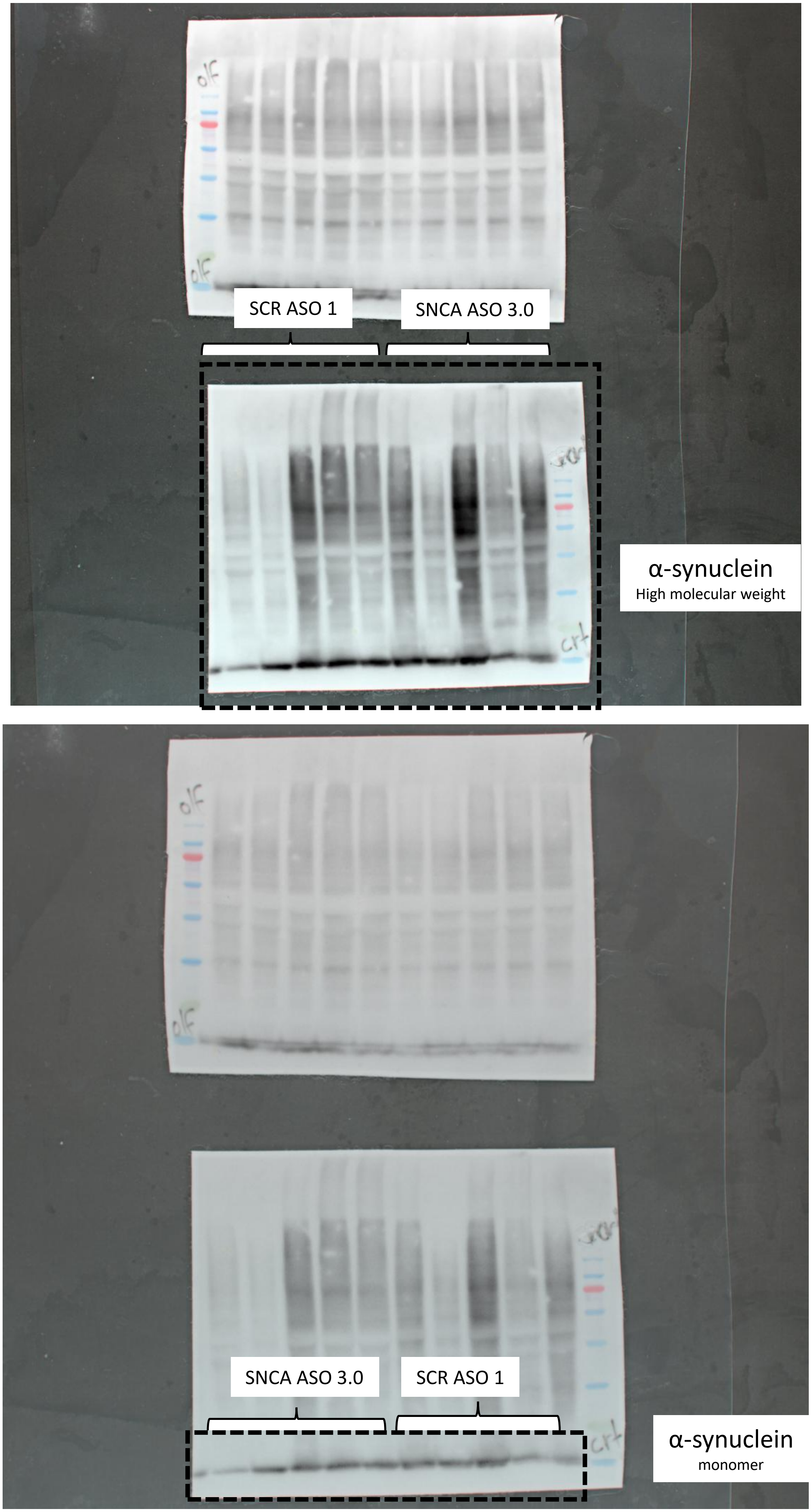

Figure S4

Prefrontal Cortex
SDS insoluble fraction

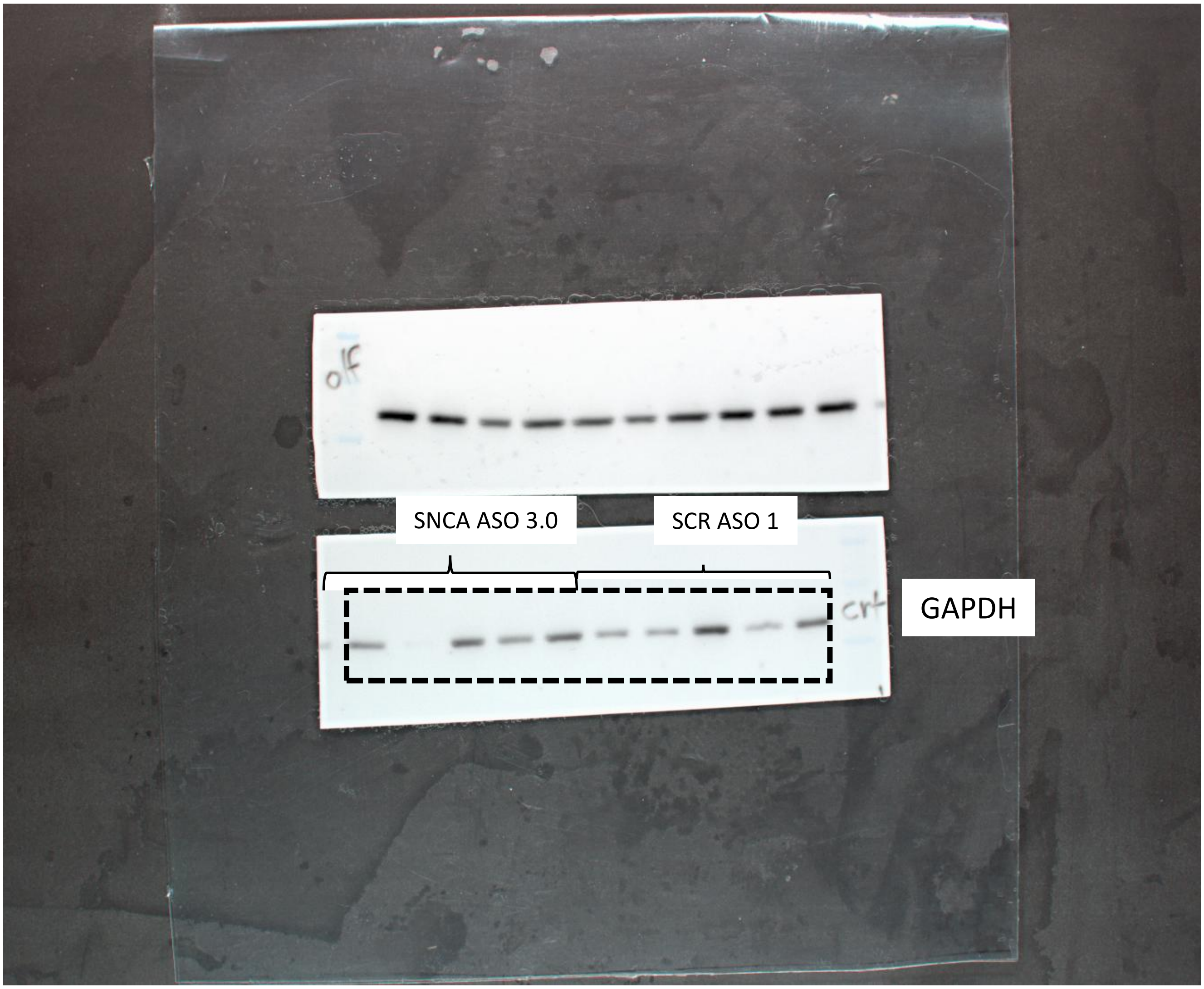

Figure S4

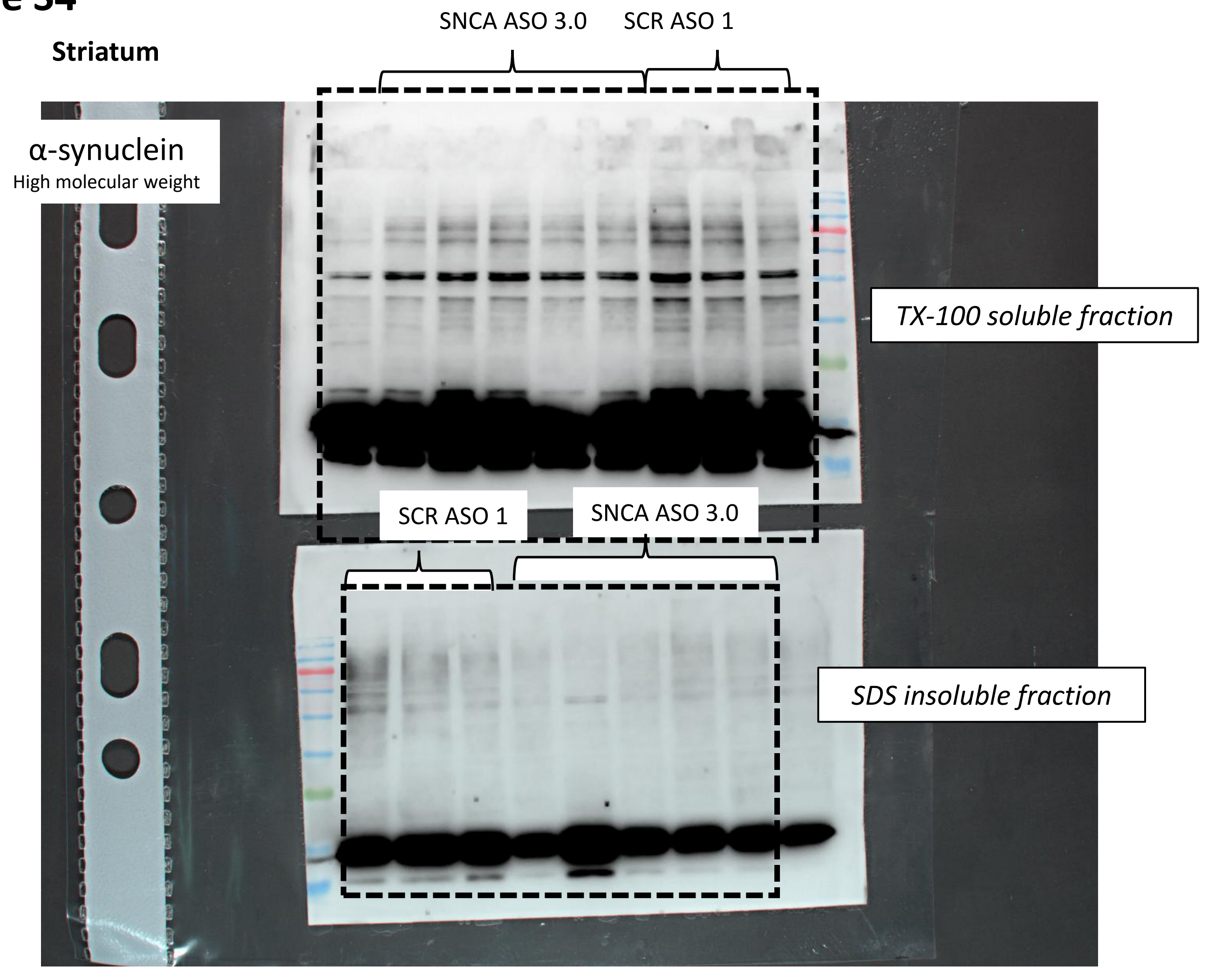
Striatum
SNCA ASO 3.0
SCR ASO 1
α-synuclein
High molecular weight
TX-100 soluble fraction
SCR ASO 1
SNCA ASO 3.0
SDS insoluble fraction

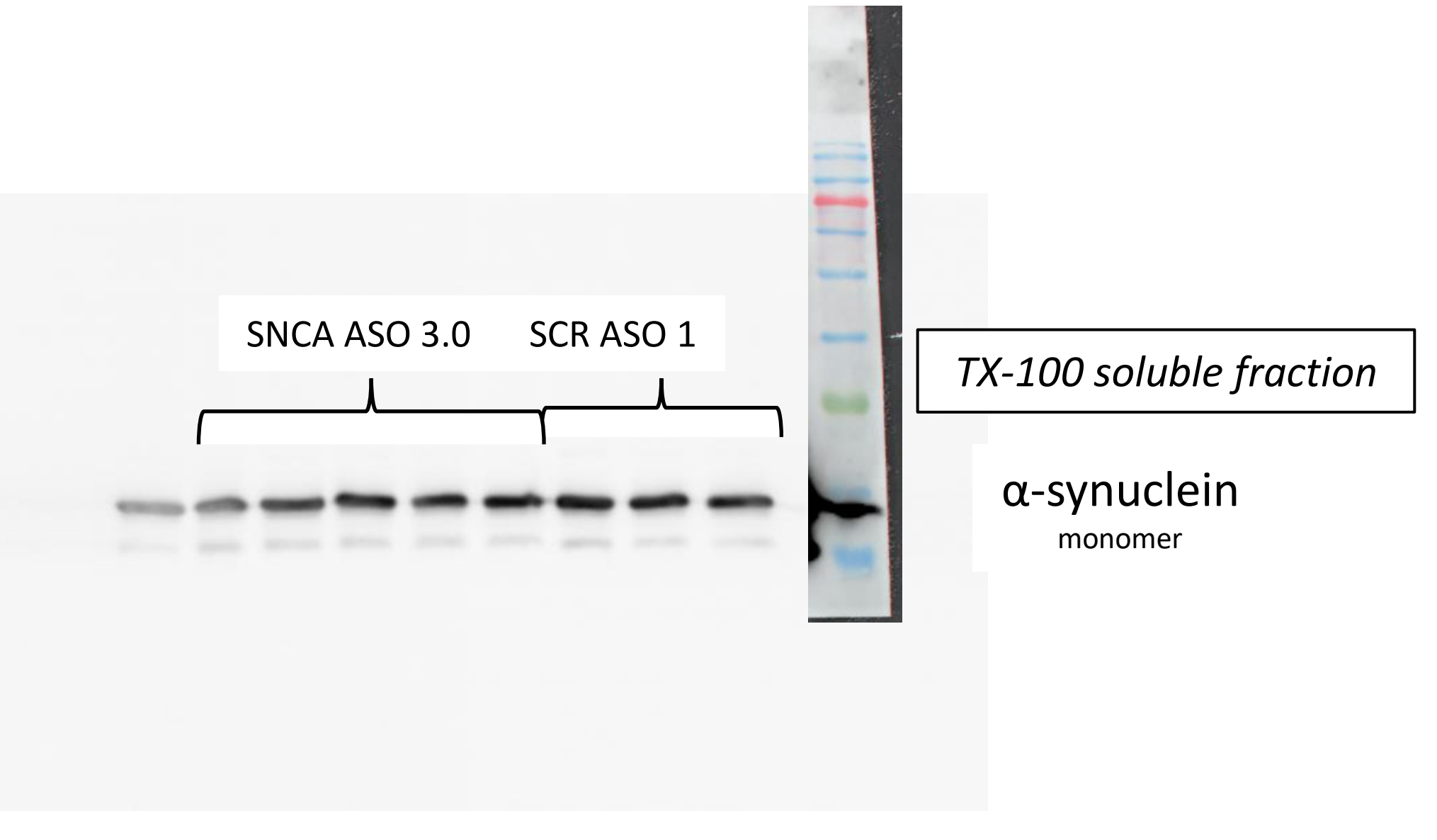
SNCA ASO 3.0
SCR ASO 1
TX-100 soluble fraction
α-synuclein
monomer

Figure S4

Striatum

GAPDH

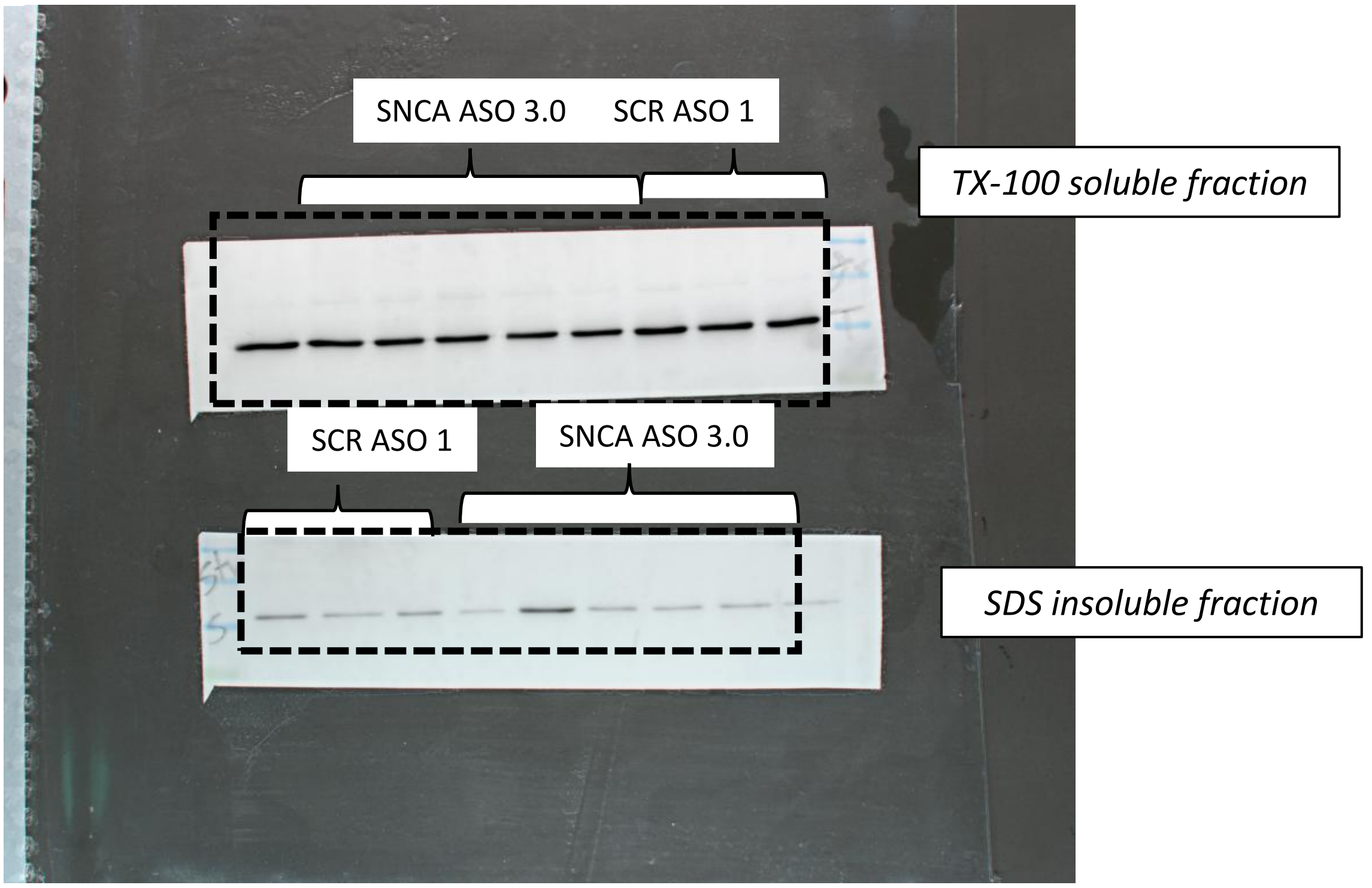

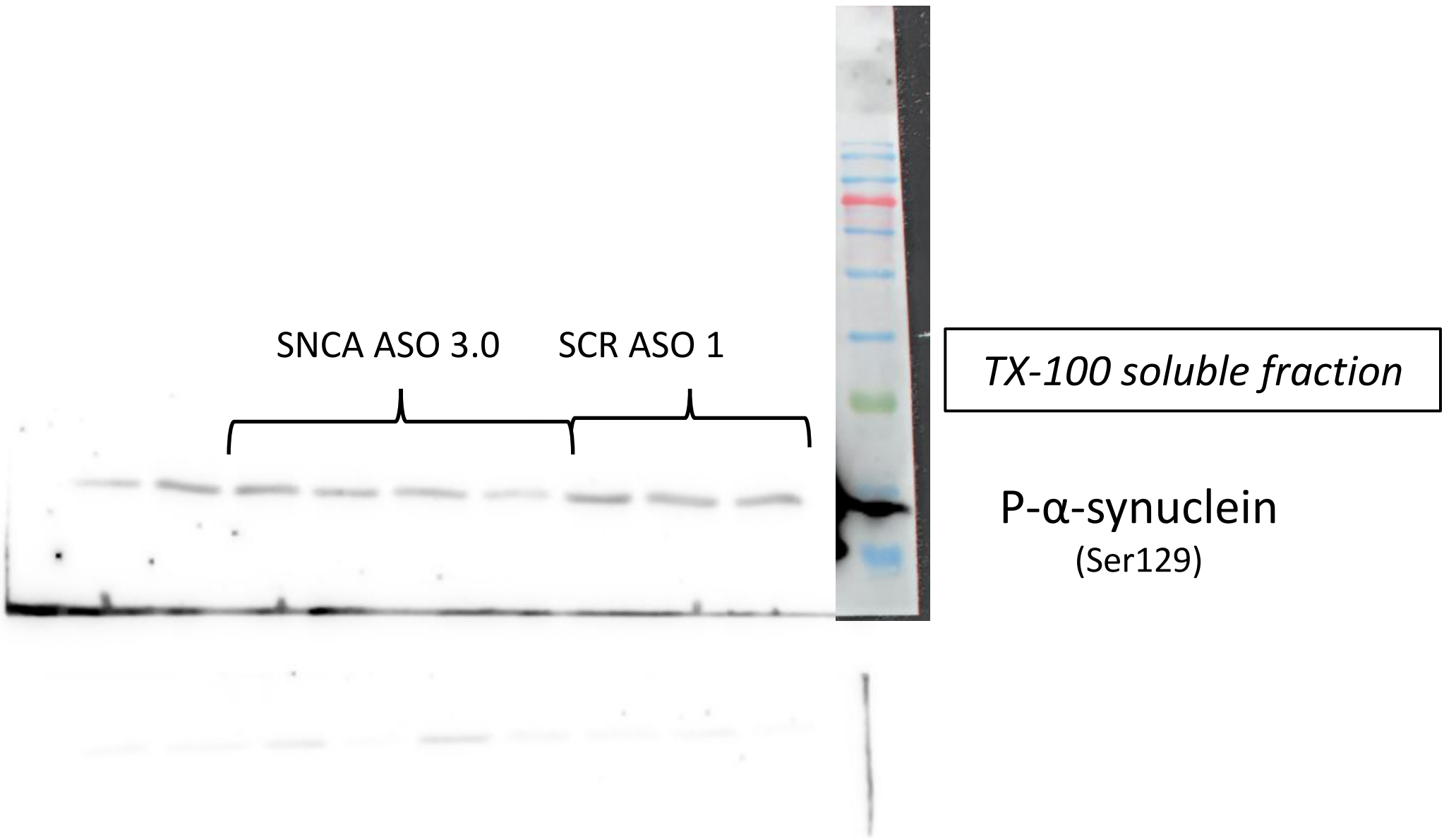

Figure S4

Striatum

SDS insoluble fraction

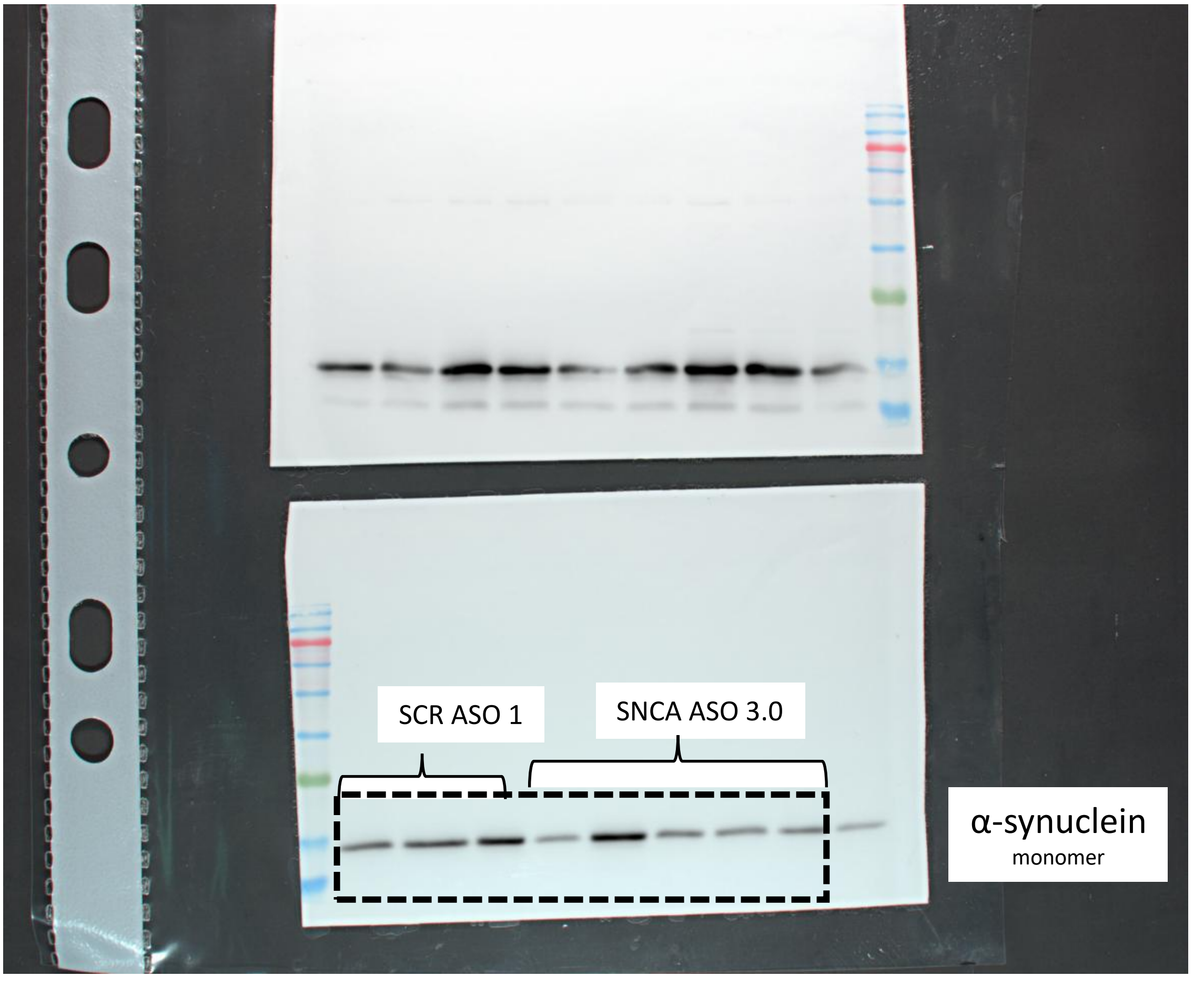

Figure S4

Hippocampus

Triton X-100 soluble fraction

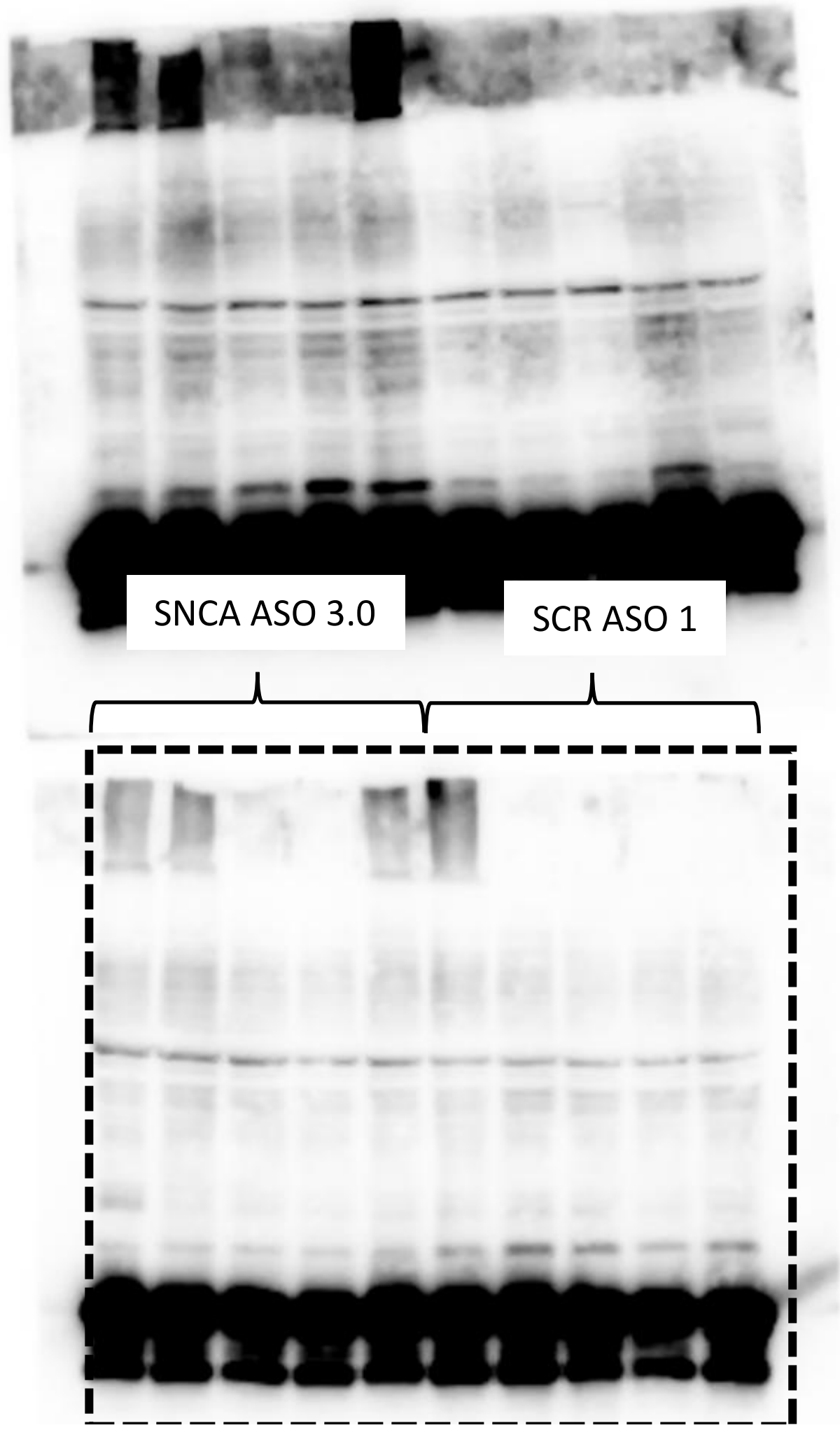

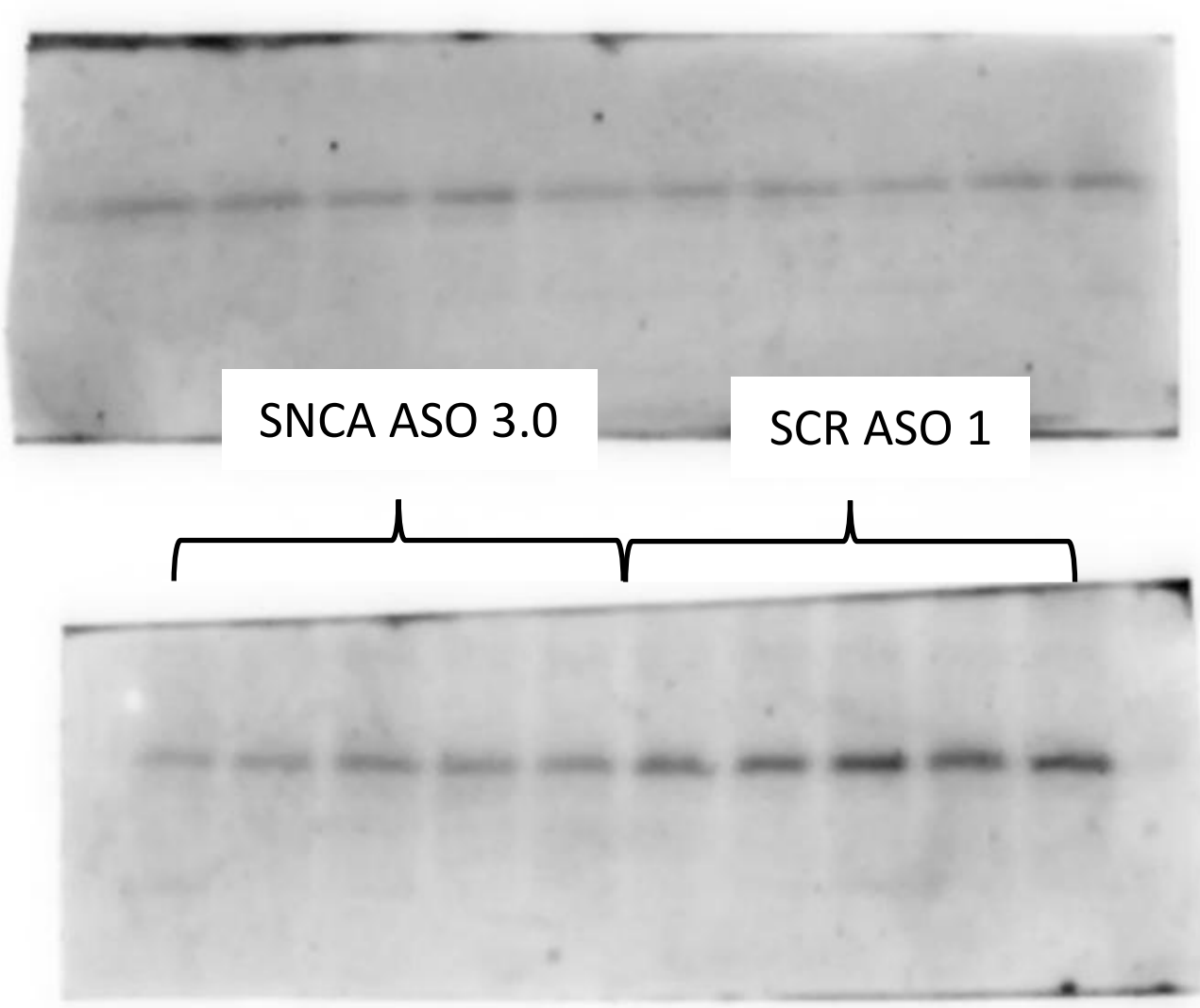

Figure S4

Hippocampus
Triton X-100 soluble fraction

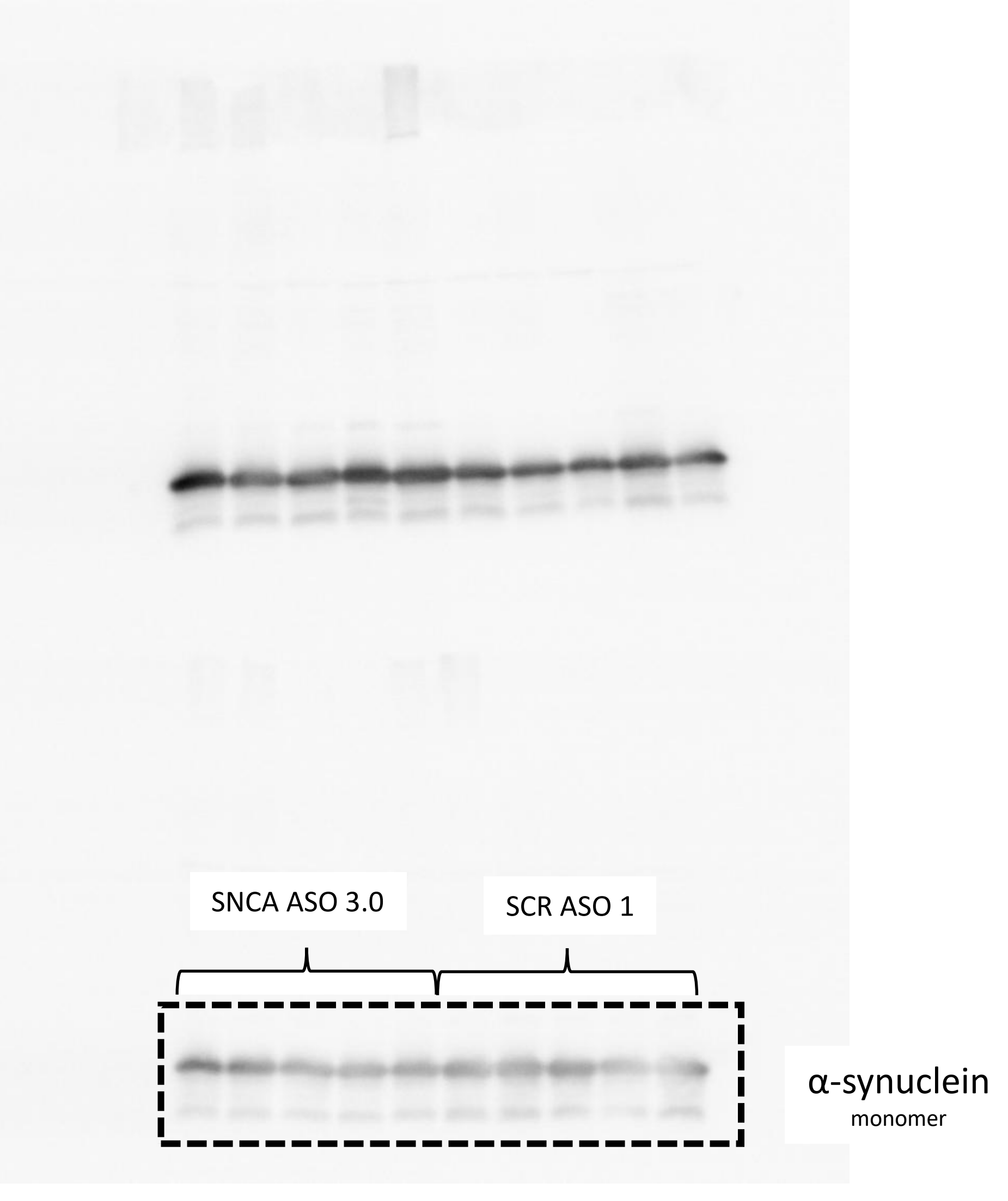

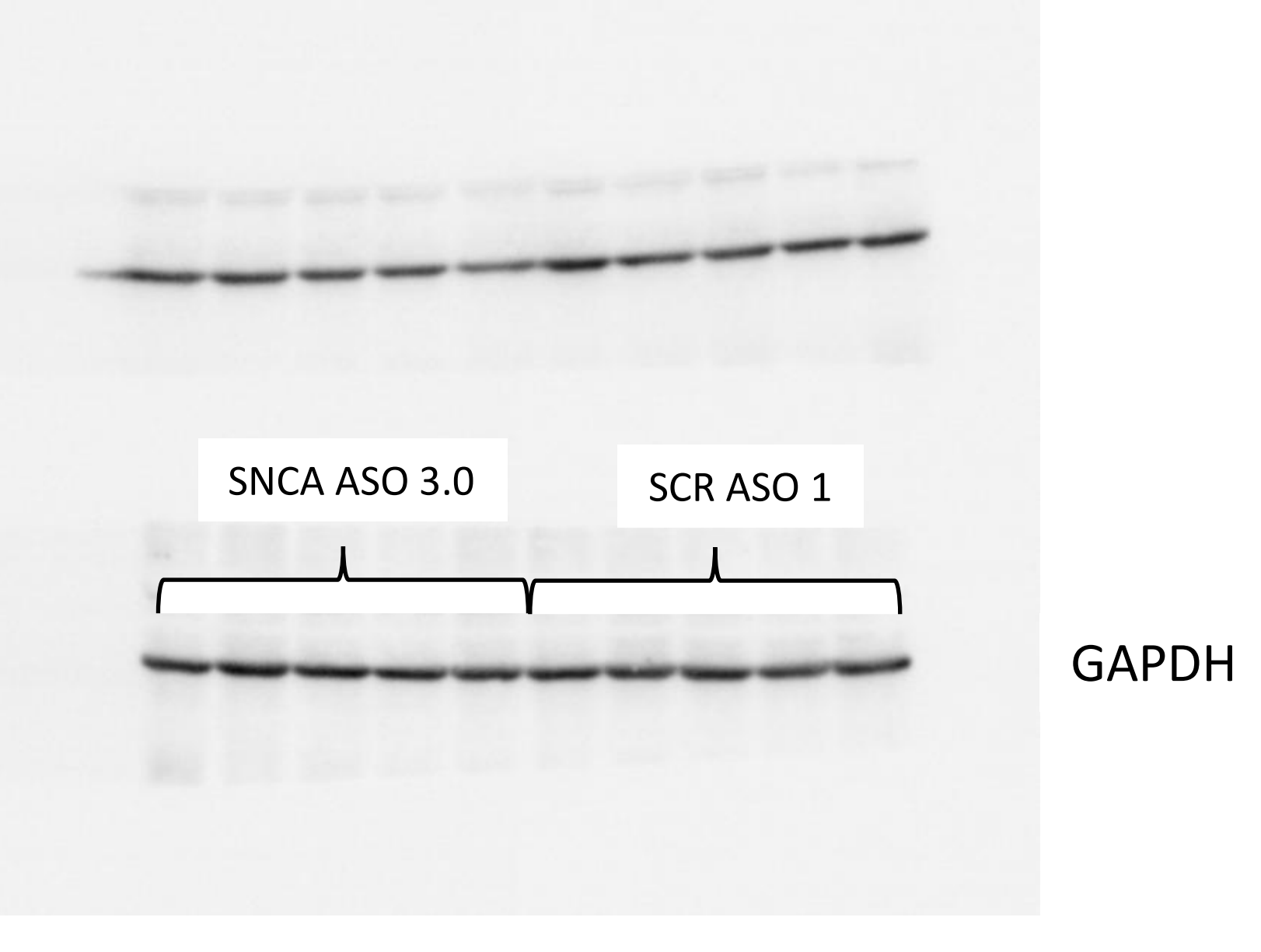

Figure S4

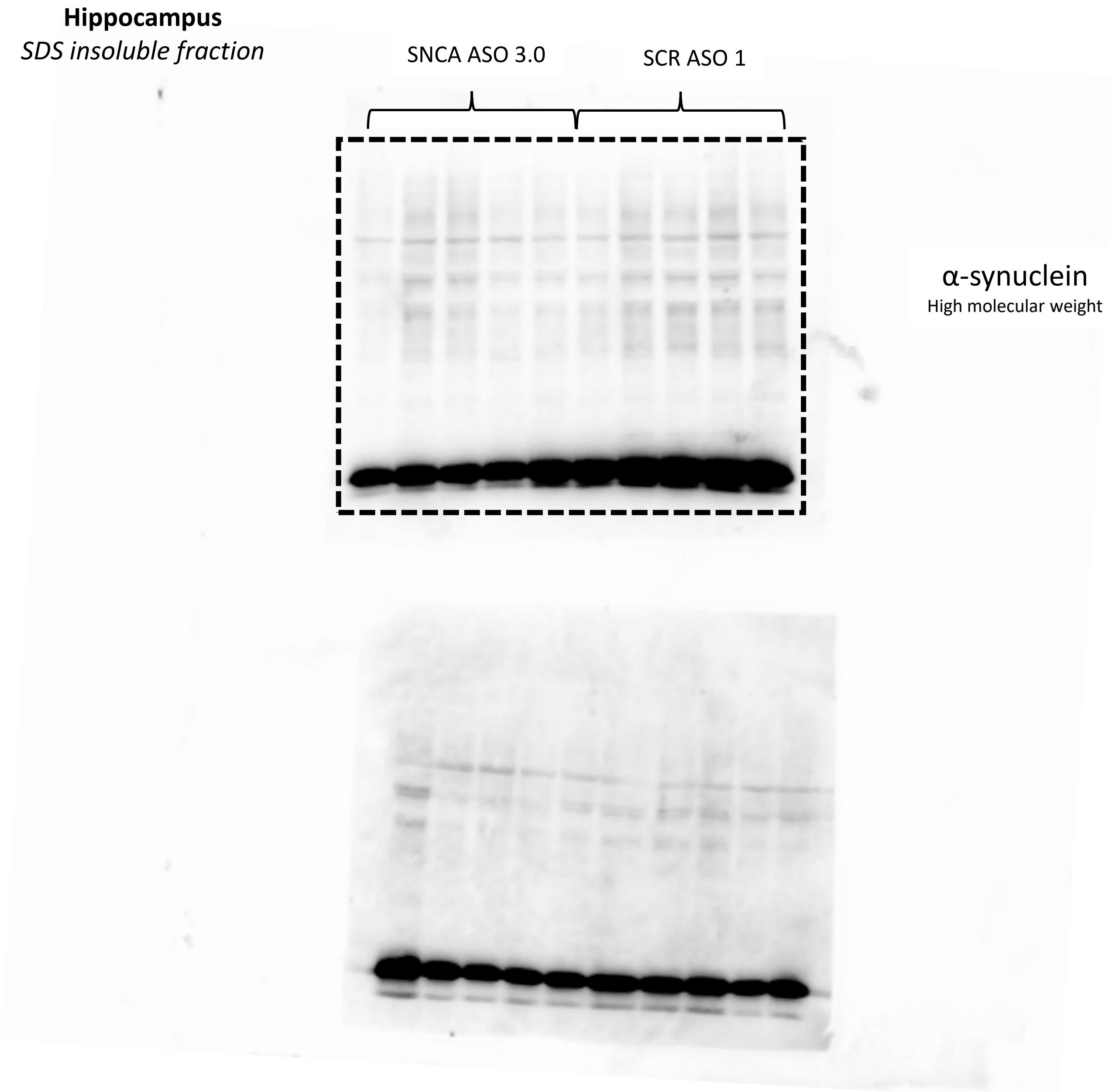
Hippocampus
SDS insoluble fraction
SNCA ASO 3.0
SCR ASO 1
α-synuclein
High molecular weight

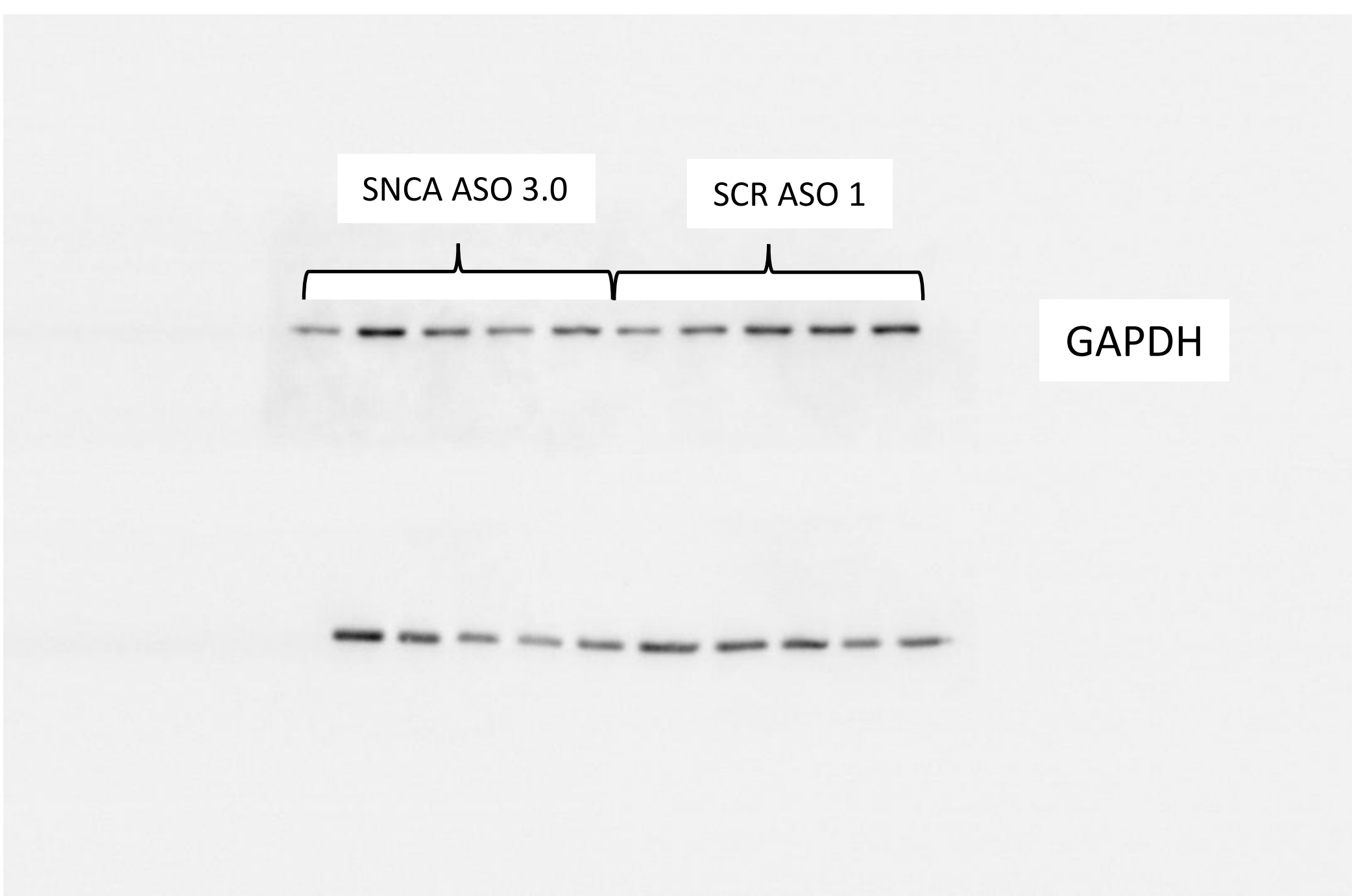
SNCA ASO 3.0
SCR ASO 1
GAPDH

Figure S4

Hippocampus
SDS insoluble fraction

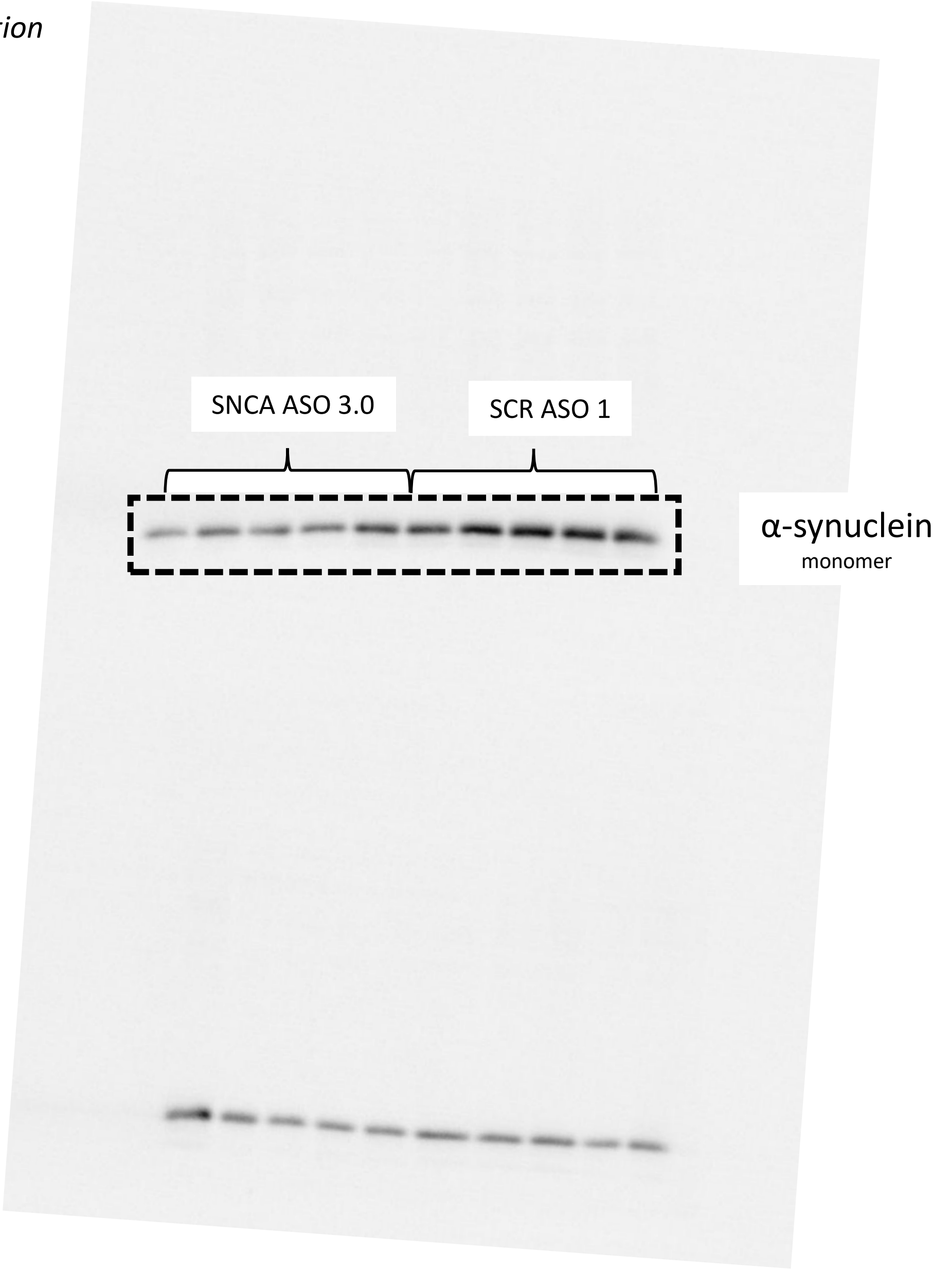

Figure S4

Dorsal Midbrain

TX-100 soluble fraction

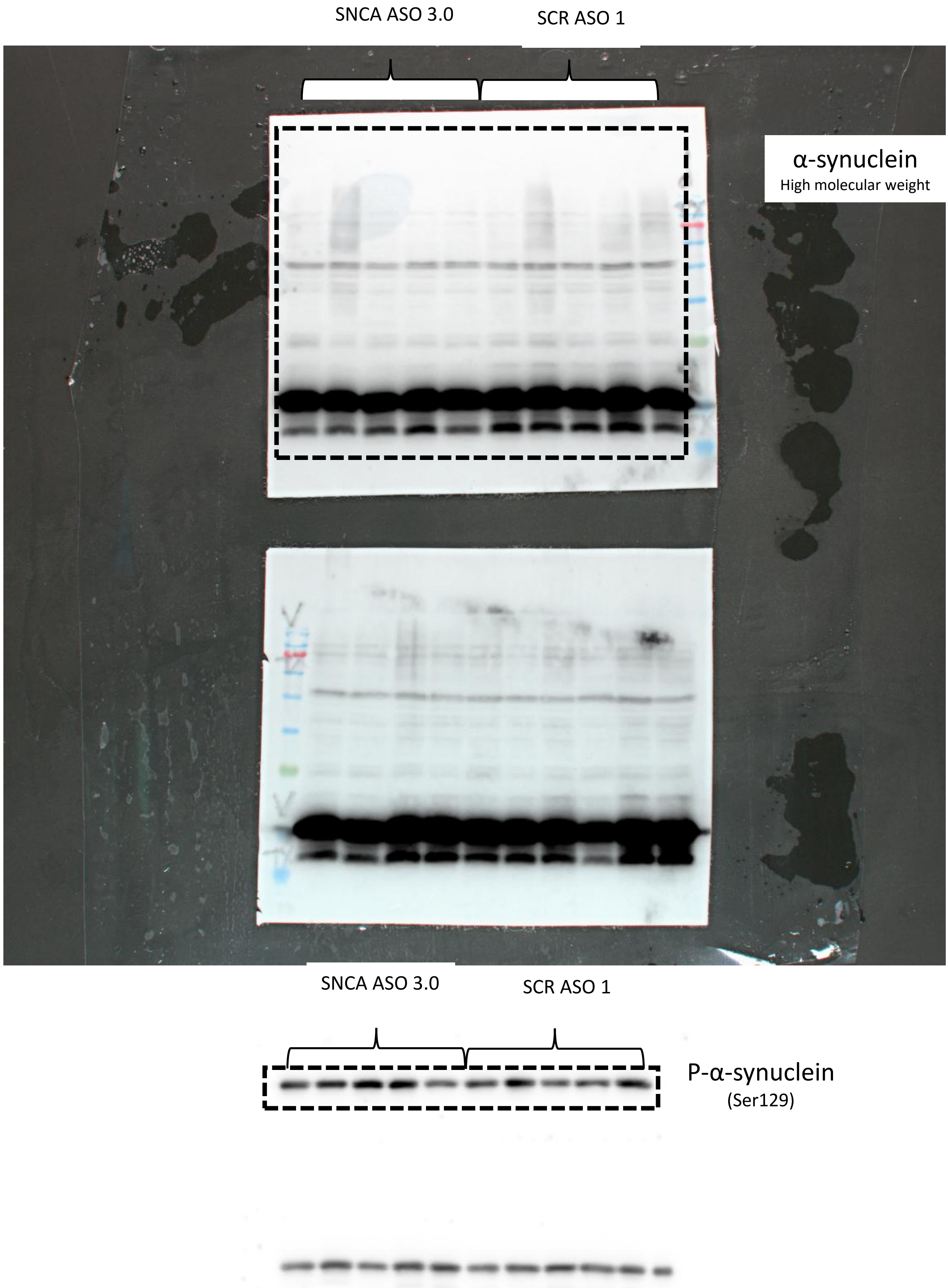

Figure S4

Dorsal Midbrain

TX-100 soluble fraction

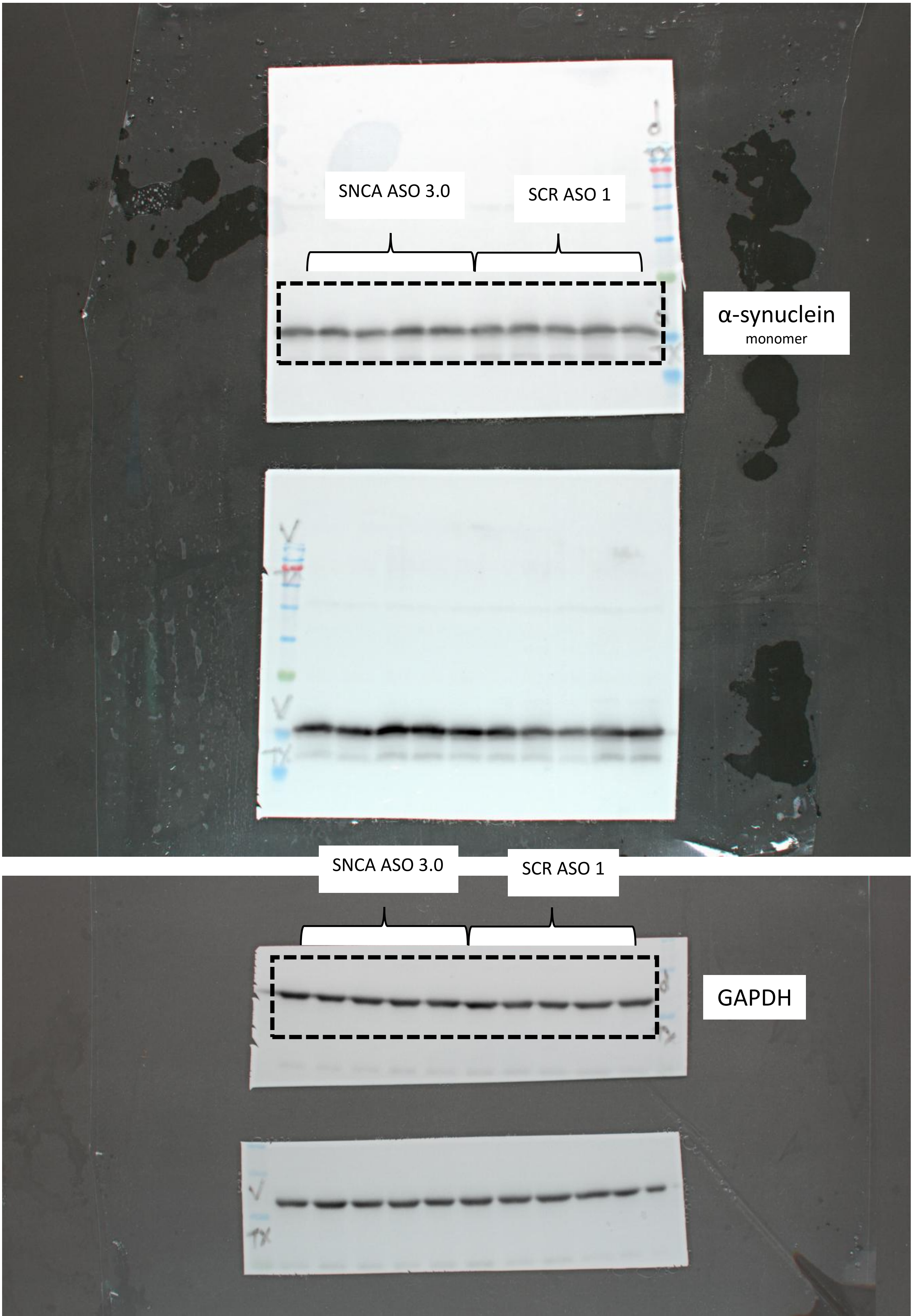

Figure S4

Dorsal Midbrain
SDS insoluble fraction

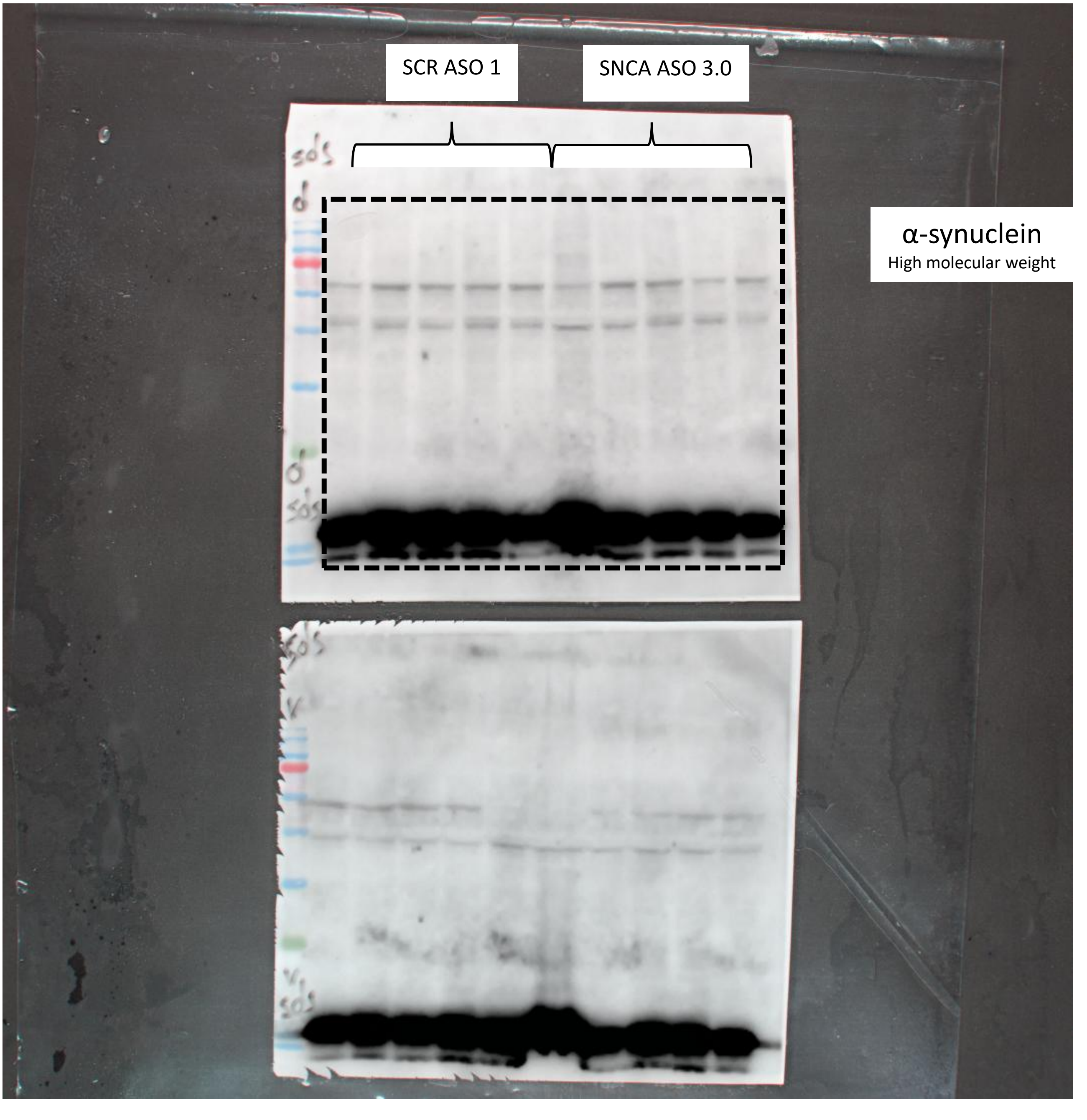

Figure S4

Dorsal Midbrain

SDS insoluble fraction

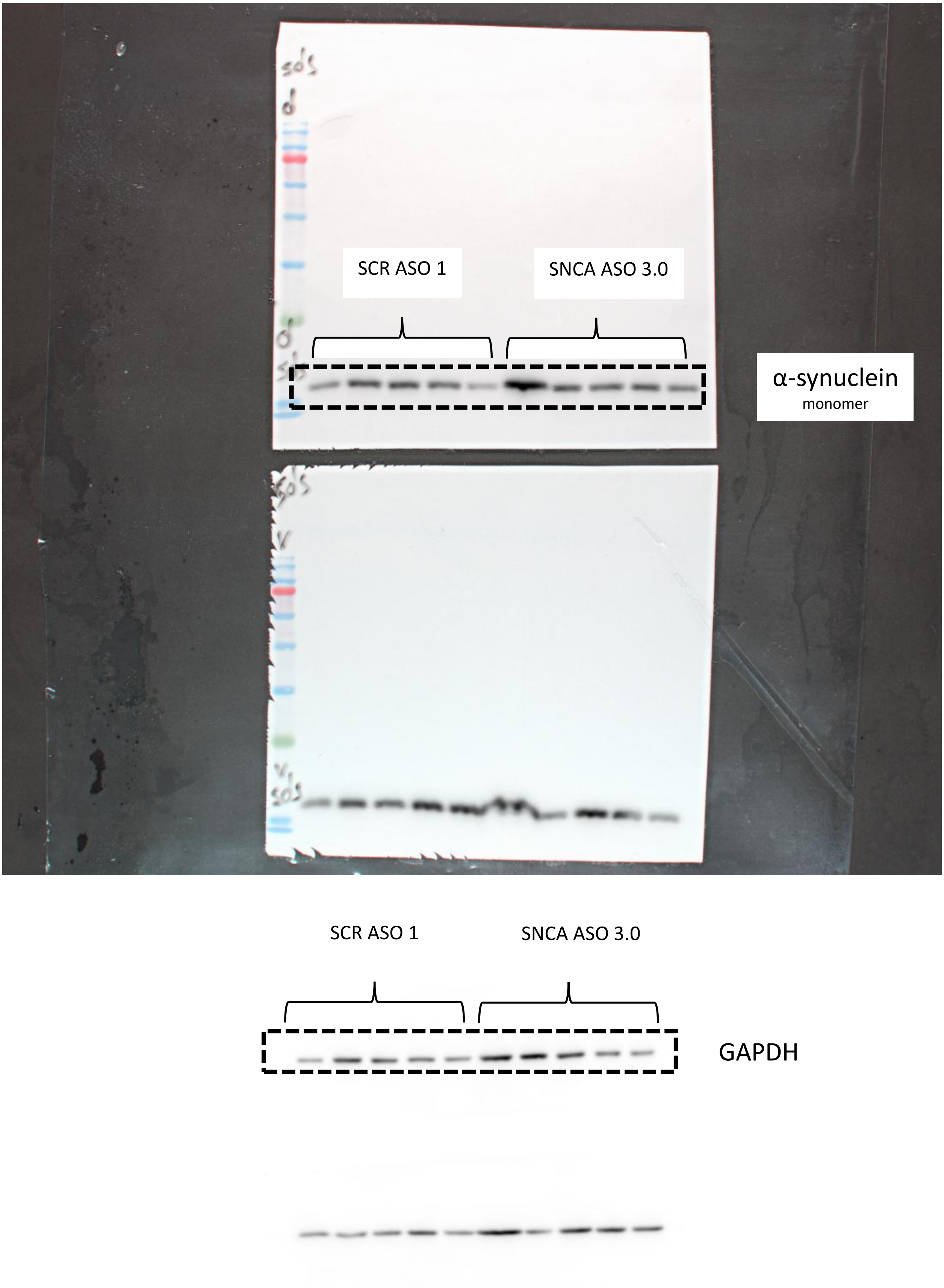

Figure S4

Ventral Midbrain

TX-100 soluble fraction

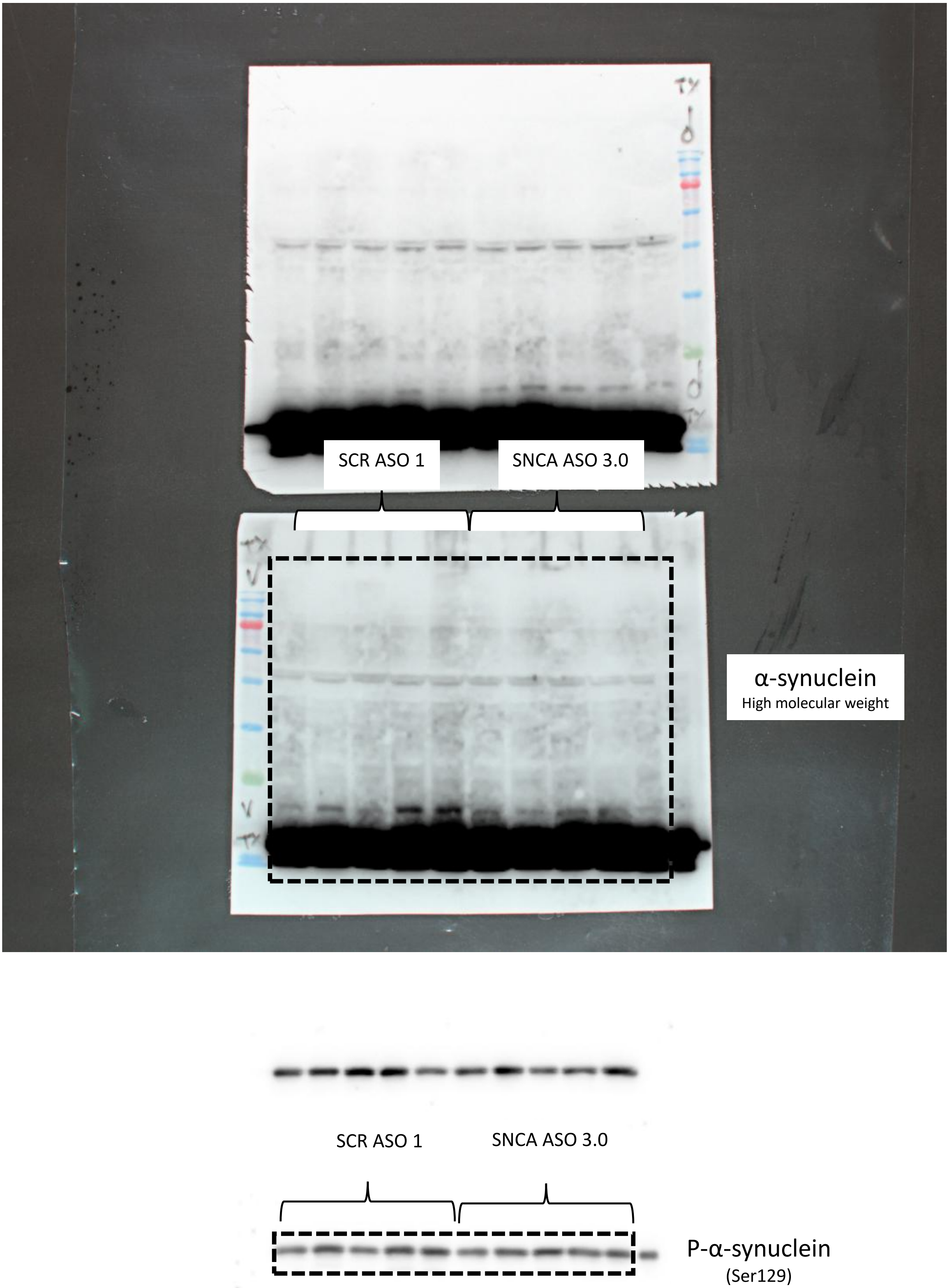

Figure S4

Ventral Midbrain

TX-100 soluble fraction

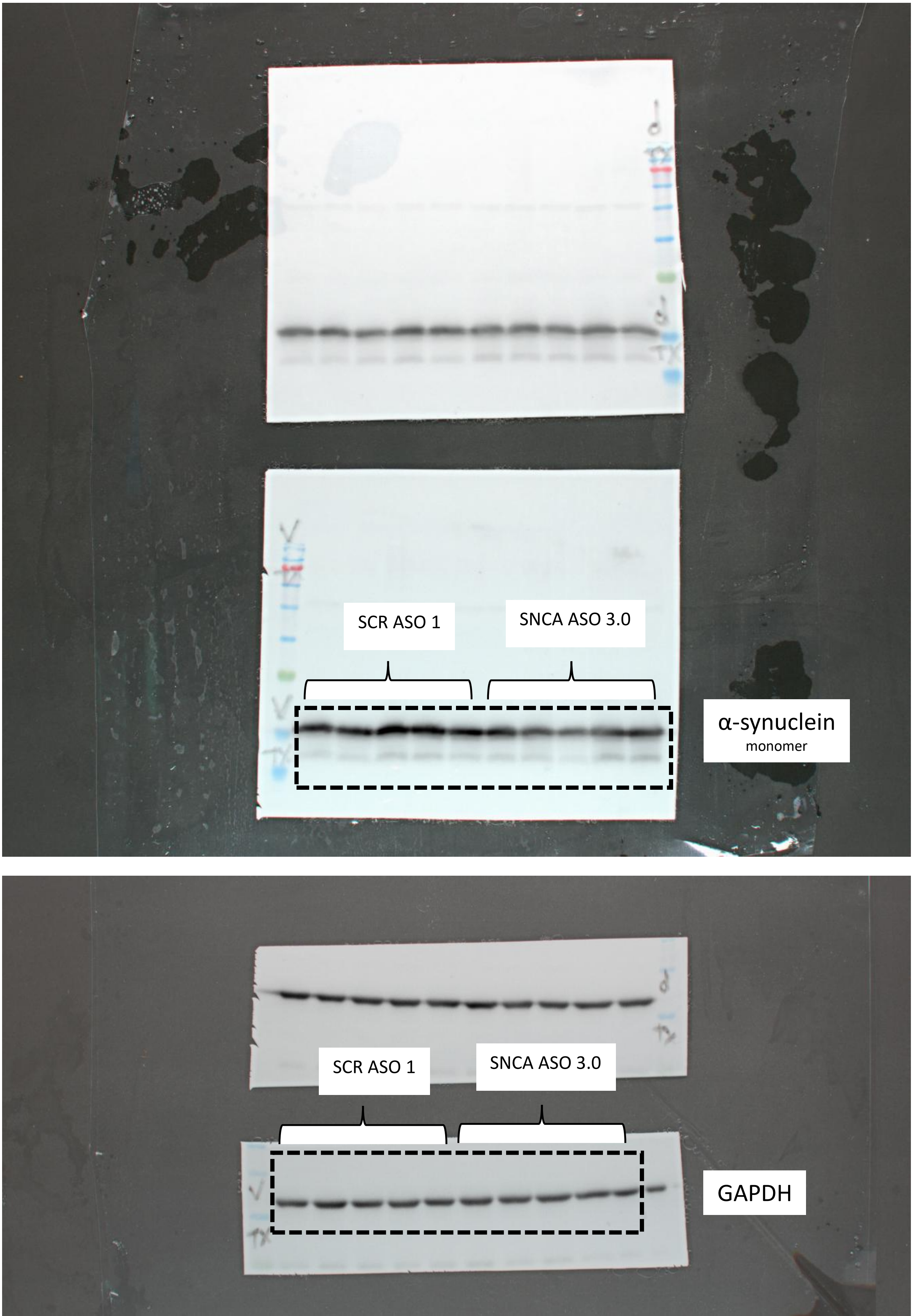

Figure S4

Ventral Midbrain

SDS insoluble fraction

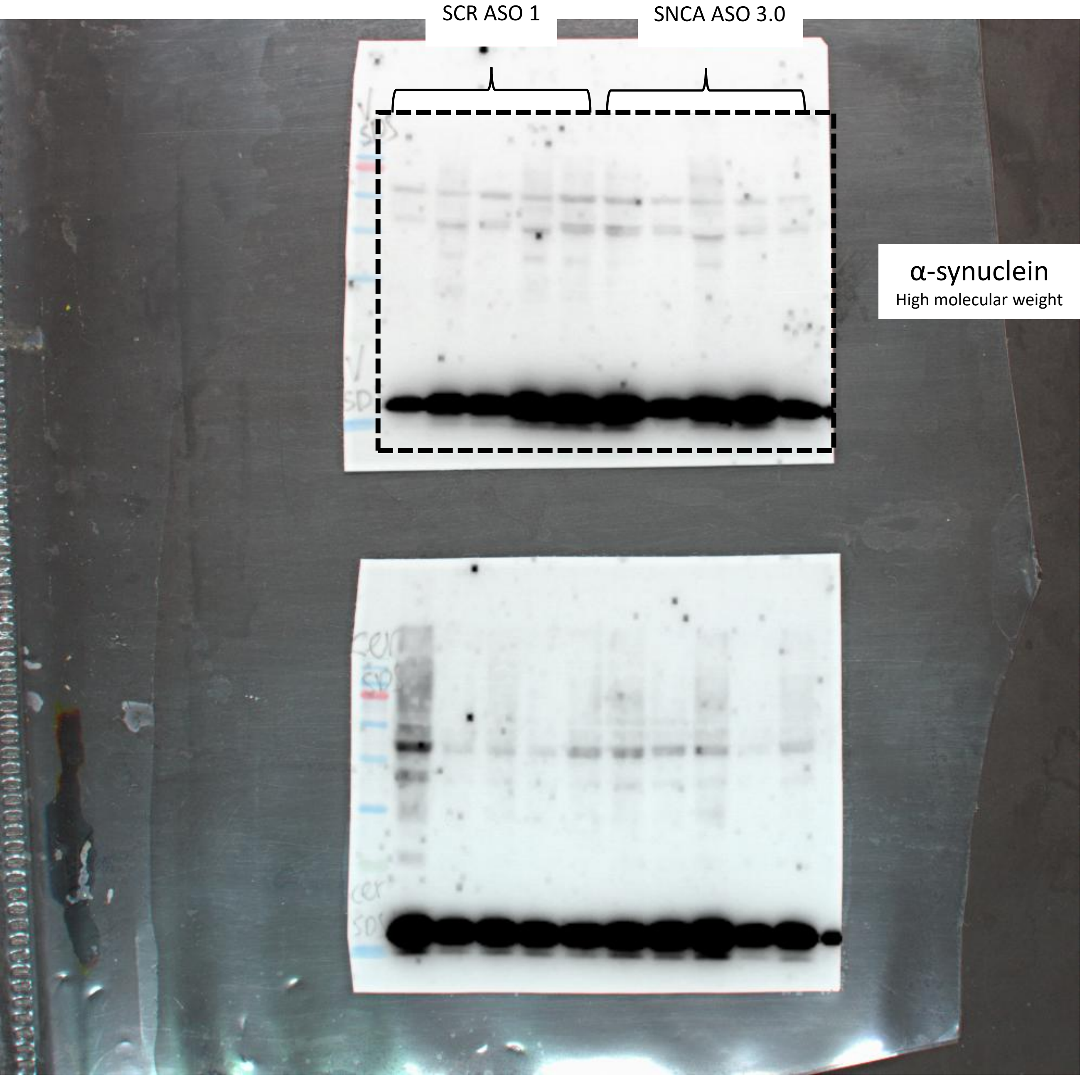

Figure S4

Ventral Midbrain

SDS insoluble fraction

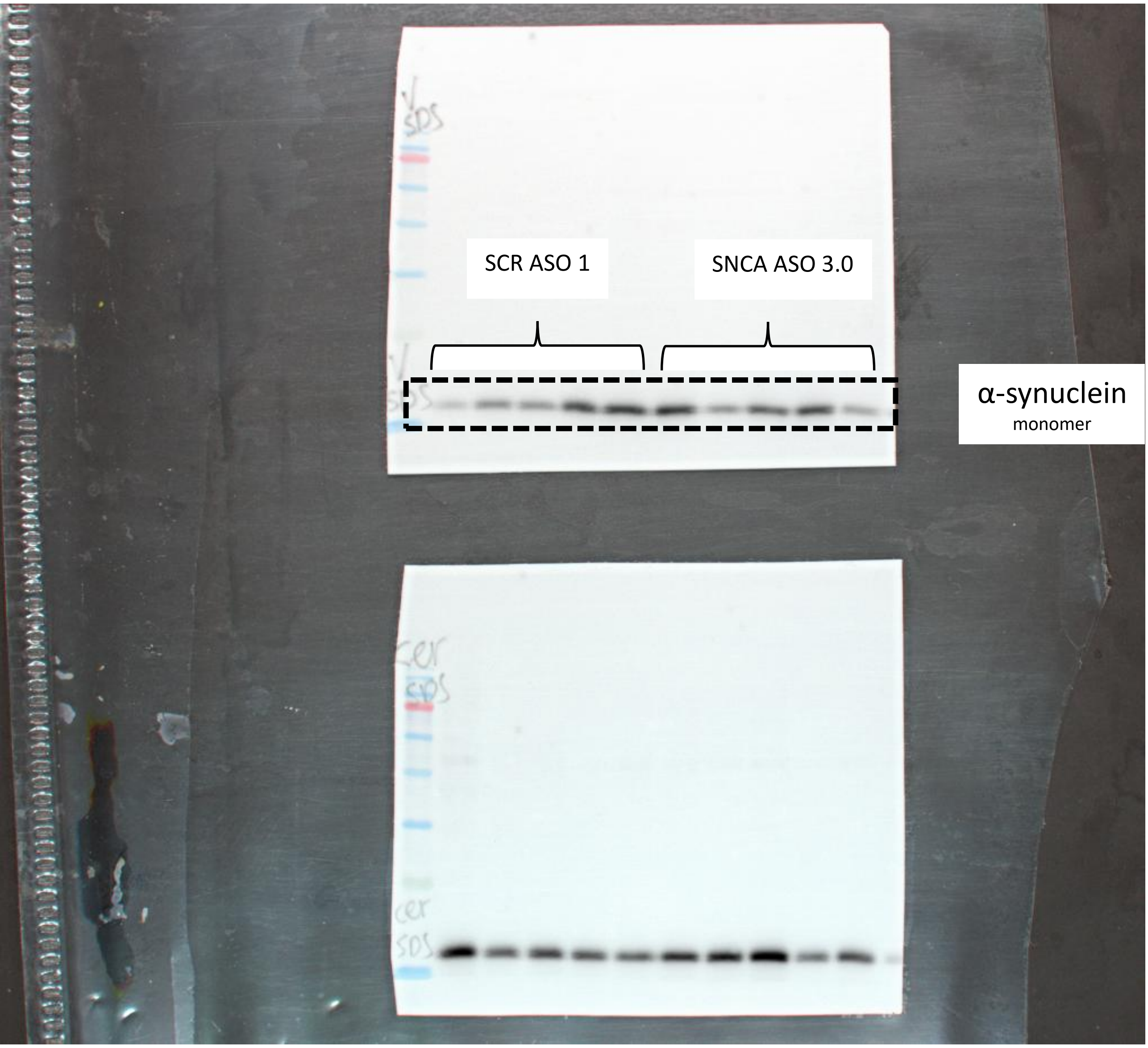

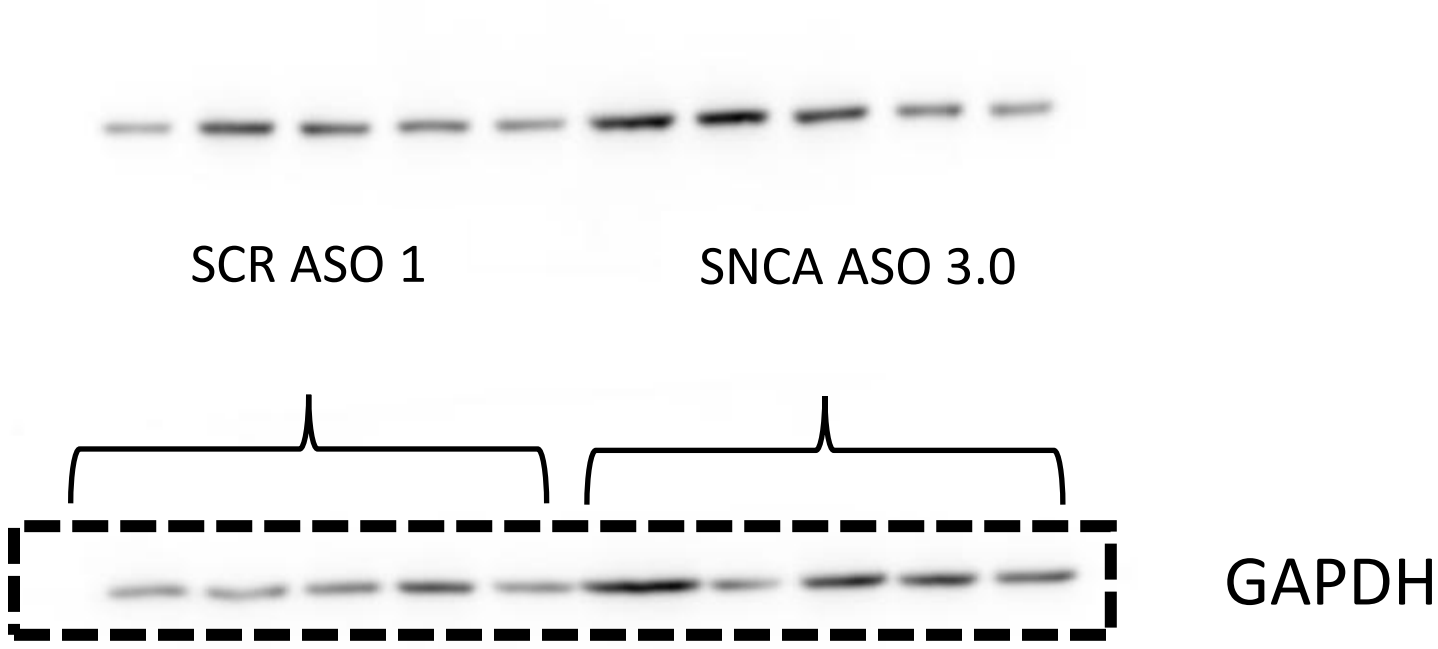

Figure S4

Cerebellum
TX-100 soluble fraction

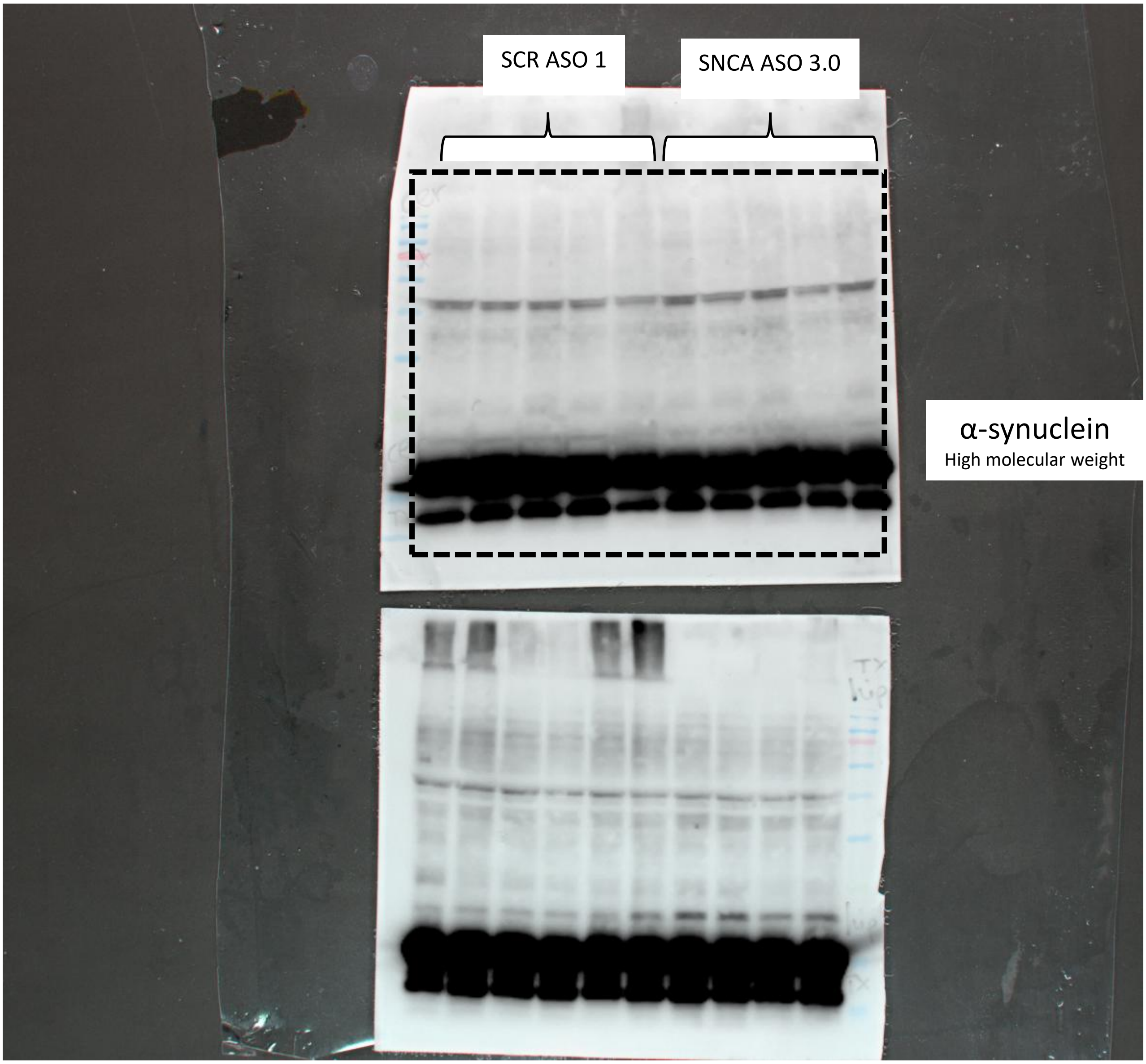

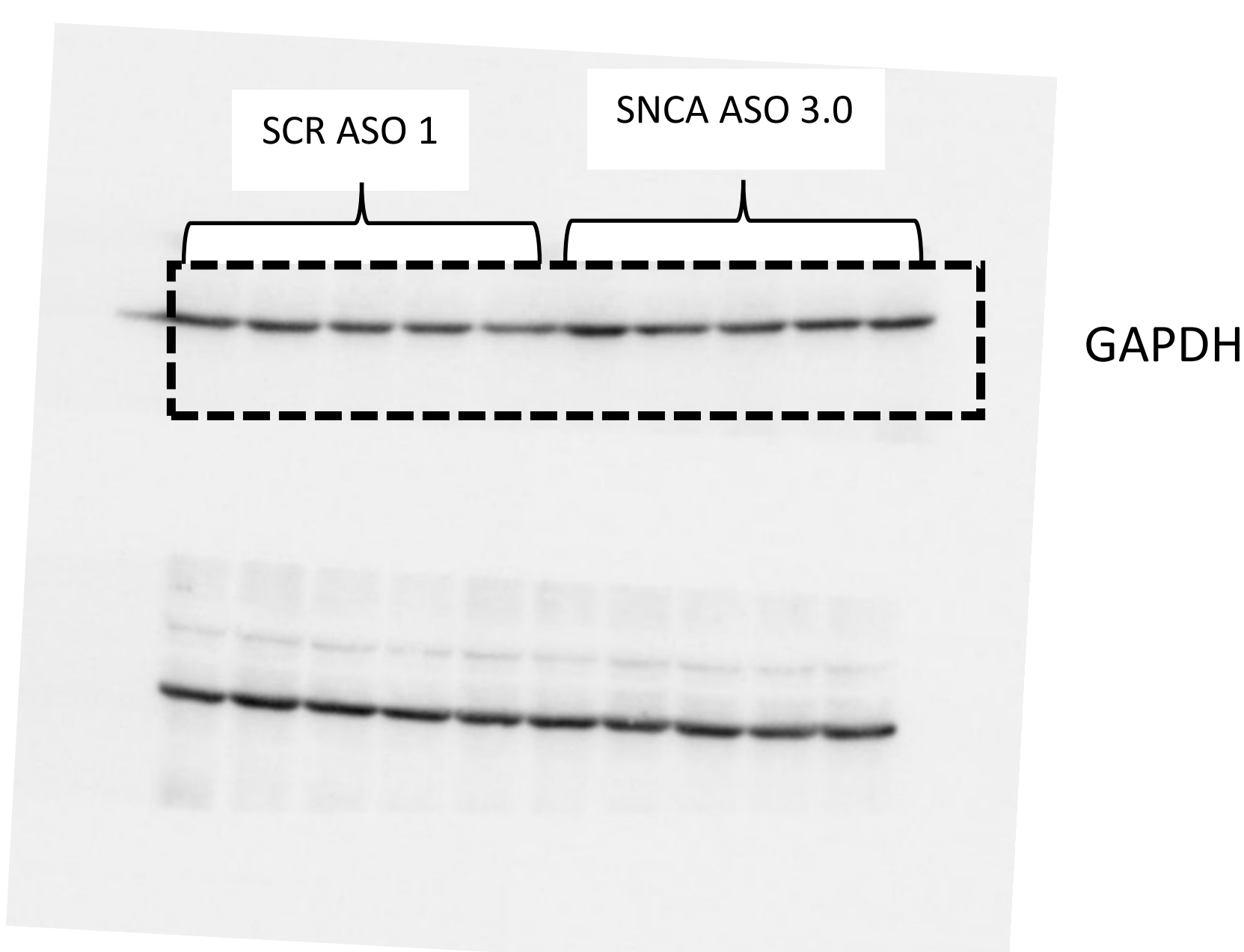

Figure S4

Cerebellum
TX-100 soluble fraction

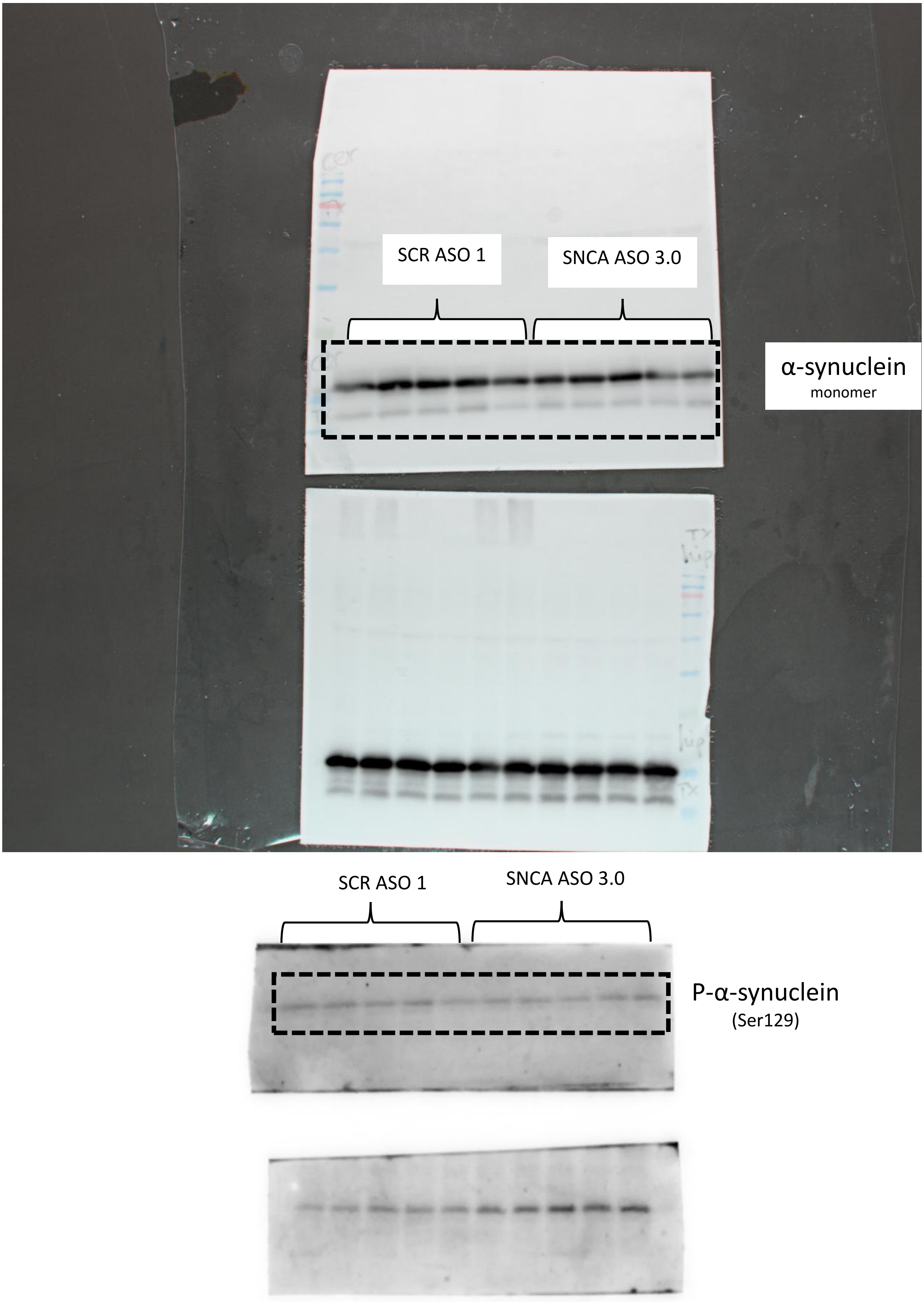

Figure S4

Cerebellum

SDS insoluble fraction

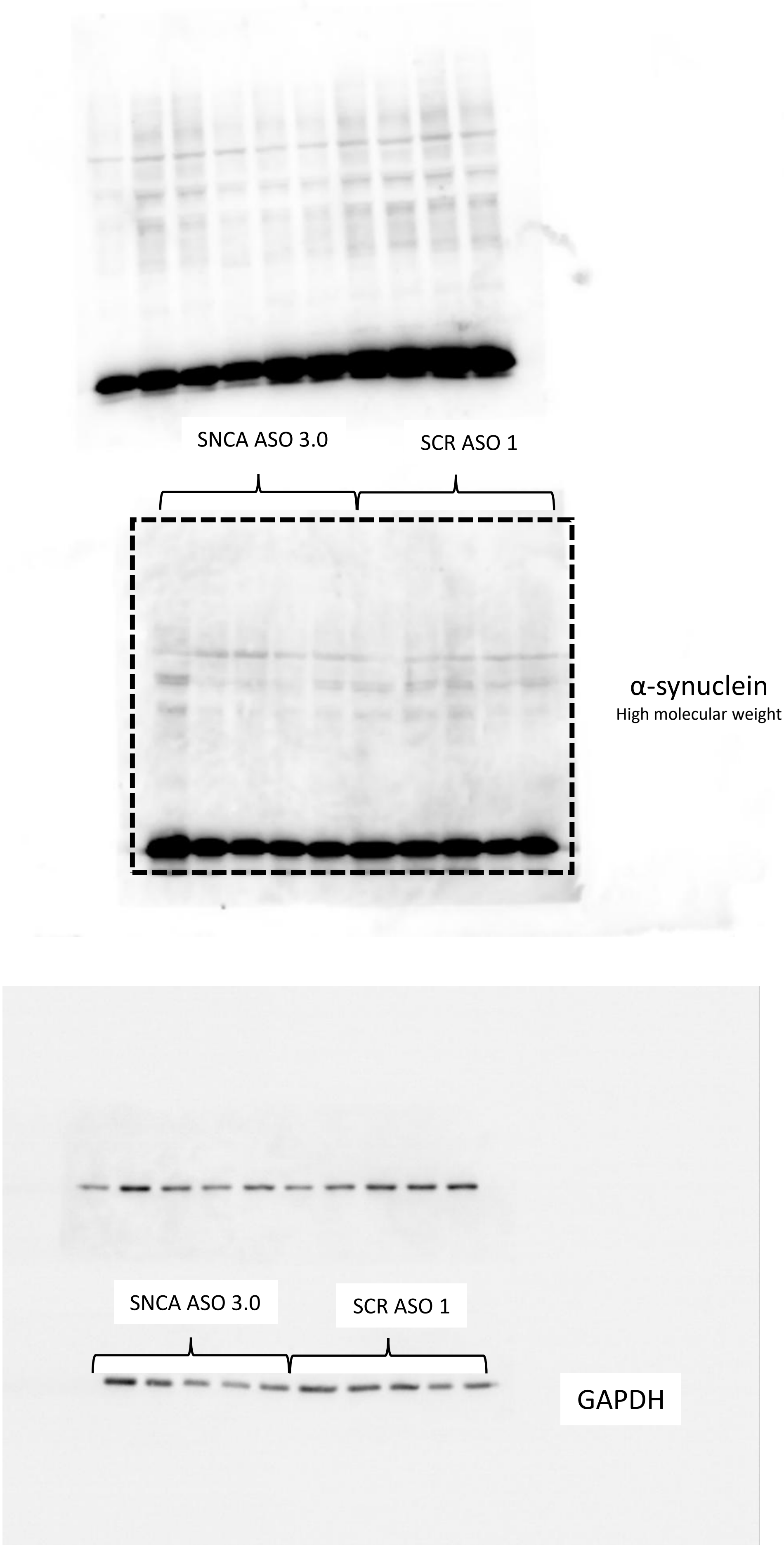

Figure S4

Cerebellum
SDS insoluble fraction

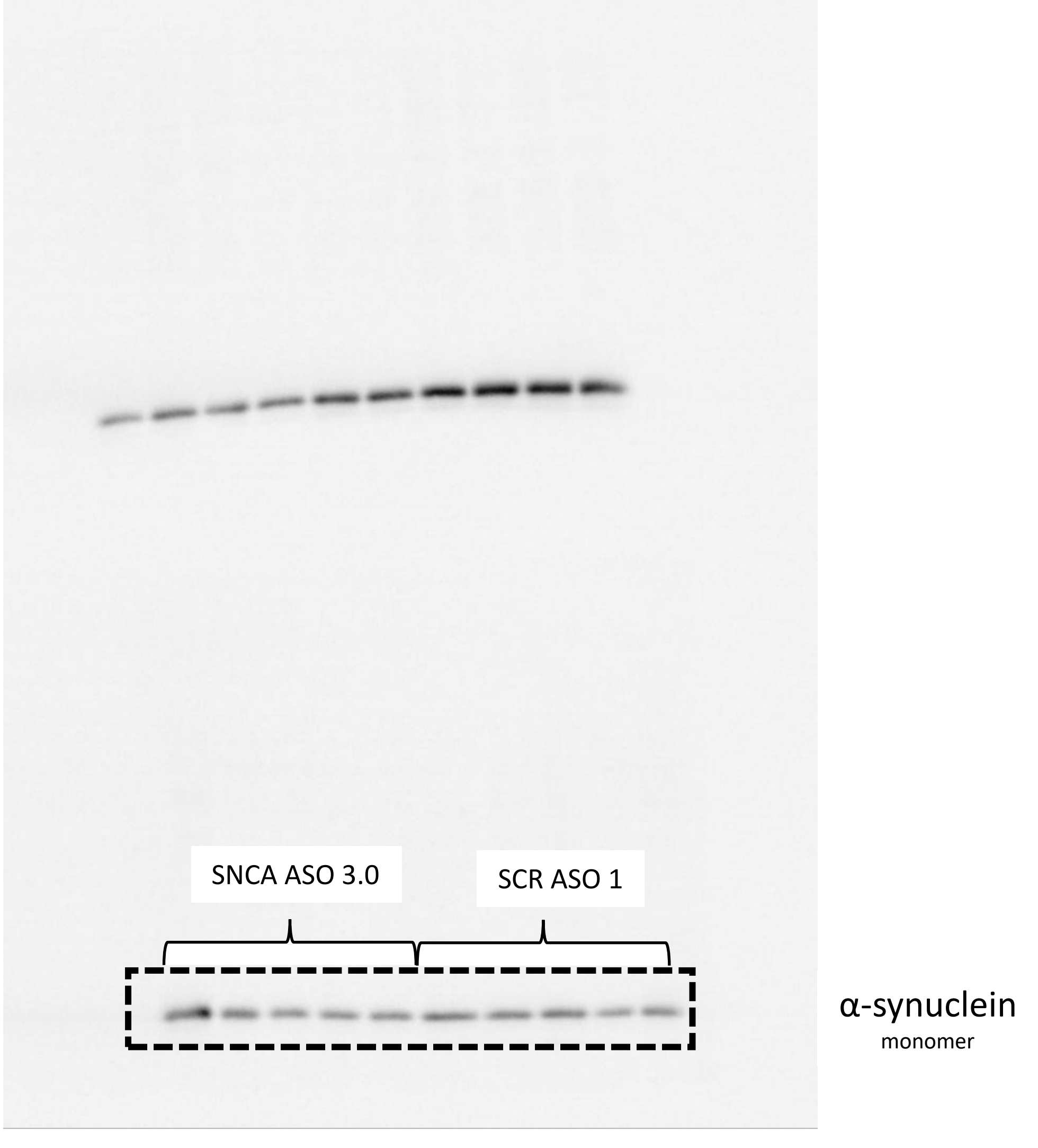

Figure S5

Striatum

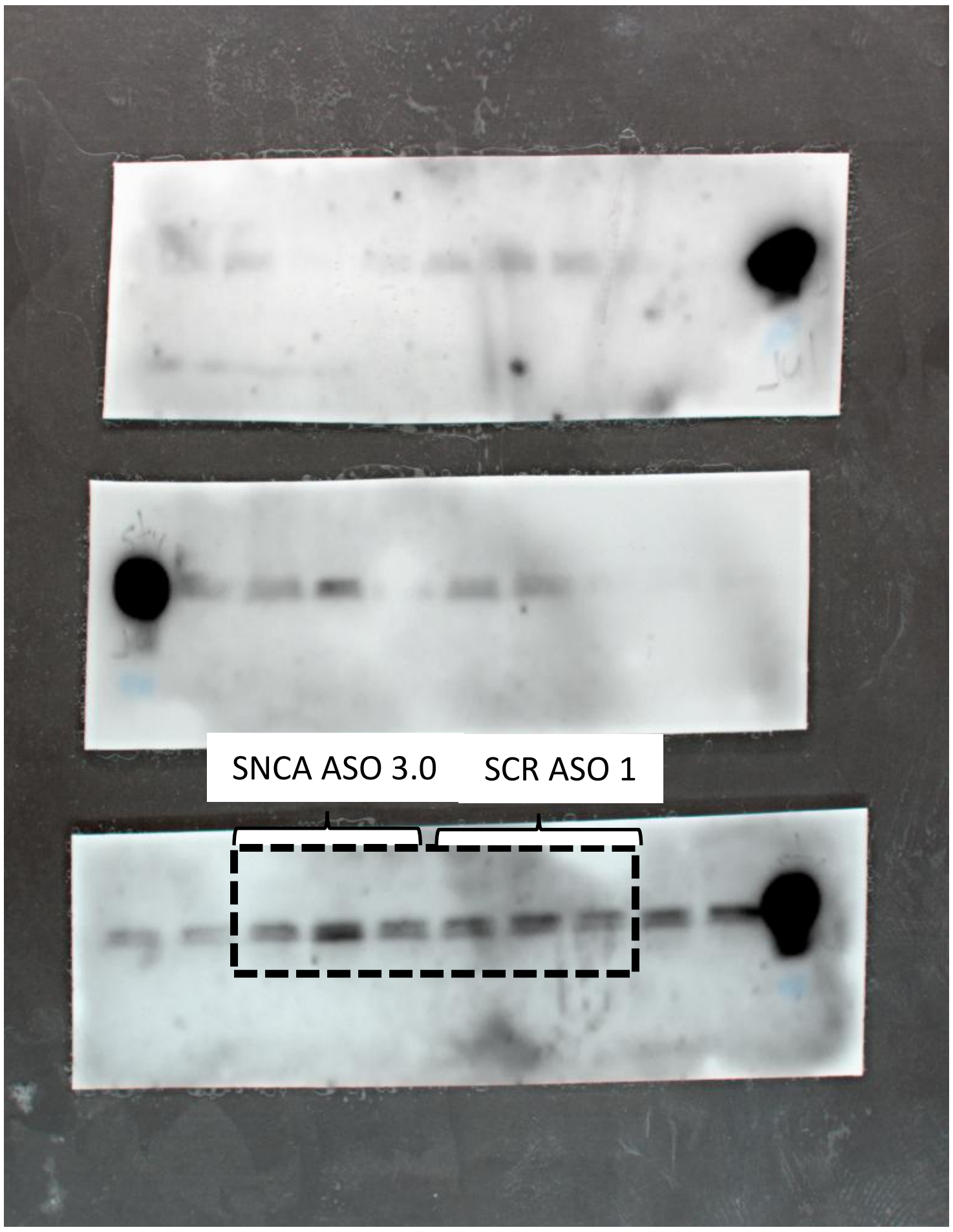

IBA1

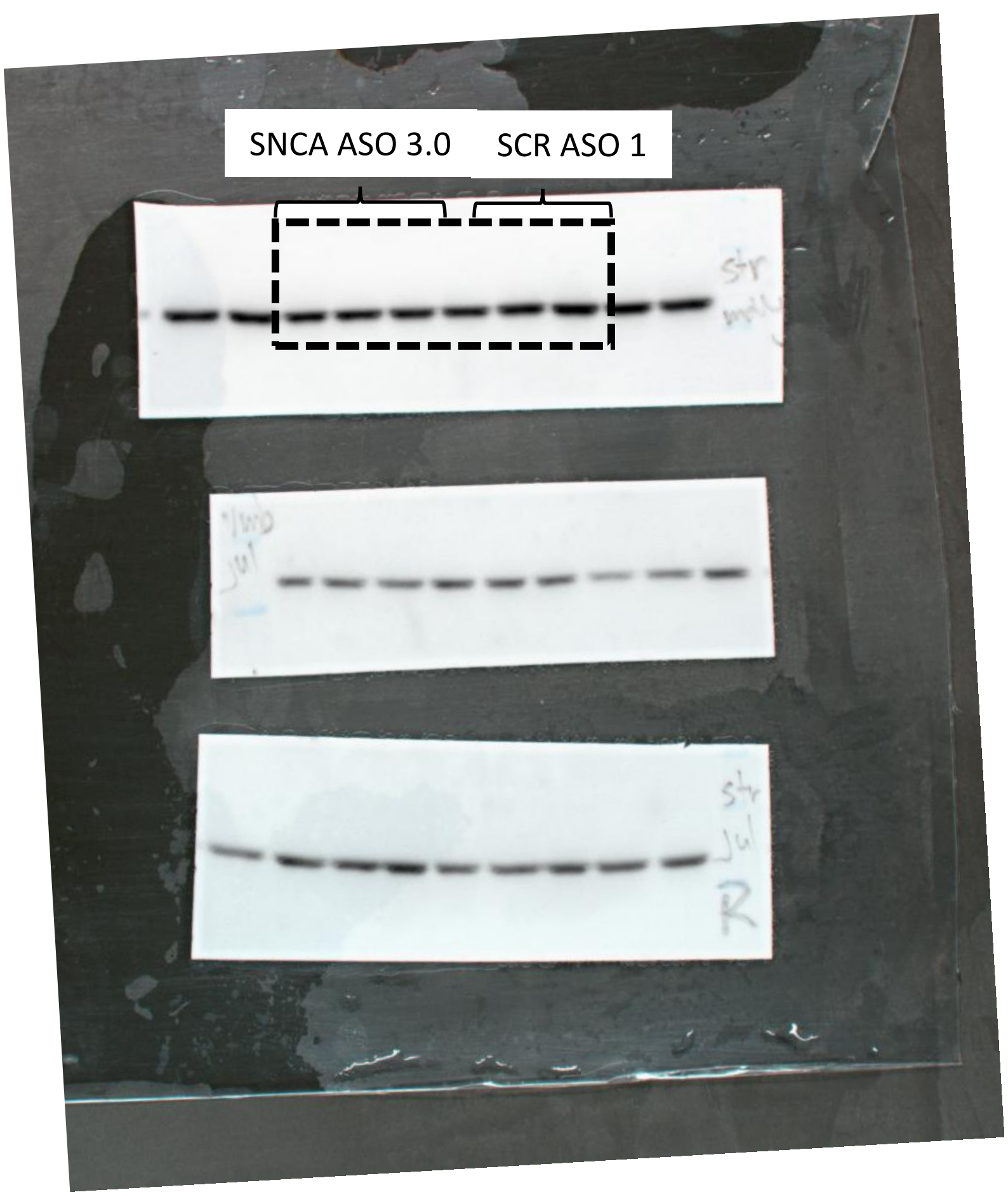

GAPDH

Figure S5

Striatum

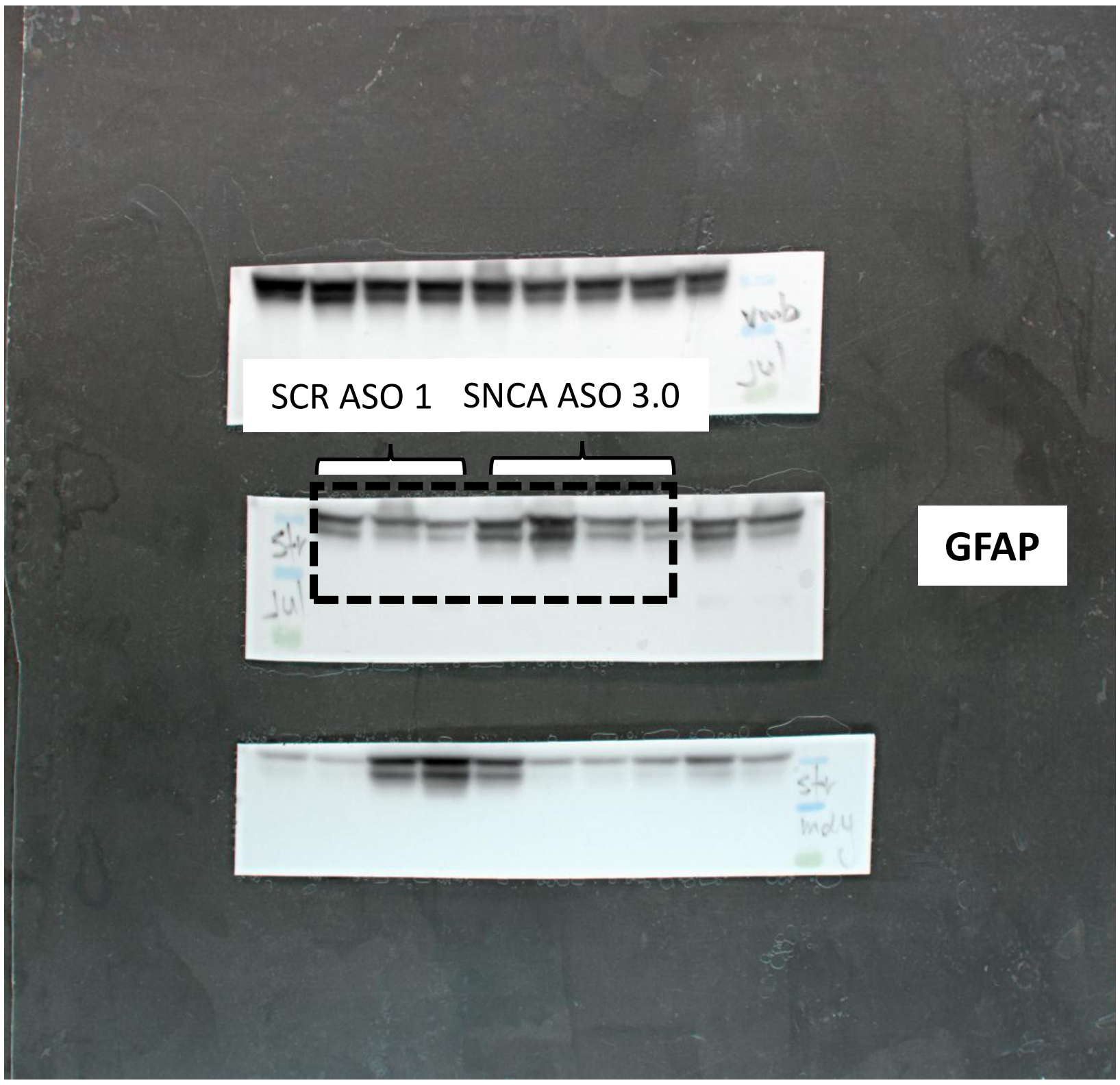

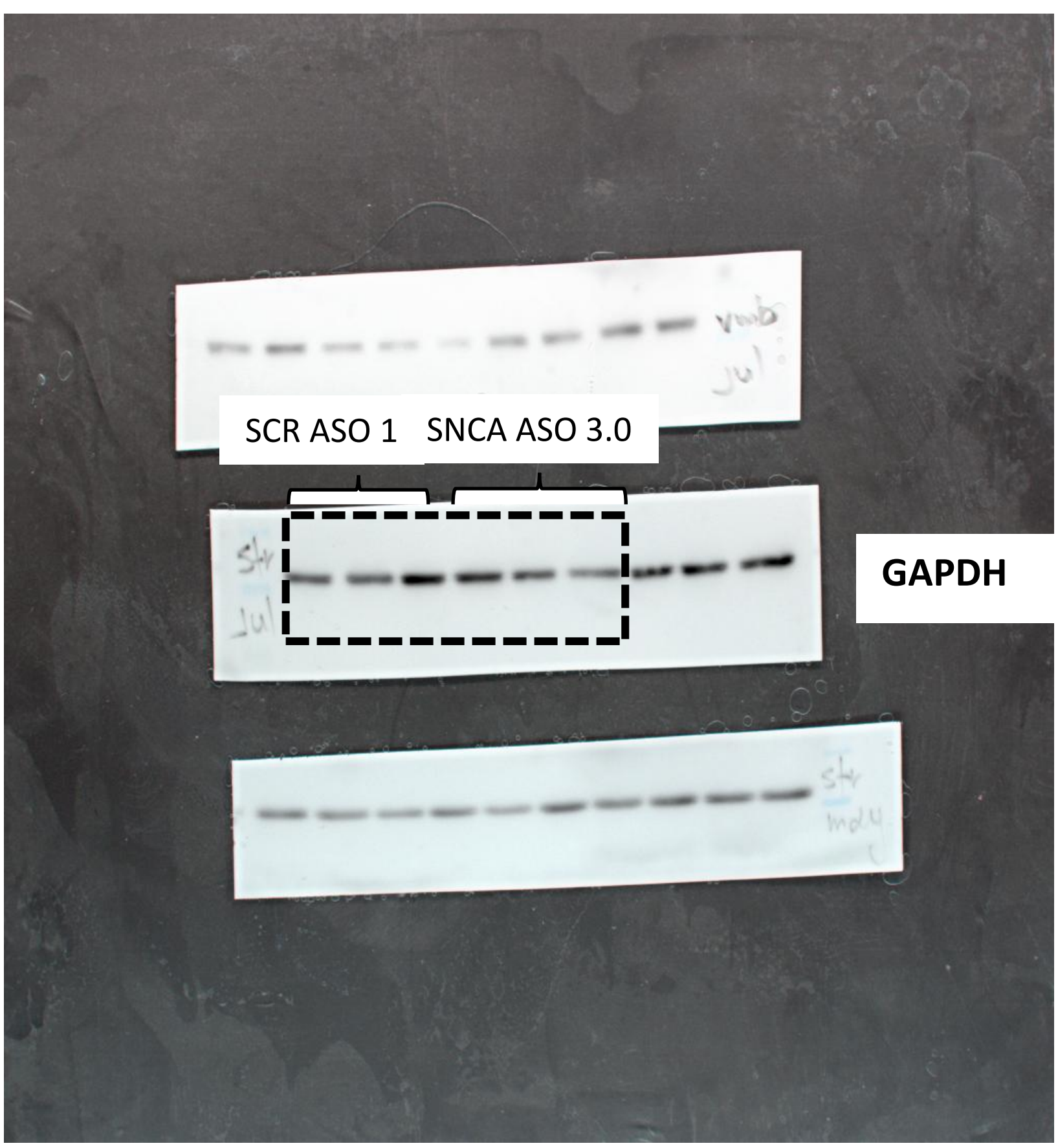

Figure S5

Striatum

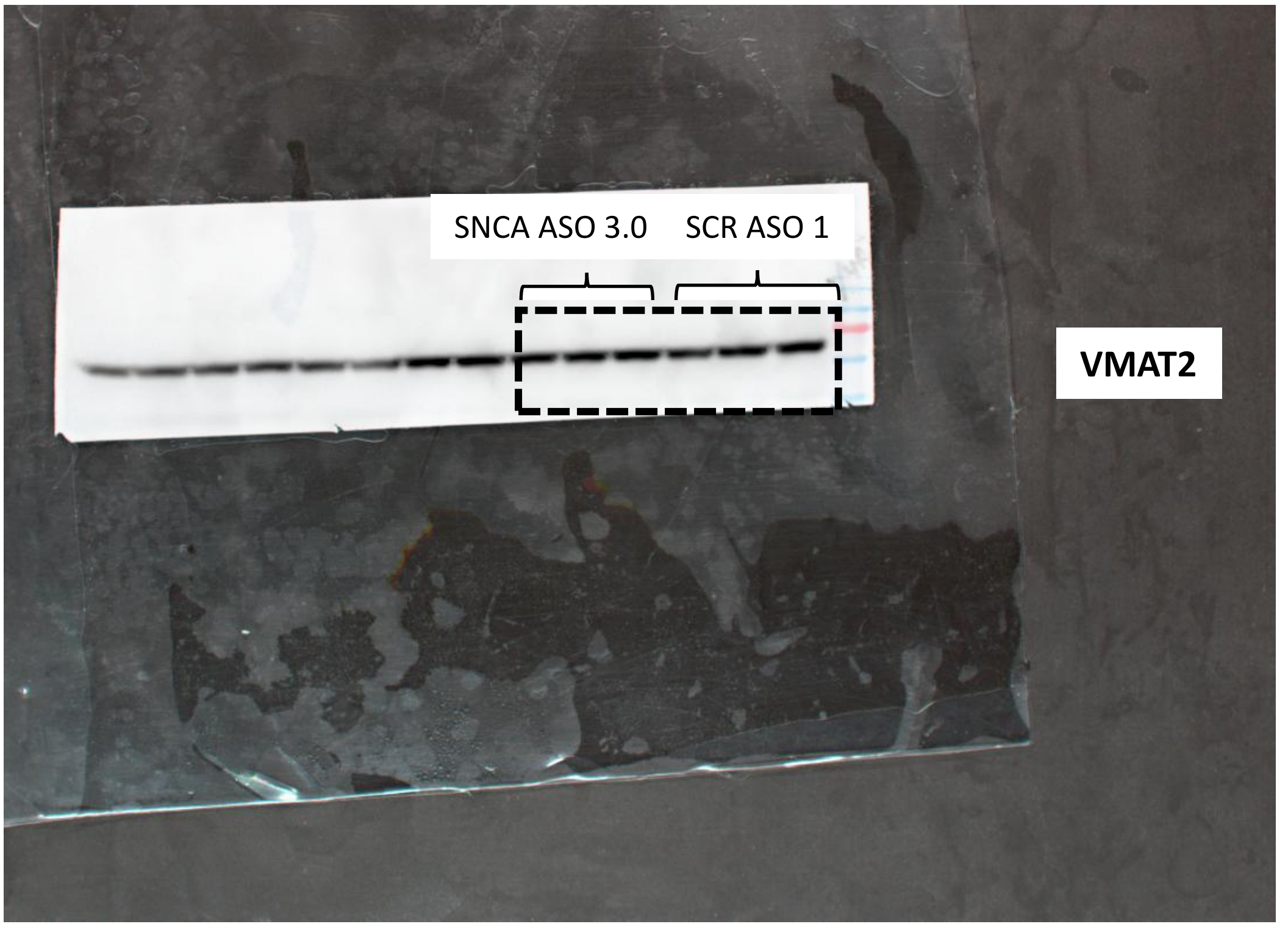

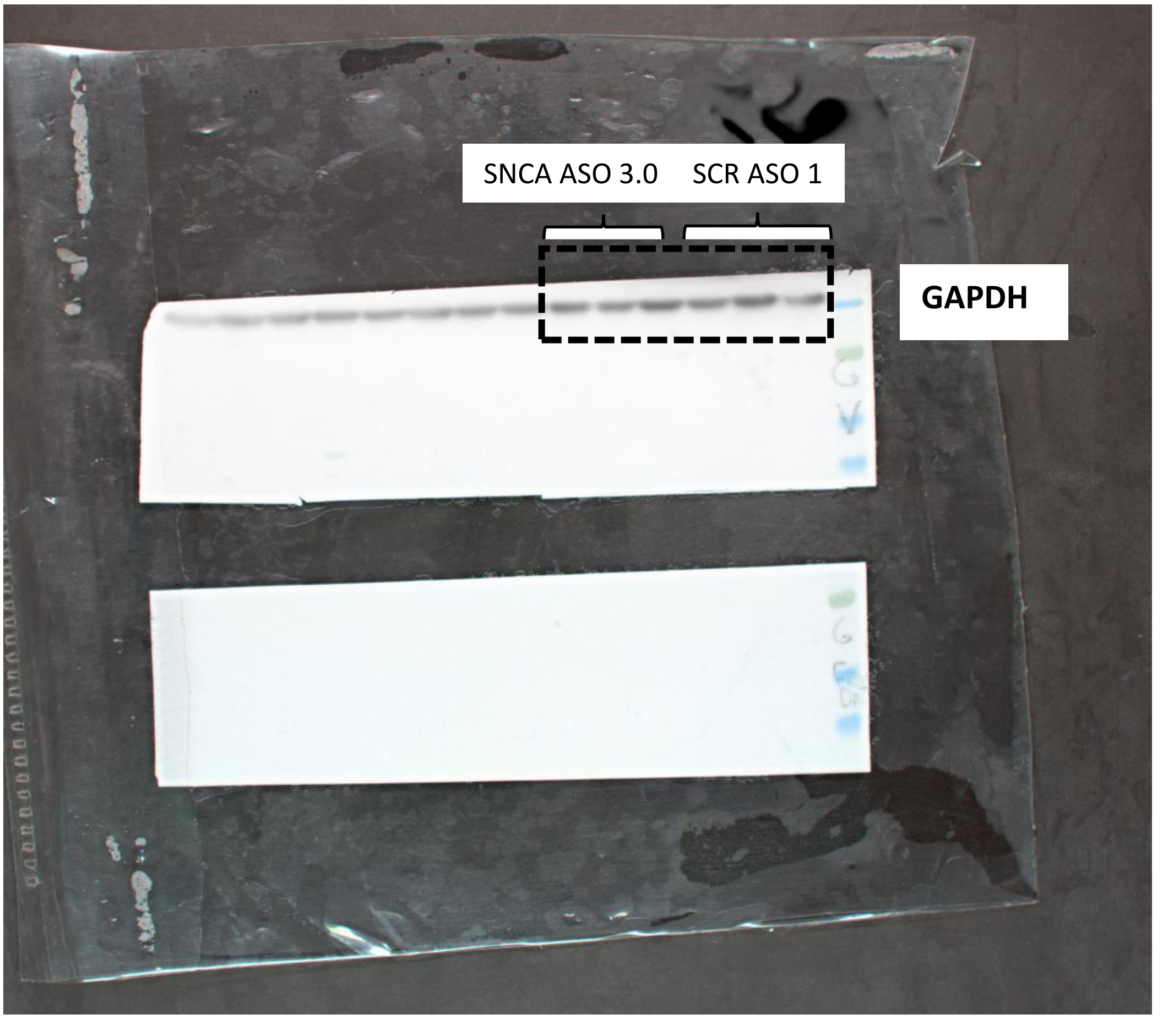

Figure S5

Ventral Midbrain

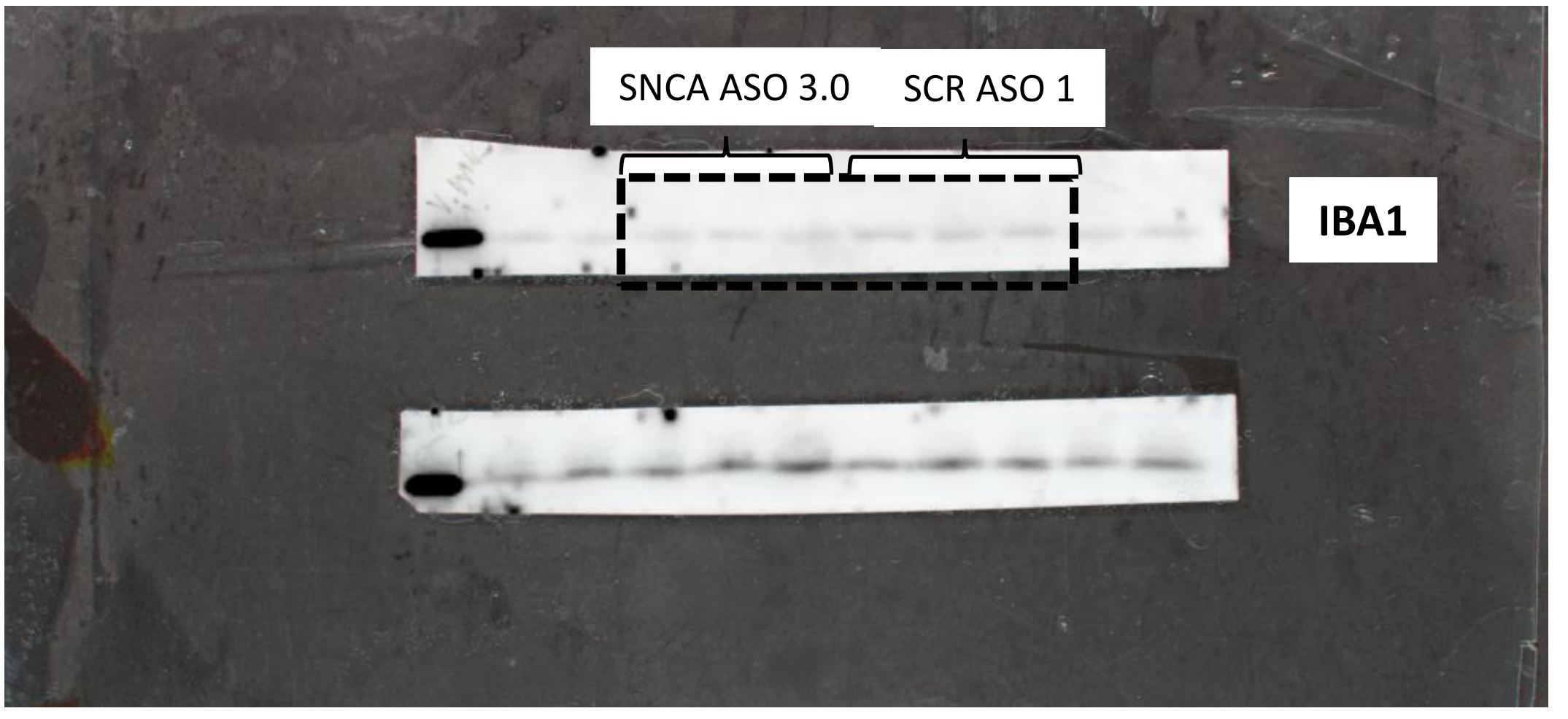

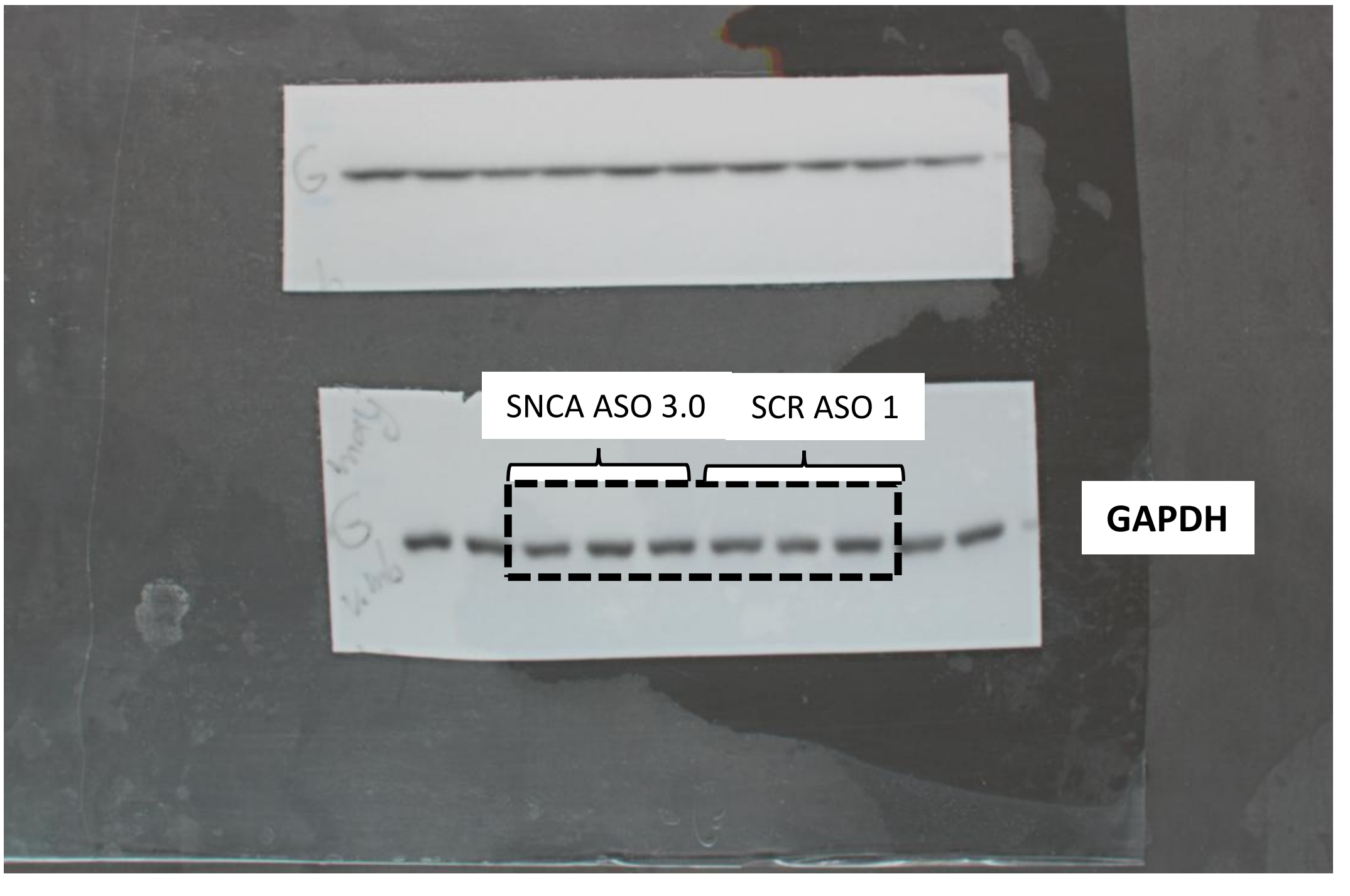

Figure S5

Ventral Midbrain

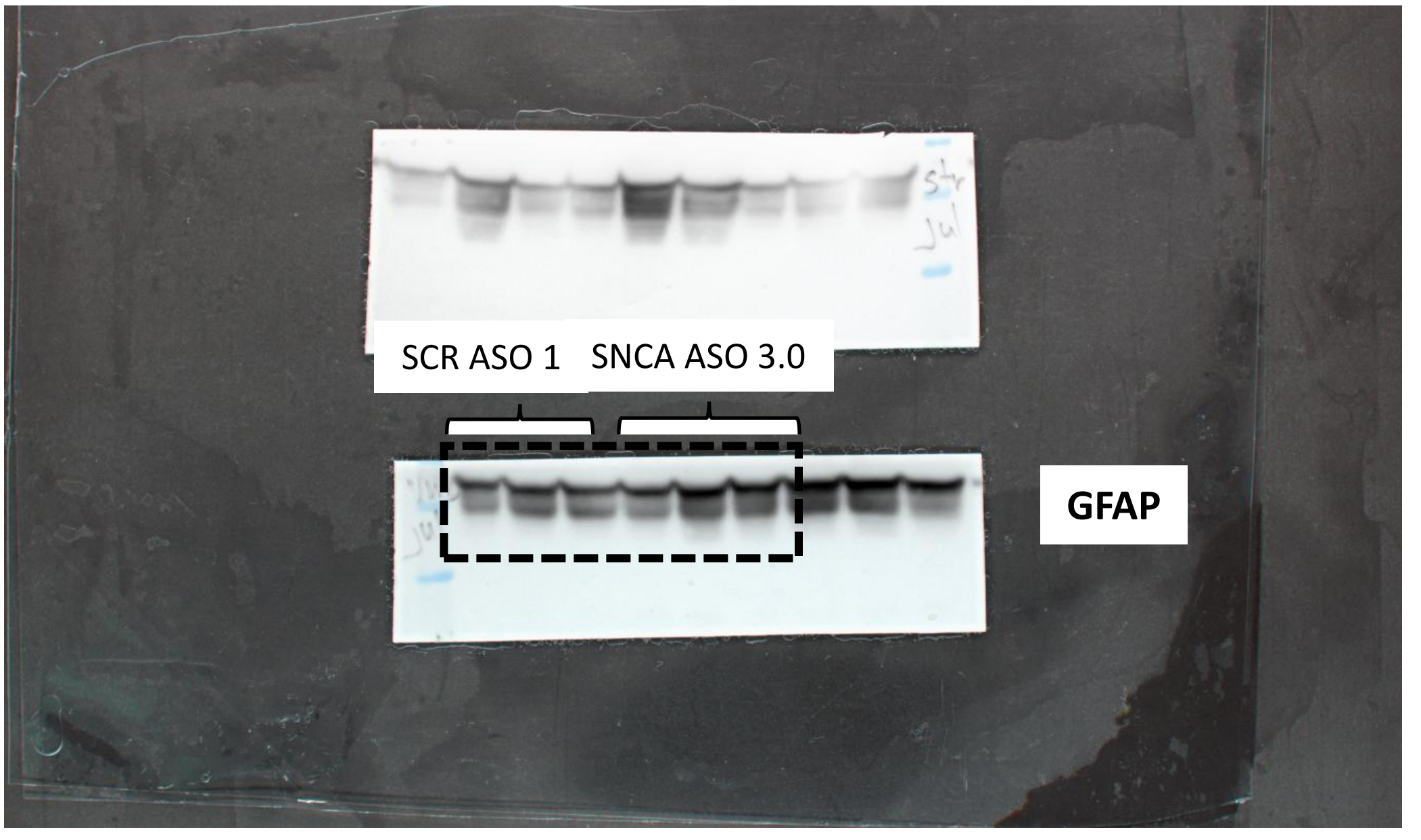

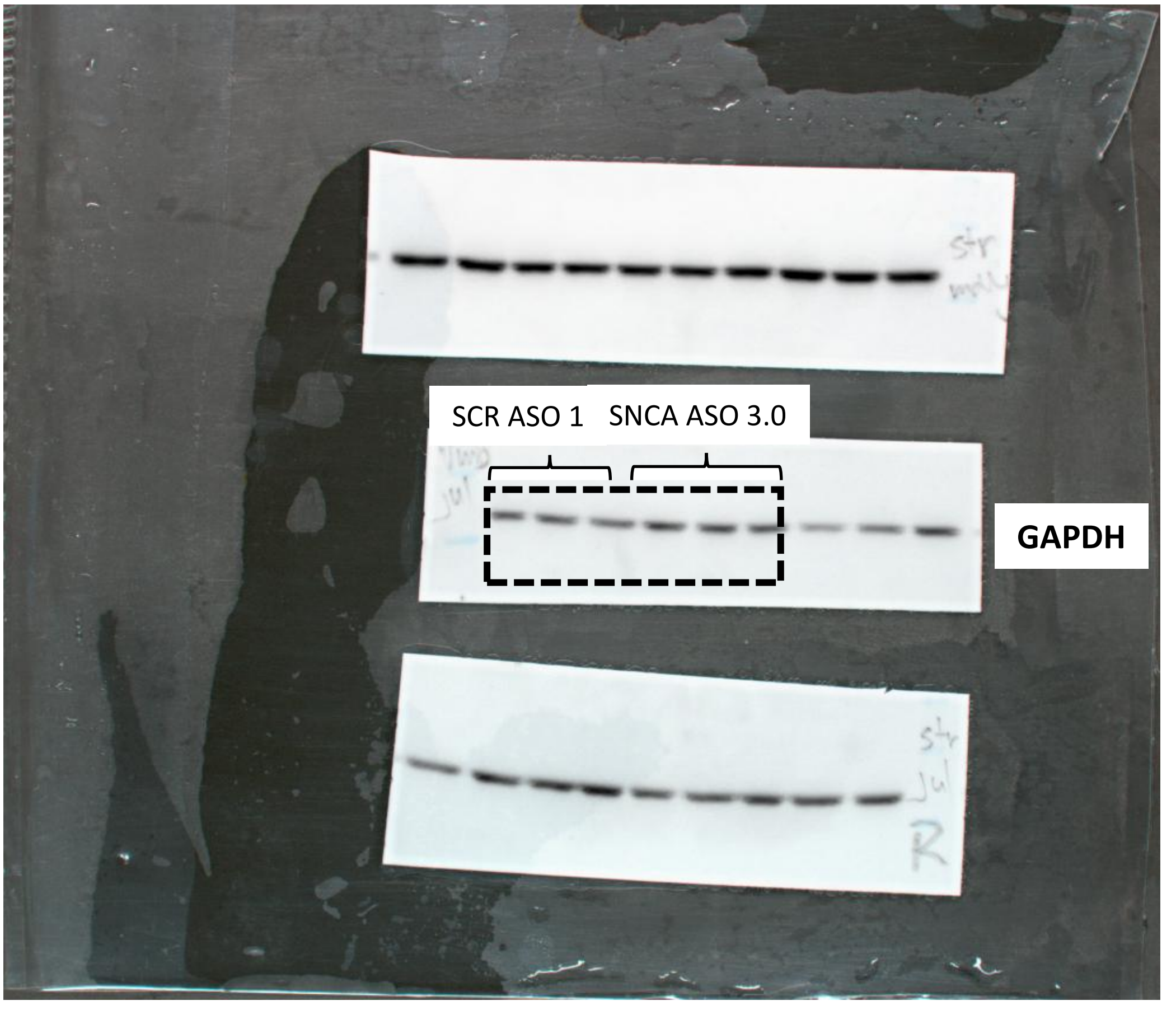

Supplement: Supplementary file 1 [file 40035_2026_577_MOESM5_ESM.pdf]
