# Supplementary material for: Brain region-resolved pharmacodynamics of an antisense oligonucleotide targeting human SNCA 3′UTR in BAC-hSNCA rats
Source: Transl Neurodegener. 2026 Sep 16;15:47. doi: 10.1186/s40035-026-00577-x (PMC13579807; doi:10.1186/s40035-026-00577-x)
Supplement: Supplementary file 2 [file 40035_2026_577_MOESM1_ESM.docx]

**Additional Figures**

**Figure S1. Representative visualization of intracerebroventricular injectate spread using Fast Green FCF.** Coronal sections collected 30 min after Fast Green injection show **a** the injection site, **b** spread in the mid-rostral forebrain, and **c** signal in the cerebellum.

**Figure S2. Western Blots of α-synuclein protein expression across brain regions in WT and BAC-h*SNCA* rats.** Monomeric and higher molecular-weight α-synuclein species in the soluble fraction were assessed using the 4B12 (GTX21904, 1:1000, GeneTex) **a** and Syn1 (#610787, 1:1000, BD) **b** antibodies. 4B12 is human-reactive and yields a low band in wild-type rat tissue, consistent with partial cross-reactivity, with a markedly higher signal in BAC-h*SNCA* samples. Parallel Syn1 blots confirm endogenous rat α-synuclein (panel **b**).

**Figure S3. Transcript-level comparison of PBS and scrambled ASO1.** **a** Human *SNCA* mRNA levels across selected brain regions in PBS- and scrambled ASO1-treated animals. **b** Long-to-total *SNCA* 3′UTR transcript ratios in the same regions. Data represent individual animals with mean ± SD. FDR-adjusted *q < 0.05, **q < 0.01, ***q < 0.001, ****q < 0.0001. Statistical analysis: Two-way ANOVA (study group × brain region) followed by Benjamini–Hochberg FDR-adjusted post hoc comparisons between scrambled ASO1 and SNCA ASO3.0 within each region. PBS samples (*n* = 2) were included only for this transcript-level comparison. Scrambled ASO1 (*n* = 3) serves as the reference condition for all primary analyses in the main figures.

**Figure S4. Western blots of α-synuclein protein expression in BAC-h*SNCA* rats following ASO3.0 treatment.** Monomeric, serine-129-phosphorylated, and higher molecular-weight α-synuclein species in Triton X-100-soluble fractions, and monomeric and detergent-insoluble α-synuclein in SDS-solubilized fractions across brain regions. Alpha-synuclein immunoblots used the human-reactive 4B12 antibody.

**Figure S5. SNCA ASO3.0 treatment does not alter GFAP, IBA1, or VMAT2 readouts in BAC-h*SNCA* rats.** IBA1 protein levels in the striatum and ventral midbrain assessed by immunoblotting **a** and immunohistochemistry **b**. GFAP protein levels in the same regions assessed by immunoblotting **c** and immunohistochemistry **d**. Striatal VMAT2 protein levels assessed by immunoblotting **e** and immunohistochemistry **f**. Data represent mean ± SD (*n* = 3 per group). Statistical analysis: Two-way ANOVA (study group × brain region) followed by Benjamini–Hochberg FDR-adjusted post hoc comparisons between scrambled ASO1 and SNCA ASO3.0 within each region for panels **a-d**, and two-tailed Student’s t-test for panels **e-f**.

**Figure S6. Cytokines unaffected by SNCA ASO3.0 treatment in homozygous BAC-h*SNCA* rats.** Data represent mean ± SD from five biological replicates. Statistical analysis: Two-way ANOVA (study group × brain region) followed by Benjamini–Hochberg FDR-adjusted post hoc comparisons between scrambled ASO1 and SNCA ASO3.0 within each region.

**Figure S7. Kinases and kinase targets with no phosphorylation changes after ASO3.0 treatment.** The horizontal red line denotes a signal-to-noise ratio (SNR) of 3, the threshold used to consider the phosphoprotein signal reliably above assay-specific background. Values below this threshold are displayed for completeness but were not interpreted as reliable above-background signals. Data represent mean ± SD from five biological replicates (FDR-adjusted *q < 0.05, **q < 0.01, ***q< 0.001, ****q< 0.0001). Statistical analysis: Two-way ANOVA (study group × brain region) followed by Benjamini–Hochberg FDR-adjusted post hoc comparisons between scrambled ASO1 and SNCA ASO3.0 within each region.

**Table S1. Primer sequences used for cloning and RT-qPCR.**

| Name | Forward primer (5’→ 3’) | Reverse primer (5’→ 3’) |
| --- | --- | --- |
| SNCA_all isoforms assay | GAAATATCTTTGCTCCCAGT | ACATTGGAACTGAGCACTTG |
| SNCA_long isoform assay | TGACGGTAATTTTTGAGCAGTGAA | GCTTCCTGAAAAGGCTTATATGACTTAA |
| GAPDH assay | GCACCACCAACTGCTTAG | GCCATCCACAGTCTTCTG |
| SNCA_CDS  plasmid | GAATTGCGGCCCAACGGTACCACCGCCACCATGGATGTATTCATGAAAGGAC | TATCGATAAGCTTGATATCGAATTCTTAGGCTTCAGGTTCGTAGTC |
| SNCA_CDS+3UTR_1-371_  plasmid | ATCATTTTGGCAAAGAATTGGATCCACCGCCACCATGGATGTATTCATGAAAGG | CCAGAGGTTGATTATCGATAAGCTTCTAAAAATCTTATAATATATGATAGCAAATC |
| SNCA_CDS+3UTR_1-576_  plasmid | ATCATTTTGGCAAAGAATTGGATCCACCGCCACCATGGATGTATTCATGAAAGG | CCAGAGGTTGATTATCGATAAGCTTATATTTTTGCAATGAGATAACG |
| SNCA_CDS+3UTR_1-1083_  plasmid | ATCATTTTGGCAAAGAATTGGATCCACCGCCACCATGGATGTATTCATGAAAGG | CCAGAGGTTGATTATCGATAAGCTTTTTATTCATGGTCGAATATTATTTATTG |
| SNCA_CDS+3UTR_1-2529_  plasmid | ATCATTTTGGCAAAGAATTGGATCCACCGCCACCATGGATGTATTCATGAAAGG | CCAGAGGTTGATTATCGATAAGCTTTATTTTCATATATGTATATATTTATTATGTCAC |

**Table S2. Target proteins and phosphorylation sites included in the custom-developed 20-plex phosphoprotein assay panel.**

| **#** | **Protein-assay abbreviation** | **Protein Name** | **pResidue** |
| --- | --- | --- | --- |
| 1 | SMAD3 | Mothers against decapentaplegic homolog 3 | S423/S425 |
| 2 | p53 | Cellular tumour antigen p53 | S15 |
| 3 | AKT1S1 | Proline-rich AKT1 substrate 1 | T246 |
| 4 | GSK3 | Glycogen synthase kinase-3 alpha/beta | S21/9 |
| 5 | AKT1 | RAC-alpha serine/threonine-protein kinase | S473 |
| 6 | HSPB1 | Heat shock protein beta-1 | S78/S82 |
| 7 | p38 | Mitogen-activated protein kinase 14/11 | T180/Y182 |
| 8 | MEK1 | Dual specificity mitogen-activated protein kinase kinase 1 | S217/S221 |
| 9 | RSK1 | Ribosomal protein S6 kinase alpha-1 | S380 |
| 10 | CREB1 | Cyclic AMP-responsive element-binding protein 1 | S133 |
| 11 | IKBA | NF-kappa-B inhibitor alpha | S32/S36 |
| 12 | mTOR | mammalian target of rapamycin | S2448 |
| 13 | c-JUN | Transcription factor Jun | S63 |
| 14 | EGFR | Epidermal growth factor receptor | Y1068 |
| 15 | ERK1 | Mitogen-activated protein kinase 3 | T202/Y204 |
| 16 | PTPN11 | Tyrosine-protein phosphatase non-receptor type 11 | Y542 |
| 17 | STAT3 | Signal transducer and activator of transcription 3 | Y705 |
| 18 | CHK2 | Checkpoint kinase 2 | T68 |
| 19 | NFKB | Nuclear factor kappa-light-chain-enhancer of activated B cells | S536 |
| 20 | MARCKS | Myristoylated alanine-rich C-kinase substrate | S170 |
| 21 | FAK | Focal adhesion kinase | Y397 |

**Table S3. Target proteins included in the custom-developed 13-plex extracellular protein assay panel.**

| **#** | **Protein-assay abbreviation** | **Protein Name** |
| --- | --- | --- |
| 1 | MB | Myoglobin |
| 2 | CNTF | Ciliary neurotrophic factor |
| 3 | MCP3 | Monocyte chemotactic protein-3 |
| 4 | RETN | Resistin |
| 5 | MIP-3a | Macrophage Inflammatory Protein-3 alpha |
| 6 | MIP1b | Macrophage Inflammatory protein-1 beta |
| 7 | RAGE | Receptor for Advanced Glycation End products |
| 8 | b-NGF | Beta-nerve growth factor |
| 9 | MMP9 | Matrix metalloproteinase 9 |
| 10 | L-Selectin | L-Selectin |
| 11 | PAI-1 | Plasminogen activator inhibitor 1 |
| 12 | MDC | Macrophage-Derived Chemokine |
| 13 | SCF | Stem cell factor |

**Table S4. Target proteins included in the Bio-Plex Pro™ Rat Cytokine 23-Plex assay panel.**

| **#** | **Protein-assay abbreviation** | **Protein Name** |
| --- | --- | --- |
| 1 | IL-13 | Interleukin-13 |
| 2 | IL-10 | Interleukin-10 |
| 3 | IL-18 | Interleukin-18 |
| 4 | IL-1a | Interleukin-1 alpha |
| 5 | IL-2 | Interleukin-2 |
| 6 | M-CSF | Macrophage colony-stimulating factor |
| 7 | IL-1b | Interleukin-1 beta |
| 8 | IL-4 | Interleukin-4 |
| 9 | IFN-g | Interferon gamma |
| 10 | MIP-3a | Macrophage Inflammatory Protein-3 alpha |
| 11 | GM-CSF | Granulocyte-macrophage colony-stimulating factor |
| 12 | IL-7 | Interleukin-7 |
| 13 | TNF-a | Tumor necrosis factor alpha |
| 14 | VEGF | Vascular Endothelial Growth Factor |
| 15 | MCP-1 | Monocyte Chemoattractant Protein-1 |
| 16 | IL-5 | Interleukin-5 |
| 17 | G-CSF | Granulocyte colony stimulating factor |
| 18 | RANTES (CCL5) | C-C motif chemokine 5 |
| 19 | IL-6 | Interleukin-6 |
| 20 | GRO/KC (CXCL1) | Growth-regulated alpha protein |
| 21 | IL-17A | Interleukin-17A |
| 22 | MIP-1a | Macrophage Inflammatory protein-1 alpha |
| 23 | IL-12p70 | Interleukin-12 p70 |
